# Supplementary material for: Global use of electronic patient-reported outcome systems in nephrology: a mixed methods study
Source: BMJ Open. 2023 Jul 12;13(7):e070927. doi: 10.1136/bmjopen-2022-070927 (PMC10347510; doi:10.1136/bmjopen-2022-070927)

Supplementary file S1

Nephrology ePROs search strategy and PRISM ScR diagram

MEDLINE (R) (Ovid) 1946 to 15/12/21

Note: Sets 1 to 6 are based on the Oxford PROM Filter (1) and sets 9-11 and 13 were developed for systematic review exploring measurement properties of PROs used in CKD (2)

1. (HR-PRO or HRPRO or HRQL or HRQoL or QL or QoL).ti,ab.

2. quality of life.mp.

3. (health index* or health indices or health profile*).ti,ab.

4. health status.mp.

5. ((patient or self or child or parent or carer or proxy) adj (appraisal* or appraised or report or reported or reporting or rated or rating or based or assessed or assessment*)).ti,ab.

6. ((disability or function or functional or functions or subjective or utility or utilities or wellbeing or well being) adj2 (index or indices or instrument or instruments or measure or measures or questionnaire* or profile or profiles or scale or scales or score or scores or status or survey or surveys)).ti,ab.

7. ((((patient adj reported adj outcome adj measure*) or patient) adj reported adj outcome*) or capability or capabilities).mp. [mp=title, abstract, original title, name of substance word, subject heading word, keyword heading word, protocol supplementary concept word, rare disease supplementary concept word, unique identifier]

8. 1 or 2 or 3 or 4 or 5 or 6 or 7

9. (Renal replacement therapy or APD or Automated Peritoneal Dialysis or CAPD, Continuous Ambulatory Peritoneal Dialysis or CCPD or Continuous cyclic peritoneal dialysis or dialysis or h*emofiltration or h*emodiafiltration or h*emodialysis or kidney transplant* or predialysis or renal replacement or renal transplant*).mp.

10. (CRF or chronic renal failure or CKF or chronic kidney failure or kidney disease* or renal disease or kidney failure or renal failure or CKD or chronic kidney disease or ESKD or end stage kidney disease or ESKF or end stage kidney failure or ESRF or end stage renal failure or ESRD or end stage renal disease or kidney insufficiency).mp.

11. Renal Insufficiency, Chronic/

12. 9 or 10 or 11

13. (PRO integration or Clinical PRO application* or telePRO or automated PRO algorithm* or screening purpose* or PRO questionnaire* or Patient-reported outcome questionnaire* or Patient-reported symptom* or Patient-centred care or Patient self-report* or Self-report health or Self-rated health or Self-reported measure* of health or Health outcome* or Health communication* or Hospital performance evaluation* or Automated telephone survey system* or paper-based survey* or web-based survey* or web-based PRO platform* or web-based system* or PRO collection* or PRO measure* or PRO intervention* or PRO assessment intervention* or PRO data or PRO assessment* or Routine PRO assessment* or Routine PRO collection or Symptom assessment* or Symptom monitoring or Symptom data or Functional status or Electronic PRO assessment* or Electronic PRO system* or ePRO orePRO* or ePRO system* or PRO system* or Generic PRO system* or PRO-based clinical alert system*).mp.

14. 8 and 12 and 13

EMBASE (Ovid) 1974 to 15/12/21

Note: Sets 1-6 based on search strategy developed for systematic review exploring measurement properties of PROs used in CKD (2).

1. (Renal replacement therapy or APD or Automated Peritoneal Dialysis or CAPD, Continuous Ambulatory Peritoneal Dialysis or CCPD or Continuous cyclic peritoneal dialysis or dialysis or h*emofiltration or h*emodiafiltration or h*emodialysis or kidney transplant* or predialysis or renal replacement or renal transplant*).mp.

2. (CRF or chronic renal failure or CKF or chronic kidney failure or kidney disease* or renal disease or kidney failure or renal failure or CKD or chronic kidney disease or ESKD or end stage kidney disease or ESKF or end stage kidney failure or ESRF or end stage renal failure or ESRD or end stage renal disease or kidney insufficiency).mp.

3. (PRO integration or Clinical PRO application* or telePRO or automated PRO algorithm* or screening purpose* or PRO questionnaire* or Patient-reported outcome questionnaire* or Patient-reported symptom* or Patient-centred care or Patient self-report* or Self-report health or Self-rated health or Self-reported measure* of health or Health outcome* or Health communication* or Hospital performance evaluation* or Automated telephone survey system* or paper-based survey* or web-based survey* or web-based PRO platform* or web-based system* or PRO collection* or PRO measure* or PRO intervention* or PRO assessment intervention* or PRO data or PRO assessment* or Routine PRO assessment* or Routine PRO collection or Symptom assessment* or Symptom monitoring or Symptom data or Functional status or Electronic PRO assessment* or Electronic PRO system* or ePRO or ePRO* or ePRO system* or PRO system* or Generic PRO system* or PRO-based clinical alert system*).mp.

4. *patient/ or *outcome assessment/ or *questionnaire/ or *"quality of life"/ or *self report/

5. 1 or 2

6. 3 and 4 and 5

PsycINFO (Ovid) 1967 to 15/12/21

Note: Sets 1 to 6 are based on the Oxford PROM Filter (1). Sets 7, 9-11 and 13 based on search strategy developed for systematic review exploring measurement properties of PROs used in CKD (2)

1.(HR-PRO or HRPRO or HRQL or HRQoL or QL or QoL).ti,ab.

2.quality of life.mp. 103501

3.(health index* or health indices or health profile*).ti,ab.

4.health status.mp.

5.((patient or self or child or parent or carer or proxy) adj (appraisal* or appraised or report or reported or reporting or rated or rating or based or assessed or assessment*)).ti,ab.

6.((disability or function or functional or functions or subjective or utility or utilities or wellbeing or well being) adj2 (index or indices or instrument or instruments or measure or measures or questionnaire* or profile or profiles or scale or scales or score or scores or status or survey or surveys)).ti,ab.

7.((((patient adj reported adj outcome adj measure*) or patient) adj reported adj outcome*) or capability or capabilities).mp. [mp=title, abstract, heading word, table of contents, key concepts, original title, tests & measures, mesh word]

8.1 or 2 or 3 or 4 or 5 or 6 or 7

9.(Renal replacement therapy or APD or Automated Peritoneal Dialysis or CAPD, Continuous Ambulatory Peritoneal Dialysis or CCPD or Continuous cyclic peritoneal dialysis or dialysis or h*emofiltration or h*emodiafiltration or h*emodialysis or kidney transplant* or predialysis or renal replacement or renal transplant*).mp.

10.(CRF or chronic renal failure or CKF or chronic kidney failure or kidney disease* or renal disease or kidney failure or renal failure or CKD or chronic kidney disease or ESKD or end stage kidney disease or ESKF or end stage kidney failure or ESRF or end stage renal failure or ESRD or end stage renal disease or kidney insufficiency).mp.

11.*Organ Transplantation/ or *Hemodialysis/ or *Kidneys/ or *Kidney Diseases/ or Renal Insufficiency, Chronic.mp.

12.9 or 10 or 11

13.(PRO integration or Clinical PRO application* or telePRO or automated PRO algorithm* or screening purpose* or PRO questionnaire* or Patient-reported outcome questionnaire* or Patient-reported symptom* or Patient-centred care or Patient self-report* or Self-report health or Self-rated health or Self-reported measure* of health or Health outcome* or Health communication* or Hospital performance evaluation* or Automated telephone survey system* or paper-based survey* or web-based survey* or web-based PRO platform* or web-based system* or PRO collection* or PRO measure* or PRO intervention* or PRO

assessment intervention* or PRO data or PRO assessment* or Routine PRO assessment* or Routine PRO collection or Symptom assessment* or Symptom monitoring or Symptom data or Functional status or Electronic PRO assessment* or Electronic PRO system* or ePRO or ePRO* or ePRO system* or PRO system* or Generic PRO system* or PRO-based clinical alert system*).mp.

14.8 and 12 and 13

CINAHL (via EBSCO host) inception to 15/12/21

Sets 1 and 2 based on search strategy developed for systematic review exploring measurement properties of PROs used in CKD (2)

S1. (MH "Kidney Failure, Chronic/TH/TM/TD/SU/SS/RF/RH/PF/PR/PC/PP/PA/NU/MO/ME/ET/EI/EP/ED/EC/DT/DI/CO/CL") OR "((Renal replacement therapy or APD or Automated Peritoneal Dialysis or CAPD, Continuous Ambulatory Peritoneal Dialysis or CCPD or Continuous cyclic peritoneal dialysis or dialysis or h*emofiltration or h*emodiafiltration or h*emodialysis or kidney transplant* or predialysis or renal replacement or renal transplant*).mp.) OR ((CRF or chronic renal failure or CKF or chronic kidney failure or kidney disease* or renal disease or kidney failure or renal failure or CKD or chronic kidney disease or ESKD or end stage kidney disease or ESKF or end stage kidney failure or ESRF or end stage renal failure or ESRD or end stage renal disease or kidney insufficiency).mp.) OR (*Organ Transplantation/ or *Hemodialysis/ or *Kidneys/ or *Kidney Diseases/ or Renal Insufficiency, Chronic.mp.)"

S2. ((PRO integration or Clinical PRO application* or telePRO or automated PRO algorithm* or screening purpose* or PRO questionnaire* or Patient-reported outcome questionnaire* or Patient-reported symptom* or Patient-centred care or Patient self-report* or Self-report health or Self-rated health or Self-reported measure* of health or Health outcome* or Health communication* or Hospital performance evaluation* or Automated telephone survey system* or paper-based survey* or web-based survey* or web-based PRO platform* or web-based system* or PRO collection* or PRO measure* or PRO intervention* or PRO assessment intervention* or PRO data or PRO assessment* or Routine PRO assessment* or Routine PRO collection or Symptom assessment* or Symptom monitoring or Symptom data or Functional status or Electronic PRO assessment* or Electronic PRO system* or ePRO or ePRO* or ePRO system* or PRO system* or Generic PRO system* or PRO-based clinical alert system*).mp.)""

S3. S1 and S2

References

(1) PROM Group, A.M., Carolina Casañas i Comabella, Monica Hadi, Elizabeth Gibbons, Ray Fitzpatrick, Nia Roberts, *PROM GROUP CONSTRUCT & INSTRUMENT TYPE FILTERS.* 2010.

(2) Aiyegbusi, O. L., Kyte, D., Cockwell, P., Marshall, T., Gheorghe, A., Keeley, T., Slade, A., & Calvert, M. (2017). Measurement properties of patient-reported outcome measures (PROMs) used in adult patients with chronic kidney disease: A systematic review. *PloS one*, *12*(6), e0179733. https://doi.org/10.1371/journal.pone.0179733

PRISMA diagram

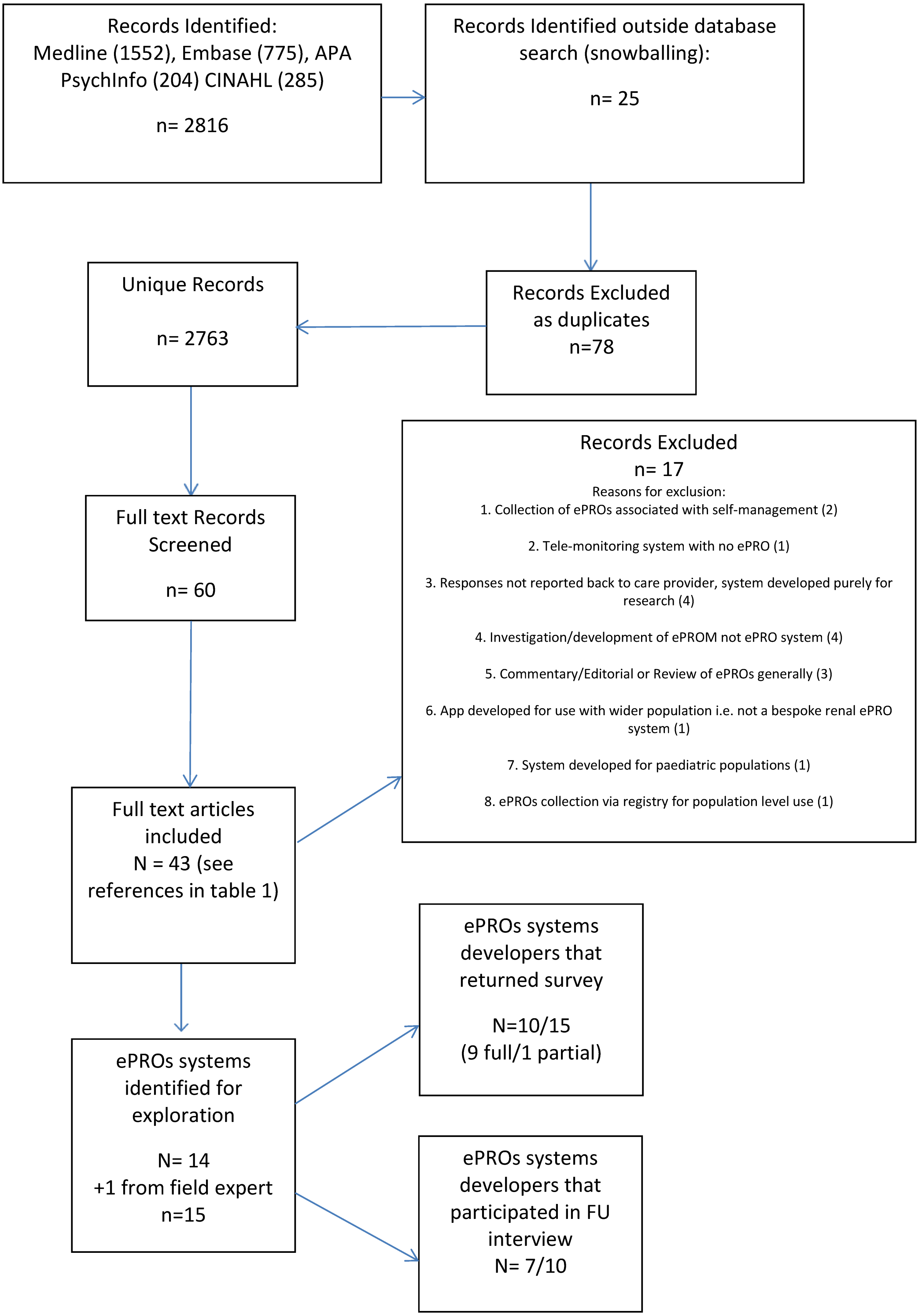

Supplement: Supplementary data [file bmjopen-2022-070927supp001.pdf]
