# Supplementary material for: Global use of electronic patient-reported outcome systems in nephrology: a mixed methods study
Source: BMJ Open. 2023 Jul 12;13(7):e070927. doi: 10.1136/bmjopen-2022-070927 (PMC10347510; doi:10.1136/bmjopen-2022-070927)
Supplement: Supplementary data [file bmjopen-2022-070927supp002.pdf]

Electronic Patient-Reported Outcomes (ePRO) systems used in nephrology settings

1. Electronic Patient-Reported Outcomes (ePRO) Systems used in Nephrology settings

Consent Form.

Please note you may choose to save and continue your survey response at a later time. If you choose this option at the end of each page, you will be directed to enter your email address and we will email you a link for you to access the survey in the future.

1. I confirm that I have read and understood the information in the Participant information sheet version 3.0 dated 06.06.2021 and understand what is expected of me. *

2. I confirm that I have been given the opportunity to ask questions regarding the study, and if asked, the questions were answered to my full satisfaction. *

3. I understand that my participation is voluntary and that I am free to withdraw at any time, but within 5 working days following survey completion (before my data is integrated into the dataset), without giving any reason. *

4. I understand that any personal information will be anonymized but institutional or system identifiers will be used in research outputs i.e. publications/presentations. *

5. I understand that the information collected may be used to support other research in the future and may be shared anonymously with other researchers. *

6. I understand that my personal data will be processed for the purposes detailed above, in accordance with the General Data Protection Regulation and Data Protection Act 2018 *

7. I agree to take part in the survey (please note, if you answer no to this question you will be taken to the end of this survey) *

8. OPTIONAL I am willing to take part in a follow up interview following survey completion, where required.
I understand:

The interview will be audio-recorded and that the transcript of the recording will be shared with researchers working on the above study at the University of Birmingham.

My participation in this interview is voluntary and that I am free to withdraw at any time, up to 10 working days following interview (before my data is integrated into the dataset), without giving any reason.

I will have the opportunity to review any interview transcript and redact any proprietary information before analysis.

I must return reviewed transcripts within 10 working days, otherwise it will be assumed I am happy with the contents.

*

9. Thank you very much for offering to take part in the the optional interview. Please enter your preferred contact details below and a member of the team will be in contact to arrange a convenient date and time. *

First name *

Last name *

Email *

3. ePRO System Characteristics

Survey

10. Please enter your name (this information is for administrative purposes only and your data will be pseudonymised) *

11. What was your job title during system development?

12. What is your system called?

Name

Version number

13. Please describe your key responsibilities in the ePRO system development/implementation, please include clinical and non-clinical responsibilities as appropriate.

14. Please state the purpose of your ePRO system (please tick those that apply, there may be more than one primary purpose)

	Primary objective of system	Secondary objective of system	Not a system objective
Improving symptom management (associated with underlying chronic kidney disease)	☐	☐	☐
Improving symptom management (associated with treatment monitoring e.g. Haemodialysis)	☐	☐	☐
Identification of psychosocial problems	☐	☐	☐
To facilitate patient-provider communication	☐	☐	☐
Research	☐	☐	☐
Benchmarking of services	☐	☐	☐
Clinical service commissioning	☐	☐	☐
Support transition of care e.g. to renal replacement therapy or conservative care	☐	☐	☐

If your ePRO system has another purpose, please state

15. What is the population focus of the system (please tick all that apply)

- ☐ Chronic Kidney Disease Stages 1-3
- ☐ Chronic Kidney Disease Stages 4-5 pre dialysis
- ☐ In-centre Haemodialysis
- ☐ Home Haemodialysis
- ☐ Peritoneal Dialysis
- ☐ Transplant
- ☐ Conservative Care

16. When was ePRO system launched?

DD/MM/YYYY

17. How was the system developed?

- ☐ Informatics team within the host organisation (single organisation)
- ☐ Collaboration across two or more organisations
- ☐ Already available Commercial product

Comments:

18. Approximate number of patient users

19. Approximate number of clinician users

20. Primary location of use (please tick all that apply)

- ☐ Primary Care (Community/General Practice clinic)
- ☐ Secondary Care (Hospital clinic)
- ☐ Dialysis Centre
- ☐ Home

21. What PRO Measure (s) are you using?

	PRO Measure
Are you using a new measure?	☐
Are you using an existing measure(s)?	☐
Do you need to use a licence for use?	☐

Comments: Please state name of PRO measure(s)

☐

22. Has the PRO Measure been validated with your patient population? *

- ☐ Yes
- ☐ No

23. How was your ePRO system validated?

- ☐ Formal paper-electronic equivalence testing
- ☐ None
- ☐ Other (please specify): ☐

24. What analysis metric is being used to make or inform clinical decisions?

- ☐ Change from baseline
- ☐ Final value
- ☐ Time to event
- ☐ Other (please specify): ☐

25. How was your ePRO system funded? (you may tick more than one option if mixed model funding used)

- ☐ Commercial
- ☐ Academic
- ☐ Charitable
- ☐ Governmental

Other: Please state

☐

26. What security features does your ePRO system employ? (please tick all that apply)

- ☐ Secure log in (password)
- ☐ Encryption
- ☐ Other (please specify):

6. Administration of PRO assessments/level of automation

27. Is your ePRO system web-based and/or App based (please tick all that apply)

- ☐ Non-responsive website i.e. for display on desk top computer
- ☐ Responsive/mobile website i.e. content displays and function across all devices
- ☐ Mobile App

28. How is the ePRO system accessed? (please tick all that apply)

- ☐ Computer
- ☐ Tablet
- ☐ Interactive Voice Response System
- ☐ Clinic based kiosk
- ☐ Smartphone
- ☐ Other (please specify):

29. Timing of ePRO assessment

- ☐ Prior to clinical assessment
- ☐ Set timepoint weekly
- ☐ Set timepoint monthly
- ☐ Set timepoint 3 monthly
- ☐ Set timepoint 6 monthly
- ☐ Set timepoint annually
- ☐ As required (by patient)
- ☐ Other (please specify):

30. PRO assessment selection: How does your ePRO system select which PRO to use?

☐ Automatic (by system) i.e. Provider
☐ Patient

Comments:

31. Question Format: How does the ePRO system present questions?

☐ One question per page
☐ Multiple questions per page
☐ Mixed format of single and multiple questions

32. Question advancement: how does the ePRO system move from one question to the next?

☐ Mouse click
☐ Automatic on completion

33. What page features are available? (please tick all that apply)

☐ Progress Bar
☐ Visual graphics i.e. graph, diagram, chart
☐ Other (please specify):

34. How does your ePRO system avoid missing data during completion? (Please tick all that apply)

☐ Allows multiple logins per assessment with automatic save function
☐ Allows multiple logins per assessment with save and return later function
☐ Allows not applicable (N/A) response
☐ Default response pre-selected (pre-populated neutral response)
☐ Reminders
☐ Other (please specify):

35. Is the ePRO available in more than one language? *

	Yes	No
Is the ePRO available in more than one language?	☐	☐

Comments: Please state available languages

7. Cross cultural validation

36. Has the ePRO measure been validated for use in multiple languages?

- ☐ Yes
- ☐ No

Comments: if yes, please specify languages

37. How does the ePRO system notify that assessments have been completed? (Please tick all that apply)

- ☐ Automated submission notification to patient
- ☐ Automated submission notification to clinicians
- ☐ Email notification to patient from clinical team following review of responses
- ☐ App based notification
- ☐ Other (please specify):

38. How are patients reminded to complete their ePRO assessment? (Please tick all that apply)

- ☐ Email
- ☐ Telephone call
- ☐ Text message: SMS
- ☐ Verbally
- ☐ By letter
- ☐ No reminder
- ☐ App based push notification

39. Does the ePRO system offer flexible system features (please tick all that apply)

- ☐ Web based home and clinic login access
- ☐ Multiple assessment scheduling options
- ☐ Two or more sources for PRO selection (patient/provider)

- ☐ Self identification of important issues by patient (computer adaptive testing functionality)
- ☐ Free text availability
- ☐ Multiple language availability
- ☐ In app or push notifications
- ☐ Other (please specify):

9. ePRO System integration and reporting

40. Please outline the ePRO report content (Please tick all that apply)

- ☐ Current scores including summary and individual scores
- ☐ Longitudinal change
- ☐ Interpretation included in report
- ☐ Cut scores (e.g. low, medium, high)
- ☐ Population norms or reference values
- ☐ Identification of meaningful change
- ☐ Modifiable reports
- ☐ General guidelines
- ☐ Other (please specify):

41. How are ePRO reports accessed? (Please tick all that apply)

- ☐ Via clinical portal/electronic patient health record
- ☐ Via immediate access i.e. summary print out on completion
- ☐ Results restricted to clinical encounter
- ☐ Other (please specify):

42. Who has access to ePRO results/summary reports (please tick all that apply)

- ☐ Patient
- ☐ Clinicians
- ☐ Care Provider
- ☐ Multiple care provider access
- ☐ Other (please specify):

43. Who is responsible for initial review and action of ePRO results?

- ☐ Medical staff
- ☐ Nursing staff
- ☐ Administrative staff
- ☐ Other member of multidisciplinary team

Comments:

44. What form does the clinical response to ePRO report take? (please tick all that apply)

- ☐ Prescribed electronic response dependent on score
- ☐ Clinician/staff follow up; follow up type (face to face, virtual) dependent on score/decision aids
- ☐ Automatic referral to member of multidisciplinary team
- ☐ Automated patient education/message regardless of score
- ☐ Other (please specify):

45. Is there visual presentation of the ePRO scores? (please tick all that apply)

- ☐ Graphical
- ☐ Tabular
- ☐ Numerical
- ☐ Emoticon
- ☐ Colour coded
- ☐ Other (please specify):

46. Does your ePRO system have a safety alert system, dependent on respondent scores? *

- ☐ Yes
- ☐ No

10. Management of safety

47. Management of safety: Who is the intended primary recipient and audience of the ePRO score alert and associated response?

- ☐ Clinician/Staff
- ☐ Patients
- ☐ Care provider
- ☐ Caregiver
- ☐ Multiple recipients i.e. clinicians and patients

Comments:

48. Management of safety: what form do ePRO score alerts take? (please tick all that apply)

- ☐ Email
- ☐ Text message/SMS
- ☐ Telephone call
- ☐ Verbal
- ☐ Real time alert
- ☐ In App notification
- ☐ None

11. Training

49. Do you offer training on the ePRO system? *

- ☐ Yes
- ☐ No

12. Training

50. Who receives training on the ePRO system? (Please tick all that apply)

- ☐ Clinical staff
- ☐ Administrative staff
- ☐ Patients
- ☐ Carers
- ☐ Training available but not specified
- ☐ No formal training available

51. What form does ePRO system training take? (Please tick all that apply)

- ☐ Face to face
- ☐ Online
- ☐ Integrated into ePRO system
- ☐ No formal training

52. Is there a facility for patient education through the ePRO system? (please tick all that may apply)

- ☐ Patient education administered within system
- ☐ Education linked to ePRO scores
- ☐ Automatic documentation of actions
- ☐ No patient education offered

Comments:

13. Evaluation

53. Has your ePRO system been evaluated? *

14. Evaluation

54. What form of evaluation did you undertake?

- ☐ Formative research
- ☐ Pre implementation feasibility testing
- ☐ Progress focused evaluation concurrent to implementation
- ☐ Retrospective evaluation

Comments:

55. Did you include patients in your evaluation?

56. What, if any, changes did you make following the evaluation process

15. Additional features

57. Is there linkage between the ePRO system and other electronic data systems? (Please tick all that apply)

- ☐ Patient Electronic Health Record
- ☐ Appointment/scheduling systems
- ☐ Patient Billing systems
- ☐ No linkage
- ☐ Other (please specify):

58. Accessibility: is the data available to areas outside nephrology settings?

- ☐ Yes
- ☐ No

Comments: If yes, please state

59. Does your ePRO system offer any additional features (please tick all that apply)

- ☐ Quality of care evaluation
- ☐ Patient satisfaction data collected
- ☐ Accreditation reporting
- ☐ None
- ☐ Other (please specify):

60. Is IT support available for your ePRO system?

- ☐ Full
- ☐ Limited
- ☐ User Manual available
- ☐ None

Comments: if Limited, please state what support is offered

16. Contact details

61. Would you like a summary of the findings of this survey?

- ☐ Yes
- ☐ No

If yes, please enter your preferred contact details below

Thank you very much for taking part in this survey.
Would you like to get in touch with the research team?
If you would like any more information, please contact Nicola Anderson at NEA451@student.bham.ac.uk
