# Supplementary material for: Global use of electronic patient-reported outcome systems in nephrology: a mixed methods study
Source: BMJ Open. 2023 Jul 12;13(7):e070927. doi: 10.1136/bmjopen-2022-070927 (PMC10347510; doi:10.1136/bmjopen-2022-070927)
Supplement: Supplementary data [file bmjopen-2022-070927supp003.pdf]

Nephrology ePRO systems review - Topic Guide

Introduction/Ice breaker

The introduction will set the tone and start building rapport:
Introduce self as a Nurse Researcher with a clinical background specialising in nephrology research and that this interview is being undertaken as part of a clinician doctoral research fellowship funded by the National Institute for Health Research.

Tell respondents the general purpose of the interview

Before the interview, check that the respondents are still happy to take part and have signed the study specific electronic consent form.

Participants will be reminded that all individual self-identifiers will be removed before transcripts are analysed but that the System/institution itself will be named.

1. Icebreaker question: Please outline your role and key responsibilities as a System Lead for the ePRO outlined in your survey responses

2. Can you describe how the ePRO is being implemented in your institution?
Prompts: What was/is your implementation plan? What stage are you at?

3. What is the purpose of assessment?
Prompt: To inform clinical care, identify symptoms/tailor care, audit, benchmark, real-world evidence, trials Mixture?

4. Did you use internal/external change agents to help implementation?
Did you use peer support/champions to help implementation?
Prompt: Did you get endorsement or support from leaders? If yes, what form did it take, if no, why do you think not?

5. Did you use an existing PROM? Or develop a new measure?
Prompt: Did you measure the psychometric properties of any existing measure? If a new measure was created – what methodological approach was taken? Use of core outcome sets? COSMIN assessment? Level of patient input? FDA guidance?

6. Who are the key influential stakeholders for the ePRO? What was your engagement strategy to get these people on board?
Prompts: Stakeholders could be Health care professionals, patients, carers, senior managers, administration staff. Were these individuals involved in c0-designing the system – in particular were patients involved?

7. What is the general level of receptivity in your organization?
Prompt: Are users (patients/Healthcare Professionals) happy to use the ePRO,

Version 4.0 dated 11.06.2021

are they confident? If not, do you know why not?

8. Were any supports developed to help you implement and use the ePRO?
Prompts: Training resources? Marketing material?> toolkit? Were these resources available digitally, was face to face training made available.

9. What is the priority for getting the ePRO implemented relative to other initiatives that are happening now?
Prompt: for example was it affected by the COVID 19 pandemic?

10. Was the ePRO piloted prior to full-scale implementation?
Prompt: Can you describe the context of the pilot? What worked well? What did not work well?

11. Was the ePRO available in more than one language? Were there mechanisms to allow use by as many patients as possible?
Prompt: were any translations culturally validated?

12. What mechanisms do you have in place to allow maximum accessibility?
Prompt: was the ePRO available for use with patients who have impairments, or can't access IT

13. What direct and indirect costs were incurred to implement the ePRO?
Prompts: Did you need to make changes to infrastructure i.e. IT systems, staffing to support ePRO, adaptions to existing workflow and care delivery systems

14. Did you have sufficient resources to implement the ePRO?
Prompt: if not, what should be prioritised? Links to EPR? Safety features?

15. Do you think the ePRO has improved the way care is being delivered? If yes, how do you think this takes effect?
Prompt: Improved access to services? Help with self-management?, Reduced travel time and expense? To support individual care/tailor care to individual needs, audit, benchmarking, real-world evidence generation – all of these? Other

16. Are you collecting any information to assess impact of the ePRO? If so what type of data?
Prompt: health economic data to assess cost effectiveness, average length of consultation , hospitalisations, death rate, health service interactions, patient and clinician satisfaction

17. What kind of performance measures, policies, regulations, or guidelines influenced the decision to implement the ePRO? Does it provide your organisation with an advantage?
Prompt: Local, state, national measures? Were there incentives to support the

Version 4.0 dated 11.06.2021

ePRO

18. Overall has the ePRO been implemented according to the implementation plan?
    Prompt: What worked well (Facilitators), what did not (Barriers), what would you change?

19. Are you aware of other ePROs systems in regular/systematic use in nephrology settings?
    Prompt: If yes, which systems and what are the associated institution? Do you know who the System Lead is?

20. How does your ePRO compare to other similar/existing ePROs in your setting?
    Prompt: Are you aware of any specific advantages/disadvantages of your digital PRO?

21. Do you have anything else to add?

We have now reached the end of the interview. Please be reminded that any personal identifiers will be removed but institutional and system identifiers will be used in any subsequent outputs. You will be given a copy of the transcript after the interview and will have 10 working days to inform the lead researchers of any redactions you wish to make or study withdrawal. Many thanks for your participation.

Version 4.0 dated 11.06.2021
