# Supplementary material for: Global use of electronic patient-reported outcome systems in nephrology: a mixed methods study
Source: BMJ Open. 2023 Jul 12;13(7):e070927. doi: 10.1136/bmjopen-2022-070927 (PMC10347510; doi:10.1136/bmjopen-2022-070927)
Supplement: Supplementary data [file bmjopen-2022-070927supp004.pdf]

Table S1: Overview of ePRO systems with additional data

Name of System	Country<br>No of patient users<br>Population	Primary objective of system<br><br>Main place of completion	In current use<br>Yes/No<br>Timing of assessment	System Measures and summary of measured domains or items
**ANZDATA SWIFT PROMs Module (1-5)**	Australia<br><br>Collection via national registry<br>143 dialysis centres<br>Target n=2400<br><br>In-centre haemodialysis	Improving symptom management associated with CKD and treatment<br>Research<br><br><br>Dialysis centres	Yes – pilot study reported, Cluster RCT in progress<br>3 monthly for 12 months	**SONG-HD fatigue measure** (6)<br>Three items that assess (1) the effect of fatigue on life participation, (2) tiredness, and (3) level of energy.<br><br>**IPOS-Renal** (7)<br>IPOS-Renal is a short measure , combining the most common symptoms (pain, shortness of breath, weakness, nausea, vomiting, poor appetite, constipation, sore/dry mouth, drowsiness, poor mobility, itching, difficulty sleeping, restless legs, changes in skin, diarrhoea) renal patients experience plus additional items from IPOS on concerns beyond symptoms, such as information needs, practical issues, family anxiety<br><br>**EuroQoL 5-Dimension 5-Level (EQ-5D-5L)** (8)<br>Measures quality of life across 5 domains: mobility, self-care, usual activities, pain/discomfort, and anxiety/depression. Each dimension is scored on a 5-level severity ranking that ranges from "no problems" through "extreme problems."
**cPRO-Collaborate Kidney Care (9)**	United States of America<br>Single renal unit<br><br>n= approximately 350<br>CKD Stages 1-3<br>CKD Stages 4-5 pre dialysis	Improving symptom management associated with CKD, Patient-provider communication<br>Community/general practice/hospital clinic	Yes – clinical demonstration model<br>72 hours prior to clinical assessment visit and 3 monthly FU	**PROMIS and FACIT measures** (9)<br>Items from PROMIS and FACIT to measure: Health perception and global health, fatigue, anxiety, pain, depression, physical function, shortness of breath, urinary frequency, oedema, Appetite, itching, side effect bother, shared decision-making, self-efficacy managing treatments, self-efficacy managing symptoms, treatment satisfaction

**Cambian (ePRO-KIDNEY) (10-16)**	Canada<br>Regional<br><br>ePRO Kidney: n= 543 home dialysis patients<br><br>Home Haemodiaylsis, Peritoneal dialysis	Enhance and support person centred kidney care via research program on ePRO use and implementation<br><br>Secondary Care (Hospital clinic), Dialysis Centre<br>Home	No – platform used within reported research projects<br>Prior to clinical assessment<br>Set time point 3 monthly	**Patient Assessment of Care for Chronic Conditions-20 questionnaire (PACIC-20) (17, 18)**<br>Patient Assessment of Chronic Illness Care (PACIC) assesses the implementation of the chronic care model (CCM) from the patient perspective that focuses on the receipt of patient-centred care and self-management behaviours, this 20 item instrument has five pre-defined domains: patient activation (3 items), delivery system/practice design (3 items), goal setting/tailoring (5 items)<br><br>**Kidney Disease Quality of Life-36 (KDQOL-36) (19, 20)**<br>Based on the KDQOL-SF™. It includes the SF-12 as generic core plus the burden of kidney disease, symptoms/problems of kidney disease, and effects of kidney disease scales from the KDQOL-SF™v1.3.36 items, and two cores: the SF-12 (i.e., generic QOL) and the disease-specific core. The generic core consists of two domains: the physical component summary (PCS) [6 items] and the Mental Component Summary (MCS) [6 items]. The disease-specific core consists of 24 items with 3 domains: symptoms and problems (12 items), burden of kidney disease (4 items) and effects of kidney disease (8 items).<br><br>**Edmonton Symptom Assessment System renal (ESAS-r RENAL) (21)**<br>ESAS-R Renal 10 items (symptoms - pain, tiredness, drowsiness, nausea, lack of appetite, shortness of breath, depression, anxiety, well-being, other problem - Can be modified to include Restless Legs Syndrome single question, RLS defined as 'uncontrollable urge to move your legs')<br><br>**EQ-5D-5L (8)**<br>As above
**OPT-ePRO (22-25)**	United Kingdom<br>Collection via national registry across 3 renal units<br><br>Target n = Approx. 600 potential users	Improving symptom management associated with CKD and treatment, Research	No – pilot study reported<br>Prior to clinical assessment	**POS-S-RENAL (26)**<br>POS-S can also be used on its own, if only a symptom assessment is required, POS-S is available as a renal card - this version assesses 17 symptoms (symptoms (pain, shortness of breath, weakness, nausea, vomiting, poor appetite, constipation, sore/dry mouth, drowsiness, poor mobility, itching, difficulty sleeping, restless legs, changes in skin, diarrhoea, feeling anxious, feeling depressed), plus open fields to list main symptoms

	CKD Stages 1-3, CKD Stages 4-5 pre dialysis, In-centre HD, Home HD, PD Transplant, Conservative Care	HD units and outpatient clinics (Tx, PD, low clearance), home		**EQ-5D-5L (8)**<br>As above
**Penguin (Cievert Ltd)**<br>**(no published data)**	United Kingdom<br>Single renal unit<br><br>Pilot n=5<br><br>Transplant	Facilitate patient-provider communication<br><br>Secondary care – aim is to complete from home	Yes- Research pilot in progress<br>Prior to clinical assessment visit or annual review	**Check-up questionnaire (copy obtained from developer)**<br>Main concern, pain, breathing, Changes in urine output, swelling, skin/mouth problems, bone/joint problems, fertility (as well as domains on medications/appointments)<br><br>**Annual review (copy obtained from developer)**<br>Main problems, emotional health (Body changes, Mood swings)<br>Physical health (medication, activity, skin heath, smoking & drinking)<br>Sexual health and fertility
**RePROM (27-31)**	United Kingdom<br>Single renal unit<br><br>n=24<br><br>CKD Stages 4-5 pre dialysis	Improving symptom management associated with CKD and treatment<br><br>Secondary care (hospital clinic), home	No – pilot study reported.<br>Monthly reporting	**New study specific measure – RePROM (27)**<br>Items on fatigue; shortness of breath; loss of appetite; nausea or vomiting; itchiness or dry skin; pain; problems with fistula; faintness or dizziness; difficulty sleeping; restless legs; diarrhoea and ankle swelling. The questionnaire<br>also includes an open text item
**Unnamed System 1 (32)**	Canada<br>Single renal unit<br><br>n=approx. 60<br><br>In-centre HD, Transplant	Improving symptom management associated with CKD Research<br><br>Secondary care (hospital clinic), Dialysis centre	No – in development: pre-defined set time-points within research protocol	**PROMIS CATs (33) (unspecified)**<br>Previous pilot study to assess usability collected data from several measures (32)

**'Your symptoms matter' (34, 35)**	Canada<br>1 provincial region<br><br>n=1459<br><br>In-centre HD	Improving symptom management associated with CKD and treatment<br><br>Secondary Care (Hospital clinic), Dialysis Centre	Yes – pilot study reported<br>Every 4-6 weeks	**ESAS-r: Renal (21)**<br>As above
***Survey data only - developers declined participation in optional FU interview**				
**Ambuflex**<br>**(PRO-KID** Non-inferiority pragmatic randomised controlled trial evaluating incorporating Ambuflex platform where clinicians review responses)<br>**(36-41)**	Denmark<br>**Ambuflex** – National<br>**PRO-KID** – one hospital renal unit<br><br>Ambuflex<br>CKD Stages 4-5 pre dialysis, Conservative Care<br><br>PRO-KID<br>CKD Stages 4-5 pre dialysis	Improving symptom management associated with CKD Patient-provider communication<br><br>**Ambuflex**<br>Home<br><br>**PRO-KID -**<br>home	**Ambuflex** - Yes every 3rd month. PRO and laboratory tests inform clinicians whether the patient needs contact with outpatient clinic<br><br>**(PRO-KID** – trial in progress, every 3 months)	**Ambuflex** 27 item Renal Disease questionnaire (37)L including domains on kidney disease (Lack of appetite, food aversion, feeling of unease, nausea, vomiting, itchy skin, shortness of breath, swollen legs, dizziness, difficulty remembering, difficulty concentrating, restless legs, feeling tired, bodily pain, limited in regular daily activities, constipation, diarrhoea, nocturnal urination, trouble sleeping, worried about future health, difficulties remembering to take medication) and Quality of life (2 items), free text space for 3 topics to would like to discuss<br>**PRO-KID**<br>1. Renal-specific domains, items from • KDQOL-SF • EORTC • SF-GH1 • Need/wish for consultation • Free textbox<br>2. Additional research PROs not available to clinicians administered at baseline, 3, 6 and 18 months (36)
**SMaRRT-HD™ (42, 43)**	United States of America<br>Regional (clinic and dialysis unit)<br><br>N=62<br><br>In-centre HD	Improving symptom management associated with treatment<br><br>Dialysis Centre (Clinic + treatment area)	Unknown – feasibility study reported Twice monthly administered during first 30 minutes of HD (recall period of the last HD treatment for each symptom	**New Study specific measure - SMaRRT-HD™** (42)<br>Symptom Monitoring on Renal Replacement Therapy-Hemodialysis (SMaRRT-HD™) is a 14-item PROM intended for use in hemodialysis patents. SMaRRT-HD™ uses a single treatment recall period and a 5-point Likert scale to assess symptom severity across 13 Symptoms (12 specific: Cramping<br>Feeling washed out, Light headedness, Restless legs, Nausea, Vomiting, Headache, Tingling Thirst, Shortness of breath Chest pain, Heart palpitations Itching, 1 free text type in); time to recovery question

**System identified only – no survey or interview data included in evidence synthesis				
"Derby Evaluation of Illness" (44)	United Kingdom<br>Single renal unit<br><br>n=43 (19 HD, 5 PD, and 19 CKD)<br><br>CKD stages 4/5 pre-dialysis, in-centre HD, PD	Did not participate in survey<br><br>Home	Unknown – feasibility study reported<br>Daily (no reminders)	**New Study specific measure – unnamed (44)**<br>Six separate domains assessed by visual analogue scale (VAS): general wellbeing, pain, sleep, breathing, energy, fistula function and appetite.
EMPATHY study (45-48)	Canada<br>Three provincial regions<br><br>n=approx. 3,000<br><br>In-centre HD	Did not participate in survey<br><br>Questionnaires administered in-centre (precise timing not specified). PROMs are administered by pen and paper or through an electronic platform on a tablet in accordance with HD unit resources	Yes – Cluster RCT in progress<br>2 monthly	**ESAS-r: Renal (21)**<br>As above<br><br>**IPOS-Renal (7)**<br>As above<br><br>**And/or**<br>**EQ-5D-5L (8)**<br>As above<br>.
K-Pal (49)	United States of America<br>Single dialysis unit<br><br>n=22<br><br>Patient's ≥60 years of age with ESRD on HD	Did not participate in survey<br><br>During dialysis session (precise timing not specified)	Unknown – feasibility study reported<br>Baseline, 3 & 6 monthly	**Short-Form McGill Pain Questionnaire 2 (SF-MPQ-2)(50)**<br>The pain rating index has 2 subscales: Sensory subscale with 11 words, and affective subscale with 4 words from the original MPQ. Also one item for present pain intensity and one item for a 10 cm visual analogue scale (VAS) for average pain.[3] The SF-MPQ-2 includes 7 additional symptoms related to neuropathic pain, for a total of 22 items with 0-10 numerical response options<br><br>**Patient Health Questionnaire-9 (PHQ-9) (51)**<br>9 item questionnaire To measure depression severity and to diagnose depressive disorders

				**Generalized Anxiety Disorder 7 Item Survey (GAD-7) (52)**<br>7 item questionnaire exploring generalised anxiety<br><br>**Dialysis Symptom Index (DSI) (53)**<br>A 30-item measure designed to assess symptom prevalence and severity in patients on haemodialysis. 21 items relate to physical symptoms, 9 to emotional symptoms, plus open-ended question to report additional symptoms<br><br>**Kidney Disease Quality of Life (KDQOL-36) (19, 20)**<br>As above
**eNephro (54)**	France<br>Five Hospitals, 3 not for profit providers<br><br>Target stage 3B/4 (n = 320); stage 5D CKD on dialysis (n = 260); stage 5 T CKD treated with transplantation (n= 260).<br><br>CKD stage 3B/4, stage 5D CKD on dialysis (PD/HD), Transplant	Did not participate in survey<br><br>Home tele-monitoring	Unknown – Pragmatic RCT (anticipated date of study completion Dec 2018 NCTO 2082093)<br>Patient assessed symptoms reported every 3 days	**Patient assessed presence of symptoms**<br>including vertigo, short breath, fatigue, oedema, abdominal pain, deterioration of general health status<br><br>PROs to assess study outcomes:<br>**KDQOL-36** (19, 20)<br>As above<br><br>**Hospitalization Anxiety Depression Scale (HADS)** (55)<br>14 questions in total (seven covering depression and seven covering anxiety).<br><br>**ReTransQoL (for transplant patients)(56)**<br>A 45-item measure designed to assess QOL in renal transplant patients. There are 5 dimensions: (i) physical health (ii) social functioning (iii) medical care (iv) treatment (v) fear of losing graft
**Dutch Renal Registry (57-59)**	The Netherlands<br><br>Collection via national registry pilot study in 16 centres<br><br>Advanced CKD with and without renal replacement therapy	Did not participate in the survey<br><br>Home<br>During dialysis session (precise timing not specified)	Yes – part of development of national registry of PROMs<br>Baseline, 3 & 6 months during study period	**Dialysis Symptom Index (53)**<br>As above<br><br>**SF-12 (60)**<br>A generic HRQOL questionnaire: 12 items to measure HRQOL. Domains: Physical functioning (PF) (2 items), Role physical (RP) (2 items), Bodily pain (BP) (1 item), General health (GH) (1 item) Vitality (VT) (1 item) Social functioning (SF) (1 item) Role emotional (RE) (2 items) Mental health (MH) (2 items)

References

1. Duncanson E, Bennett PN, Viecelli A, Dansie K, Handke W, Tong A, et al. Feasibility and acceptability of e-PROMs data capture and feedback among patients receiving haemodialysis in the Symptom monitoring WIth Feedback Trial (SWIFT) pilot: protocol for a qualitative study in Australia. BMJ Open.10(11):e039014.
2. Morton RL, Lioufas N, Dansie K, Palmer SC, Jose MD, Raj R, et al. Use of patient-reported outcome measures and patient-reported experience measures in renal units in Australia and New Zealand: A cross-sectional survey study. Nephrology. 2020;25(1):14-21.
3. Viecelli AK, Duncanson E, Bennett PN, D'Antoine M, Dansie K, Handke W, et al. Perspectives of Patients, Nurses, and Nephrologists About Electronic Symptom Monitoring With Feedback in Hemodialysis Care. Am J Kidney Dis. 2022;80(2):215-26.
4. Greenham L, Bennett PN, Dansie K, Viecelli AK, Jesudason S, Mister R, et al. The Symptom Monitoring with Feedback Trial (SWIFT): protocol for a registry-based cluster randomised controlled trial in haemodialysis. Trials. 2022;23(1):419.
5. Duncanson EB, P. N.; Viecelli, A. K.; Dansie, K.; Greenham, L. M.; Tong, A.; Jesudason, S.; McDonald, S. P.; Morton, R. L. Mini Orals: Feasibility and acceptability of electronic patient reported outcome measures (e-proms) data capture in the symptom monitoring with feedback trial (SWIFT) pilot: Perspectives of nephrologists, nurses and patients. Nephrology. 2020;25:35.
6. Ju A, Teixeira-Pinto A, Tong A, Smith AC, Unruh M, Davison SN, et al. Validation of a Core Patient-Reported Outcome Measure for Fatigue in Patients Receiving Hemodialysis. The SONG-HD Fatigue Instrument. 2020;15(11):1614-21.
7. Raj R, Ahuja K, Fr, sen M, Murtagh FE, Jose M. Validation of the IPOS-renal symptom survey in advanced kidney disease: A cross-sectional study. Journal of Pain and Symptom Management. 2018;56(2):281-7.
8. Devlin NJ, Brooks R. EQ-5D and the EuroQol Group: Past, Present and Future. Appl Health Econ Health Policy. 2017;15(2):127-37.
9. Perry LM, Morken V, Peipert JD, Yanez B, Garcia SF, Barnard C, et al. Patient-Reported Outcome Dashboards Within the Electronic Health Record to Support Shared Decision-making: Protocol for Co-design and Clinical Evaluation With Patients With Advanced Cancer and Chronic Kidney Disease. JMIR Res Protoc. 2022;11(9):e38461.
10. Schick-Makaroff K, Molzahn A. Brief Communication: patient satisfaction wit the use of tablet computers: a pilot study in two outpatient home dialysis clinics. Canadian Journal of Kidney Health and Disease. 2014;1(22).
11. Schick-Makaroff K, Molzahn AE. Evaluation of real-time use of electronic patient-reported outcome data by nurses with patients in home dialysis clinics. BMC Health Serv Res. 2017;17(1):439.

12. Schick-Makaroff K, Mharapara P. Use of Electronic Patient-Reported Outcomes in the Care of Patients with Kidney Failure. Nephrology Nursing Journal: Journal of the American Nephrology Nurses' Association. 2020;47(5):465-72.

13. Schick-Makaroff K, Molzahn A. Strategies to use tablet computers for collection of electronic patient-reported outcomes. Health & Quality of Life Outcomes. 2015;13:2.

14. Schick-Makaroff K, Tate K, Molzahn A. Use of Electronic Patient Reported Outcomes in Clinical Nephrology Practice: A Qualitative Pilot Study. Can J Kidney Health Dis. 2019;6:2054358119879451.

15. Silbernagel P, Sawatzky R, Klarenbach S, Czupryn J, Iradukunda R, Palfreyman S, et al. Patient-Reported Outcomes in Clinical Kidney Practice: Development of Education Sessions for Clinicians. Journal of Pain and Symptom Management. 2018;56(6).

16. Schick-Makaroff K, Sawatzky R, Kwon JY, Lee L, Cohen SR, Czupryn J, et al. A process evaluation in home dialysis: Electronic patient reported outcomes in clinical kidney practice (ePRO Kidney). Quality of Life Research. 2022;31(Supplement 2):S67.

17. Glasgow RE, Wagner EH, Schaefer J, Mahoney LD, Reid RJ, Greene SM. Development and validation of the Patient Assessment of Chronic Illness Care (PACIC). Med Care. 2005;43(5):436-44.

18. Seo AR, Kim BK, Park KS. Psychometric Properties and Effects on Health Outcomes of the Patient Assessment of Chronic Illness Care (PACIC) in Korean Hemodialysis Patients. Healthcare. 2022;10(6):20.

19. Chao S, Yen M, Lin T-C, Sung J-M, Wang M-C, Hung S-Y. Psychometric Properties of the Kidney Disease Quality of Life–36 Questionnaire (KDQOL-36™). Western Journal of Nursing Research. 2016;38(8):1067-82.

20. Peipert JD, Bentler PM, Klicko K, Hays RD. Psychometric Properties of the Kidney Disease Quality of Life 36-Item Short-Form Survey (KDQOL-36) in the United States. Am J Kidney Dis. 2018;71(4):461-8.

21. Tang E, Dano S, Edwards N, Macanovic S, Ford H, Bartlett S, et al. Screening for symptoms of anxiety and depression in patients treated with renal replacement therapy: utility of the Edmonton Symptom Assessment System-Revised. Qual Life Res. 2021;31(2):597-605.

22. Van Der Veer SN, Ercia A, Caskey FJ, Farrington K, Jury F, Rees M, et al. Developing an Intervention to Implement Electronic Patient-Reported Outcomes in Renal Services in the UK. Studies in Health Technology & Informatics. 2020;270:936-40.

23. Knowles SE, Ercia A, Caskey F, Rees M, Farrington K, Van der Veer SN. Participatory co-design and normalisation process theory with staff and patients to implement digital ways of working into routine care: the example of electronic patient-reported outcomes in UK renal services. BMC Health Serv Res. 2021;21(1):706.

24. van der Veer SN, Couchoud C, Morton RL. The role of kidney registries in expediting large-scale collection of patient-reported outcome measures for people with chronic kidney disease. Clin Kidney J. 2021;14(6):1495-503.

25. van der Veer SN, Aresi G, Gair R. Incorporating patient-reported symptom assessments into routine care for people with chronic kidney disease. Clinical Kidney Journal. 2017;10(6):783-7.

26. Gutierrez-Sanchez D, Leiva-Santos JP, Sanchez-Hern, ez R, Hern, ez-Marrero D, et al. Spanish modified version of the palliative care outcome scale-symptoms renal: cross-cultural adaptation and validation. BMC Nephrology. 2016;17(1):180.
27. Kyte D, Anderson N, Auti R, Aiyegbusi OL, Bishop J, Bissell A, et al. Development of an electronic patient-reported outcome measure (ePROM) system to aid the management of patients with advanced chronic kidney disease. J Patient Rep Outcomes. 2020;4(1):55.
28. Aiyegbusi OL, Kyte D, Cockwell P, Marshall T, Dutton M, Slade A, et al. Using Patient-Reported Outcome Measures (PROMs) to promote quality of care and safety in the management of patients with Advanced Chronic Kidney disease (PRO-trACK project): a mixed-methods project protocol. BMJ Open.7(6):e016687.
29. Kyte D, Anderson N, Bishop J, Bissell A, Brettell E, Calvert M, et al. Results of a pilot feasibility randomised controlled trial exploring the use of an electronic patient-reported outcome measure in the management of UK patients with advanced chronic kidney disease. BMJ Open. 2022;12(3):e050610.
30. Kyte D, Bishop J, Brettell E, Calvert M, Cockwell P, Dutton M, et al. Use of an electronic patient-reported outcome measure in the management of patients with advanced chronic kidney disease: the RePROM pilot trial protocol. BMJ Open. 2018;8(10):e026080.
31. Aiyegbusi OL, Kyte D, Cockwell P, Marshall T, Dutton M, Walmsley-Allen N, et al. Development and usability testing of an electronic patient-reported outcome measure (ePROM) system for patients with advanced chronic kidney disease. Computers in Biology & Medicine.101:120-7.
32. Wong D, Cao S, Ford H, Richardson C, ice, Belenko D, et al. Exploring the use of tablet computer-based electronic data capture system to assess patient reported measures among patients with chronic kidney disease: a pilot study. BMC Nephrology. 2017;18:1-10.
33. Mucsi I, Chawla G, Chen A, Sanchez MJPM, Edwards N, Peipert JD, et al. Construct validity of the patient-reported outcomes measurement information system (promis) profile summary scores in patients with kidney failure. Journal of the American Society of Nephrology. 2021;32:632.
34. Evans JM, Glazer A, Lum R, Heale E, Mackinnon M, Blake PG, et al. Implementing a patient-reported outcome measure for hemodialysis patients in routine clinical care perspectives of patients and providers on esas-r:Renal. Clinical Journal of the American Society of Nephrology. 2020;15(9):1299-309.
35. Schell JO, Lupu DE. A step in the right direction the promise of proms in routine hemodialysis care. Clinical Journal of the American Society of Nephrology. 2020;15(9):1228-30.
36. Grove BE, Ivarsen P, de Thurah A, Schougaard LM, Kyte D, Hjollund NH. Remote follow-up using patient-reported outcome measures in patients with chronic kidney disease: the PROKID study - study protocol for a non-inferiority pragmatic randomised controlled trial. BMC Health Serv Res. 2019;19(1):631.

37. Grove BE, Schougaard LMV, Ivarsen PR, Kyte D, Hjollund NH, de Thurah A. Patient-reported outcome measures for clinical decision-making in outpatient follow-up: validity and reliability of a renal disease questionnaire. Journal of Patient-Reported Outcomes. 2021;5(1):1-11.
38. Schougaard L M LLP, Jessen A et al. AmbuFlex: tele-patient-reported outcomes (telePRO) as the basis for follow up in chronic and malignant diseases. Qual Life Res. 2016;25(3):525-34.
39. Hjollund NHI, Larsen LP, de Thurah AL, Grove BE, Skuladottir H, Linnet H, et al. Patient-reported outcome (PRO) measurements in chronic and malignant diseases: ten years' experience with PRO-algorithm-based patient-clinician interaction (telePRO) in AmbuFlex. Qual Life Res. 2023.
40. Grove BE, Valen Schougaard LM, Ivarsen P, Hjollund NH, de Thurah A, Mejdahl CT. Remote follow-up based on patient-reported outcomes in patients with chronic kidney disease: A qualitative study of patient perspectives. PLoS One. 2023;18(2):e0281393.
41. Grove B, Ivarsen P, De Thurah A, Kyte D, Henrik HN. Tele follow-up using patient-reported outcomes (PRO) measures in patients with chronic kidney disease-the PRO-KID study: A study protocol for a non-inferiority randomised controlled trial in Denmark. Quality of Life Research.27:S143-S4.
42. Flythe JE, Dorough A, Narendra JH, Wingard RL, Dalrymple LS, DeWalt DA. Development and content validity of a hemodialysis symptom patient-reported outcome measure. Qual Life Res. 2019;28(1):253-65.
43. Flythe JE, Tugman MJ, Narendra JH, Dorough A, Hilbert J, Assimon MM, et al. Feasibility of Tablet-Based Patient-Reported Symptom Data Collection Among Hemodialysis Patients. Kidney Int Rep. 2020;5(7):1026-39.
44. Pittman ZC, John SG, McIntyre CW. Collection of daily patient reported outcomes is feasible and demonstrates differential patient experience in chronic kidney disease. Hemodial Int. 2017;21(2):265-73.
45. Schick-Makaroff K, Wozniak LA, Short H, Davison SN, Klarenbach S, Buzinski R, et al. Burden of mental health symptoms and perceptions of their management in in-centre hemodialysis care: a mixed methods study. J Patient Rep Outcomes. 2021;5(1):111.
46. Johnson JA, Al Sayah F, Buzinski R, Corradetti B, Davison SN, Elliott MJ, et al. A cluster randomized controlled trial for the Evaluation of routinely Measured PATient reported outcomes in HemodialYsis care (EMPATHY): a study protocol. BMC Health Services Research. 2020;20(1):1-14.
47. Davison SN, Klarenbach S, Manns B, Schnick-Makaroff K, Buzinski R, Corradetti B, et al. Patient-reported outcome measures in the care of in-centre hemodialysis patients. Journal of Patient-Reported Outcomes. 2021;5(1):1-8.
48. Schick-Makaroff K, Wozniak LA, Short H, Davison SN, Klarenbach S, Buzinski R, et al. How the Routine Use of Patient-Reported Outcome Measures for Hemodialysis Care Influences Patient-Clinician Communication: A Mixed-Methods Study. Clin J Am Soc Nephrol. 2022;17(11):1631-45.

49. Gabbard J, McLouth CJ, Brenes G, Claudel S, Ongchuan S, Burkart J, et al. Rapid Electronic Capturing of Patient-Reported Outcome Measures in Older Adults With End-Stage Renal Disease: A Feasibility Study. Am J Hosp Palliat Care. 2021;38(5):432-40.

50. Dworkin RH, Turk DC, Revicki DA, Harding G, Coyne KS, Peirce-Sandner S, et al. Development and initial validation of an expanded and revised version of the Short-form McGill Pain Questionnaire (SF-MPQ-2). Pain. 2009;144(1-2):35-42.

51. Kroenke K, Spitzer RL, Williams JB. The PHQ-9: validity of a brief depression severity measure. J Gen Intern Med. 2001;16(9):606-13.

52. Spitzer RL, Kroenke K, Williams JBW, Löwe B. A Brief Measure for Assessing Generalized Anxiety Disorder: The GAD-7. Archives of Internal Medicine. 2006;166(10):1092-7.

53. Weisbord SD, Fried LF, Arnold RM, Rotondi AJ, Fine MJ, Levenson DJ, et al. Development of a symptom assessment instrument for chronic hemodialysis patients: the Dialysis Symptom Index. Journal of Pain & Symptom Management. 2004;27(3):226-40.

54. Thilly N, Chanliau J, Frimat L, Combe C, Merville P, Chauveau P, et al. Cost-effectiveness of home telemonitoring in chronic kidney disease patients at different stages by a pragmatic randomized controlled trial (eNephro): rationale and study design. BMC Nephrology. 2017;18(1):126.

55. Snaith RP. The Hospital Anxiety And Depression Scale. Health and Quality of Life Outcomes. 2003;1(1):29.

56. Gentile S, Jouve E, Dussol B, Moal V, Berland Y, Sambuc R. Development and validation of a French patient-based health-related quality of life instrument in kidney transplant: the ReTransQoL. Health Qual Life Outcomes. 2008;6:78.

57. van der Willik EM, Hemmelder MH, Bart HAJ, van Ittersum FJ, Hoogendijk-van den Akker JM, Bos WJW, et al. Routinely measuring symptom burden and health-related quality of life in dialysis patients: first results from the Dutch registry of patient-reported outcome measures. Clin Kidney J. 2021;14(6):1535-44.

58. van der Willik EM, Meuleman Y, Prantl K, van Rijn G, Bos WJW, van Ittersum FJ, et al. Patient-reported outcome measures: selection of a valid questionnaire for routine symptom assessment in patients with advanced chronic kidney disease - a four-phase mixed methods study. BMC Nephrol. 2019;20(1):344.

59. van der Willik EM, Milders J, Bart JAJ, Bos WJW, van Ittersum FJ, Ten Dam M, et al. Discussing results of patient-reported outcome measures (PROMs) between patients and healthcare professionals in routine dialysis care: a qualitative study. BMJ Open. 2022;12(11):e067044.

60. Ware J, Jr., Kosinski M, Keller SD. A 12-Item Short-Form Health Survey: construction of scales and preliminary tests of reliability and validity. Med Care. 1996;34(3):220-33.
