# Supplementary material for: Global use of electronic patient-reported outcome systems in nephrology: a mixed methods study
Source: BMJ Open. 2023 Jul 12;13(7):e070927. doi: 10.1136/bmjopen-2022-070927 (PMC10347510; doi:10.1136/bmjopen-2022-070927)
Supplement: Supplementary data [file bmjopen-2022-070927supp005.pdf]

Example illustrative quotations

**Intervention characteristics**	*'huge variety of different PROMs available but no single PROM measured even 50% of the symptoms that were reported by the population. So I think that kind of sends a clear message that maybe there's a gap here for a PROM that's maybe a bit more comprehensive that meets patient's needs. But of course, the challenge of that is you don't want to make it too long or burdensome.' (01)* – **Heterogeneity of measures**<br><br>*'the main learning is that if we want to make ePRO collection a success in renal or in any other specialty, we need to find concrete decisions or concrete situations where ePRO information really adds value. My learning is that just coming ePROs as a concept and saying, 'Now we're going to collect this data for anything,' I think that's not the way to go '(09).* **Importance of context and purpose**<br><br>*'It's a huge investment to make ePROS part of a registry and create that extra platform, and so we need to really provide the evidence that it's going to work before we just outlay everything, don't we?' (010)* – **Evidence of impact & benefit**
**Outer setting**	*'The way we designed the study was that we had intentionally iterations planned. We start with a certain package, then look at what works and what doesn't work and then add stuff. So related to what I said at the beginning, I think we started off with focusing on the patient side of things but acknowledging that we would need to do something to improve clinician engagement later on. So, I think I've always envisaged that being part of the later iterations, but we never got there because the study got paused halfway through the second wave' (09)* – **Impact of COVID**<br><br>*'It's really like hospital IT department, we kind of conceived of them as like a vendor who we were purchasing their services to launch something, which I guess is good, really wanted to be full partners, but that means they brought in their whole ideas about what informatics kind of launches should be like and this stuff. Which they were included in the co-production from the very beginning, which was good, but that relationship still isn't totally as good as it could be and so that's a challenge too……. there's always sort of push and pull on the priorities (02)* – **Partnerships and competing interests**<br><br>*'we were really planning to use this existing framework because this platform is integrated and linked to the local EPR and so once patients complete the questionnaires, their scores are displayed for the team' (04)* – **Data linkage**
**Inner setting**	*'And that was one thing that we had thought of going into it, was that any time you have a new sort of feature that's going to be in the clinic, it can actually exacerbate disparities because the patients who are most ready to benefit from that, who are already doing the best probably will, right? And so now they've got this new thing that they can use and the patients that are already doing worse or have the most challenges or barriers, that's just another thing that they're not going to be able to take advantage of…' (02)* – **Accessibility and inclusivity**

	'I think giving the people in these units the opportunity to fill out a survey in their own language also then increases the inclusivity of the trial' (010) - **Accessibility and inclusivity**<br><br>*But they [patients] did use words like feeling empowered to be a partner in the discussion. And obviously bringing up things, they had permission to bring things up and so it would be easier to start a conversation on mental health for example '(012)* – **shifting power to patients**<br><br>*And if I was going to do it over again, I would have taken all the emphasis off the clinicians, and I would have put it on the patients. I would have educated the patients. I would have told them how they could use it. Initiating discussion with the clinician. Giving it to them to share with their clinician or, like I would have turned the tables (03*) **– shifting power to patients**<br><br>*'They [clinicians] felt that when you have the ePRO data in advance of a consultation it allowed them to focus in on the really important aspects around patient symptoms from the outset so maybe more efficient use of time' (01)* – **fitting into workflow**
**Characteristics of individuals**<br><br>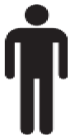	*'we also want to link the whole assessment to response pathways, both self-management support for patients and decision support for the professional teams' (04)* – **Ability to deal with responses**<br><br>*'But I think what I'm resisting is the, to quote Foucault, which is about the technology of self which is going to put it in a box. And say, this is not so bad, this is okay. This is a screaming alert and so the screaming alert gets all of our attention and all of sudden we've 'technologicalised' a life. And we don't want to do that so, I'm worried about moving to the technological simplification of something that isn't simple……….Yes, particularly in our context right, particularly where we see high technological dependence primarily in the nursing role…' (03)* – **Danger of digitising the patient experience**
**Process**<br><br>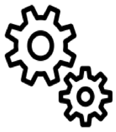	*'so the registry is a really important tool. We have this huge platform where we collect this information and it's already put in there in real time for the most part' (010)* – **Pragmatic approach**<br><br>*'The patient advisory group was really important especially during development. There are a number of times where we sort of went in with a plan and for the intervention, for the ePRO and we changed our plans based on what they were telling us in terms of how this would actually work in practice' (01)* – **Importance of patient involvement**<br><br>*'They got face-to-face training because they needed to understand the system really well and they needed to be able to explain what certain things meant. We did train nurses but that was more in groups. We maybe joined a multidisciplinary team meeting or a team meeting for the nurses in the morning, explaining where they could find the results' (09)* – **Value of training**

	'I guess the sustainability is the question because you can envision training and so-called patient [involvement] at the launch of this initiative and hoping that it will be cascaded down further and sustained but probably some kind of ongoing training or at least continuing education in services for nurses is going to be necessary. That's also part of a sustainability plan. It needs to become part of the quality systems (04) **– Sustainability**
