# Supplementary material for: Global use of electronic patient-reported outcome systems in nephrology: a mixed methods study
Source: BMJ Open. 2023 Jul 12;13(7):e070927. doi: 10.1136/bmjopen-2022-070927 (PMC10347510; doi:10.1136/bmjopen-2022-070927)
Supplement: Author's manuscript [file bmjopen-2022-070927.draft_revisions.pdf]

BMJ Open is committed to open peer review. As part of this commitment we make the peer review history of every article we publish publicly available.

When an article is published we post the peer reviewers' comments and the authors' responses online. We also post the versions of the paper that were used during peer review. These are the versions that the peer review comments apply to.

The versions of the paper that follow are the versions that were submitted during the peer review process. They are not the versions of record or the final published versions. They should not be cited or distributed as the published version of this manuscript.

BMJ Open is an open access journal and the full, final, typeset and author-corrected version of record of the manuscript is available on our site with no access controls, subscription charges or pay-per-view fees (http://bmjopen.bmj.com).

If you have any questions on BMJ Open's open peer review process please email
info.bmjopen@bmj.com

BMJ Open

Global Use of Electronic Patient-Reported Outcome Systems in Nephrology: A Mixed-Methods Study

Journal:	*BMJ Open*
Manuscript ID	bmjopen-2022-070927
Article Type:	Original research
Date Submitted by the Author:	13-Jan-2023
Complete List of Authors:	Anderson, Nicola; University of Birmingham, Centre for Patient Reported Outcomes Research; University Hospitals Birmingham NHS Foundation Trust, Renal Research<br>Kyte, Derek; University of Worcester,<br>McMullan, Christel; University of Birmingham, Institute of Applied Health Research, Centre for Patient Reported Outcomes Research<br>Cockwell, Paul; Queen Elizabeth Hospital Birmingham, Department of Renal Medicine; University of Birmingham, Division of Infection and Immunity<br>Aiyegbusi, Olalekan Lee; University of Birmingham, Centre for Patient-Reported Outcomes Research; University of Birmingham, NIHR Birmingham Biomedical Research Centre, University of Birmingham and University Hospitals Birmingham NHS Foundation Trust<br>Verdi, Rav; University of Birmingham, Patient Advisory Group Member, Centre for Patient-Reported Outcomes Research (CPROR)<br>Calvert , Melanie ; University of Birmingham, Centre for Patient Reported Outcomes Research and Institute of Applied Health Research; University of Birmingham, NIHR Birmingham Biomedical Research Centre
Keywords:	Telemedicine < BIOTECHNOLOGY & BIOINFORMATICS, Health informatics < BIOTECHNOLOGY & BIOINFORMATICS, Dialysis < NEPHROLOGY, Quality of Life, REGISTRIES

SCHOLARONE™
Manuscripts

I, the Submitting Author has the right to grant and does grant on behalf of all authors of the Work (as defined in the below author licence), an exclusive licence and/or a non-exclusive licence for contributions from authors who are: i) UK Crown employees; ii) where BMJ has agreed a CC-BY licence shall apply, and/or iii) in accordance with the terms applicable for US Federal Government officers or employees acting as part of their official duties; on a worldwide, perpetual, irrevocable, royalty-free basis to BMJ Publishing Group Ltd ("BMJ") its licensees and where the relevant Journal is co-owned by BMJ to the co-owners of the Journal, to publish the Work in this journal and any other BMJ products and to exploit all rights, as set out in our licence.

The Submitting Author accepts and understands that any supply made under these terms is made by BMJ to the Submitting Author unless you are acting as an employee on behalf of your employer or a postgraduate student of an affiliated institution which is paying any applicable article publishing charge ("APC") for Open Access articles. Where the Submitting Author wishes to make the Work available on an Open Access basis (and intends to pay the relevant APC), the terms of reuse of such Open Access shall be governed by a Creative Commons licence – details of these licences and which Creative Commons licence will apply to this Work are set out in our licence referred to above.

Other than as permitted in any relevant BMJ Author's Self Archiving Policies, I confirm this Work has not been accepted for publication elsewhere, is not being considered for publication elsewhere and does not duplicate material already published. I confirm all authors consent to publication of this Work and authorise the granting of this licence.

For peer review only

Global Use of Electronic Patient-Reported Outcome Systems in Nephrology: A Mixed Methods Study

Nicola Anderson MSc, RN Dip HE[1,2], Derek Kyte PhD[3] Christel McMullan PhD[1,4.5], Paul Cockwell PhD[7], Olalekan L Aiyegbusi PhD [1,5,6,8,9] Ravinder Verdi [10], Melanie J Calvert PhD[1,4,5,6,8,9]

[1] Institute of Applied Health Research, Centre for Patient Reported Outcomes Research, University of Birmingham, Birmingham, United Kingdom

[2]Research and Development, University Hospitals Birmingham NHS Foundation Trust

[3] School of Allied Health and Community, University of Worcester, Worcester, UK

[4] NIHR SRMRC, University Hospitals Birmingham NHS Foundation Trust and University of Birmingham, UK

[5]NIHR Birmingham-Oxford Blood and Transplant Research Unit (BTRU) in Precision Transplant and Cellular Therapeutics

[6]NIHR Birmingham Biomedical Research Centre, University Hospitals Birmingham NHS Foundation Trust and University of Birmingham, UK

[7]Department of Nephrology, University Hospitals Birmingham NHS Foundation Trust

[8]Birmingham Health Partners Centre for Regulatory Science and Innovation

[9]NIHR Applied Research Collaboration West Midlands, University of Birmingham

[10] Patient Partner, University of Birmingham, UK

Corresponding author: Nicola Anderson NEA451@bham.ac.uk

For peer review only - http://bmjopen.bmj.com/site/about/guidelines.xhtml

ABSTRACT

Objectives

The use of electronic patient-reported outcome (ePRO) systems to support the management of patients with chronic kidney disease is increasing. This mixed-methods study aimed to comprehensively identify existing and developing ePRO systems, used in nephrology settings globally, ascertaining key characteristics and factors for successful implementation.

Study design

ePRO systems and developers were identified through a rapid scoping review of the literature and contact with field experts. Developers were invited to participate in a structured survey, to summarise key system characteristics including: (i) system objectives; (ii) population; (iii) PRO measures used; (iv) level of automation; (v) reporting; (vi) integration into workflow; and (vii) links to electronic health records/national registries. Subsequent semi-structured interviews were conducted to explore responses.

Setting and participants

Eligible systems included those being developed or used in nephrology settings to assess ePROs and summarise results to care providers. System developers included those with a key responsibility for aspects of the design, development, or implementation of an eligible system.

Analytical Approach

Structured survey data was summarised using descriptive statistics. Interview transcripts were analysed using Codebook Thematic Analysis using domains from the Consolidated Framework for Implementation Research.

For peer review only - http://bmjopen.bmj.com/site/about/guidelines.xhtml

Results

Fifteen unique ePRO systems were identified across seven countries; ten system developers completed the structured survey and seven participated in semi-structured interviews. Despite system heterogeneity, reported features required for effective implementation included early and sustained patient involvement, clinician champions, and expanding existing electronic platforms to integrate ePROs. Systems demonstrated several common features, with the majority being implemented within research settings thereby affecting system implementation readiness for real-world application.

Conclusions

There has been considerable research investment in ePRO systems. Study findings may accelerate the implementation gap between research and practice by outlining key system features and factors to support the successful implementation of ePROs in routine kidney care.

For peer review only

Key Words:

Nephrology, Chronic Kidney Disease, Haemodialysis, Patients Reported Outcomes, Electronic Patient Reported Outcomes, Health Related Quality of Life

Article Summary

Strengths and limitations of this study

- This study summarises for the first-time characteristics of ePRO systems used globally to support the management of chronic kidney disease (CKD).
- The mixed-methods approach offered a greater understanding of the barriers and facilitators of ePROs implementation, supplementing survey findings on key system characteristics.
- The review was restricted to English language publications of ePRO systems. Therefore, it is possible that potentially relevant publications published in other languages were excluded.
- It is possible that other ePRO systems that could be used in nephrology settings were not identified by this study.

For peer review only

INTRODUCTION

Patients at all stages of chronic kidney disease (CKD), and particularly those undertaking kidney replacement therapy, experience a high symptom burden and often report diminished health-related quality of life (HRQOL) (1-3). However, some symptoms remain under-recognized and unalleviated, leading to increased patient burden (4). HRQOL, is not only an important outcome in itself, it is associated with clinical outcomes such as healthcare utilisation and mortality (5). Symptom burden and impact on HRQOL can be assessed through the use of patient-reported outcomes (PROs), defined as "any report of the status of a patient's health condition that comes directly from the patient, without interpretation of the patient's response by a clinician or anyone" (6). PROs assessing the impact of CKD and associated treatments can be collected electronically (ePROs).

With the increase in telemedicine, accelerated by the COVID-19 pandemic (7, 8), the collection of ePROs has increasing significance. Web-based systems and 'bring your own device' schemes offer patients new ways to report symptoms, function, and HRQOL; they can report PROs in 'real-time' from home or in clinic prior to their consultation. Such use could assist in the management of symptoms, whilst ensuring that healthcare resources are used effectively to maximize impact (9).

There is increasing evidence, particularly from oncology, that ePROs are cost-effective (10), can facilitate shared-decision making, promote self-management, improve symptoms and HRQOL, reduce unplanned hospitalisation and enhance long-term outcomes including survival (11-14). In contrast to paper-based collection, ePRO systems offer enhanced ease of

For peer review only - http://bmjopen.bmj.com/site/about/guidelines.xhtml

use and reduced burden, increased user satisfaction and lower rates of missing data for both patients and clinicians (15-18).

The evidence around implementation of ePROs in nephrology is growing. Studies from North America demonstrate the feasibility of ePRO collection in haemodialysis (HD) settings (16, 19, 20) and the Australian SWIFT (Symptom monitoring With Feedback Trial) study (21) and Canadian EMPATHY study (22) are cluster randomised controlled trials exploring the usefulness of integrating PRO assessments in the clinical management of patients receiving HD. In advanced CKD populations, the RePROM study in the UK piloted the use, and established feasibility, of an ePRO for remote symptom monitoring in real-time (9, 23) while the AmbuFlex telepro system is used in practice to manage renal follow up in Denmark (24, 25).

With this growth of ePRO system research and implementation in CKD globally, there is an opportunity to explore the key characteristics associated with successful implementation to support widespread adoption. Therefore, the objectives of this study were to (1) comprehensively identify ePRO systems for CKD, including those under development, (2) to explore system characteristics including methods of administration, levels of integration into existing workflow and electronic health records (EHR) and the reporting of ePROs to manage patient safety.

The overall aim being to provide a comprehensive classification of core factors which contribute to successful implementation of ePROs in nephrology, including common facilitators and barriers.

For peer review only - http://bmjopen.bmj.com/site/about/guidelines.xhtml

STUDY DESIGN

Methods

This was a three phased study which used mixed methods:

Phase 1 comprised a rapid scoping review identifying ePRO Systems and corresponding developers (26). Eligible systems were those used in nephrology practice (excluding acute kidney injury) including related treatment areas (e.g., dialysis units), that assessed PROs electronically and provided a summary of the patients' responses to their care provider. Eligible systems from any country were included, in routine use or under development/study. A system developer could have a clinical or non-clinical role but must have carried key responsibility for aspects of design, development, or implementation of an eligible ePRO system. Systems were identified through publications in English, including conference abstracts and grey literature, using 'pearl' and 'snowballing' methodology, i.e., searching from known key references and checking reference lists (27-29). Field experts were consulted. Data from these publications were used to create a structured survey exploring key system characteristics (See supplementary file 1).

Phase 2 involved survey administration via an online survey platform Smart Survey ™ (www.smartsurvey.co.uk) which was piloted prior to use. System developers were invited to participate via email. The survey focused on system design and software features, integration of e-PRO collection and reporting in clinical care.

Phase 3: Optional follow up semi-structured interviews were undertaken online via videoconferencing software with survey participants, or a suitable team member nominated by the original survey respondent. The purpose of these interviews was to expand on survey

For peer review only - http://bmjopen.bmj.com/site/about/guidelines.xhtml

responses and allow more detailed system/organisational enquiry. All follow up interviews used a piloted study-specific topic guide (see supplementary file 2)

Analysis

Data derived from the Phase 1 review were abstracted and charted, and descriptive statistics from the Phase 2 structured survey were tabulated. Analysis of Phase 3 interview data was undertaken using Codebook Thematic Analysis (30, 31) and the domains from the Consolidated Framework for Implementation Research (CFIR) (Figure 1). The CFIR is a determinant framework that can be used to identify and delineate contextual factors (i.e., barriers or facilitators) that influence the outcome of implementation efforts. (32, 33). Computer Assisted Qualitative Data Analysis Software CASDAQ (QSR NVIVO 12 (34)) was used to facilitate qualitative data analysis. Thematic analysis using a framework approach was chosen to systematically identify and analyse patterns of meaning within data, with the aim of highlighting the most salient features (30). The CFIR was used to gain insight into the overall effectiveness of the ePRO systems and associated implementation strategies. It is possible to rate constructs using the CFIR to undertake organisational comparison via Qualitative Comparative Analysis (QCA) (35). Due to the heterogeneity of systems, their context and stage of development, there was a danger of oversimplifying complex, dynamic descriptions of implementation processes and contexts so an aggregated data approach was not taken.

Patient and Public Involvement (PPI)

Patient partners were involved in the design of this study, and co-authored the final manuscript; where they specifically highlighted the importance of ensuring ePROs do not exacerbate existing health inequalities due to digital poverty (lack of digital access or skills)

For peer review only - http://bmjopen.bmj.com/site/about/guidelines.xhtml

and health data poverty: which has been defined as the inability for individuals, groups, or populations to benefit from a discovery or innovation due to insufficient data that are adequately representative (36).

RESULTS

The review identified 15 ePRO systems across seven countries: Canada n=4, United States of America n=3, United Kingdom n=4, Denmark n=1, France n=1, The Netherlands n=1, Australia n=1 (Table 1).

For peer review only

For peer review only - http://bmjopen.bmj.com/site/about/guidelines.xhtml

Table 1: Overview of ePRO systems

Name of System	Country	Renal Population	In current use Yes/No	System ePROs
**ANZDATA SWIFT PROMs Module (21, 37-39)**	Australia	In-centre HD	Yes – pilot study reported, Cluster RCT in progress	SONG-HD fatigue measure<br>IPOS-Renal<br>EQ-5D-5L
**cPRO-Collaborate Kidney Care (40)**	United States of America	CKD Stages 1-3<br>CKD Stages 4-5 pre dialysis	Yes – clinical demonstration model	PROMIS and FACIT measures to assess symptoms, social isolation, practical/psychosocial needs, nutritional needs, global health
**Cambian (ePRO-KIDNEY) (16, 41-44)**	Canada	Home HD, PD	No – platform used within reported research projects	Patient Assessment of Care for Chronic Conditions-20 questionnaire (PACIC-20)<br>Kidney Disease Quality of Life-36 (KDQOL-36)<br>Edmonton Symptom Assessment System renal (ESAS-r)<br>EQ-5D-5L
**OPT-ePRO (45-47)**	United Kingdom	CKD Stages 1-3, CKD Stages 4-5 pre dialysis<br>In-centre HD, Home HD, PD<br>Transplant, Conservative Care	No – pilot study reported	POS-S-RENAL, EQ-5D-5L
**Penguin (Cievert Ltd)<br>(no published data)**	United Kingdom	Transplant	Yes- Research pilot in progress	Penguin - New System specific measure (items unspecified)
**RePROM (9, 23, 48)**	United Kingdom	CKD Stages 4-5 pre dialysis	No – pilot study reported.	New study specific measure – items on fatigue; shortness of breath; loss of appetite; nausea or vomiting; itchiness or dry skin; pain; problems with fistula; faintness or dizziness; difficulty sleeping; restless legs; diarrhoea and ankle swelling. The questionnaire also includes an open text item
**Unnamed System (49)**	Canada	In-centre HD, Transplant	No – in development. Pilot study reporte	PROMIS CATs (unspecified)
**'Your symptoms matter' (50, 51)**	Canada	In-centre HD	Yes – pilot study reported	ESAS-r: Renal

For peer review only - http://bmjopen.bmj.com/site/about/guidelines.xhtml

***Survey data only - developers declined participation in optional FU interview**				
**Ambuflex (24, 52, 53)**	Denmark	CKD Stages 4-5 pre dialysis, Conservative Care	Yes – in routine care	Single items from KDQOL, EORTC and SF-36
**SMaRRT-HD (20, 54)**	United States of America	In-centre HD	Unknown – feasibility study reported	SMaRRT-HD
****System identified only – no survey or interview data included in evidence synthesis**				
**"Derby Evaluation of Illness" (55)**	United Kingdom	CKD stages 4/5 pre-dialysis, in-centre HD, PD	Unknown – feasibility study reported	Six separate domains assessed by VAS: general wellbeing, pain, sleep, breathing, energy, fistula function and appetite.
**EMPATHY study (22, 56-58)**	Canada	In-centre HD	Yes – Cluster RCT in progress	ESAS-r: Renal/IPOS-Renal and/or the EQ-5D-5L PROMs are administered by pen and paper or through an electronic platform on a tablet in accordance with HD unit resources.
**K-Pal (19)**	United States of America	Patient's ≥60 years of age with ESRD on HD	Unknown – feasibility study reported	Short-Form McGill Pain Questionnaire 2 (SF-MPQ-2), Patient Health Questionnaire-9 (PHQ-9), Generalized Anxiety Disorder 7 Item Survey (GAD-7), Dialysis Symptom Index (DSI), Kidney Disease Quality of Life (KDQOL-36)
**Unnamed system (59)**	France	HD patients in a dialysis unit	Unknown – ePRO system used to describe symptom prevalence – unclear if responses feedback to care provider.	New study specific measures (items on stress/fatigue/sleep disturbance and overall health status)
**Dutch Renal Registry (60-62)**	The Netherlands	Advanced CKD and ESKD with and without renal replacement therapy	Yes – part of development of national registry of PROMs included in Dutch Renal Registry	Dialysis Symptom Index (DSI), SF-12

For peer review only - http://bmjopen.bmj.com/site/about/guidelines.xhtml

Ten system developers responded to the survey request (66% response rate). System developers held varying academic and clinical academic research posts, all demonstrating outcomes methodology expertise. Key responsibilities ranged from funding acquisition and stakeholder engagement, to overseeing system development, piloting, and evaluation. Seven developers agreed to a follow up semi-structured interview: one expert delegated this task to a member of their study team.

Key system characteristics

Table 2 outlines characteristics of surveyed systems and system objectives.

For peer review only

Table 2: System Characteristics

System Characteristic/Feature<br>*multiple response options total ≠ 100%	Number<br>n=10<br>(Skipped response)	Response %
System launched	(0)	
In development	1	10%
<1 year	1	10%
1-5 years	5	50%
>5 years	3	30%
Primary Objective of System*	(0)	
Improving symptom assoc with CKD	7	70%
Improving symptom assoc with treatment	5	50%
Psychosocial care	0	0%
Facilitate communication	4	40%
Research	3	30%
Benchmarking	0	0%
Commissioning	0	0%
Support transition of care	0	0%
Secondary Objective of System*	(0)	
Improving symptom assoc CKD	2	20%
Improving symptom assoc treatment	4	40%
Psychosocial care	8	80%
Facilitate communication	6	60%
Research	4	40%
Benchmarking	2	20%
Commissioning	1	10%
Support transition of care	2	20%
How developed?	(0)	
In house informatics team	5	50%
Collaboration across sectors	4	40%
Commercial product	1	10%
Funding Source*	(0)	
Commercial	1	10.0%
Academic	4	40.0%
Charitable	3	30.0%
Government	3	30.0%
No of patient users	(1)	
In development	1	11.1%
<100	3	33.3%
100-500	3	33.3%
>500	2	22.2%
Primary location of use*	(0)	
Primary Care (Community/General Practice clinic)	10.0%	1
Secondary Care (Hospital clinic)	80.0%	8
Dialysis Centre	60.0%	6
Home	20.0%	2

For peer review only - http://bmjopen.bmj.com/site/about/guidelines.xhtml

**System platform***	(0)	
**Non-responsive website**	2	20.0%
**Responsive/mobile website**	8	80.0%
**Mobile application**	2	20.0%
**System Access***	(0)	
**Computer**	7	70.0%
**Tablet**	10	100.0%
**Interactive Voice Response System**	0	0.0%
**Clinic Based kiosk**	2	20.0%
**Smartphone**	7	70.0%
**PRO selection***	(2)	
**Automatic (by system) i.e., Provider**	8	100%
**By Patient**	0	0.0%
**System security features***	(1)	
**Secure Log in**	9	100.0
**Encryption**	7	77.8%
**two factor log in**	1	11.1%
**Unsure**	1	11.1%
**Page features***	(1)	
**Progress Bar**	6	66.7%
**Visual graphics i.e., graph, diagram, chart**	4	44.4%
**Automatic save function, Option to save and return later**	1	11.1%
**Training available**	(0)	
**Yes**	9	90%
**No**	1	10%
**Who receives training? ***	(1)	
**Clinical staff**	9	100.0%
**Administrative staff**	4	44.4%
**Patients**	7	77.8%
**Carers**	0	0.0%
**Form of e PRO system training***	(1)	
**Face to face**	7	77.8%
**Online**	7	77.8%
**Integrated into ePRO system**	2	22.2%
**Facility for patient education***	(2)	
**Administered in system**	4	50.0%
**Education linked to ePRO scores**	1	12.5%
**Automatic documentation of action**	0	0.0%
**No patient education offered**	4	50.0%
**In development**	1	12.5%
**Has the system been evaluated?**	(0)	
**Yes**	7	70%
**No**	3	30%

For peer review only - http://bmjopen.bmj.com/site/about/guidelines.xhtml

The most common primary objective was symptom management associated with CKD and/or renal replacement therapy by HD. However, systems were implemented across kidney disease stages and treatment modalities (see Table 1). A key secondary goal was the identification of psychosocial problems. Aggregate uses of ePROs for benchmarking or commissioning of services were not common; one system collected satisfaction with care data (63), and two others assessed quality of care (21, 40). There was variation in funding source; academic (40%), government (30%) and charitable funding (30%), with 70% of systems being developed in the last five years. Half of the systems were developed in-house by local informatics teams, with the rest reporting collaborations across sectors with registries and technology companies. Systems allowed ePROs to be completed in different settings; the most common setting for completion was hospital clinic (80%). Only one system could be accessed by primary care (40).

One surveyed system had been implemented into routine practice, Ambuflex (25); all other systems were at varying stages of development with most having conducted pilot/feasibility testing. Two identified systems were being evaluated within a cluster randomised controlled trial (21, 22); while another was being evaluated in a co-designed demonstration trial (40) (see Table 1). Tablet was the most popular mode of administration. Two systems offered a clinic-based kiosk facility (25, 50). Training was available or planned for clinical staff in all systems but focused largely on system access and functionality. Seven systems had developed training materials for patients and four for administrative staff. Four systems had patient education material administered within the system, only one of these linked self-management guidelines to scores (9). Systems were evaluated by various means including formative research (57.1%), progress-focused evaluation concurrent to implementation

For peer review only - http://bmjopen.bmj.com/site/about/guidelines.xhtml

(57.1%) and retrospective evaluation (14.3%). Patients were involved in evaluation strategies, influencing changes to content/order of measures, timing and frequency of administration, improved data visualisation to show trends and the addition of symptom guidance. Only 60% of systems were currently linked with the EHR.

Data collection and assessment

Table 3 summarises key data collection functions.

Table 3: Data collection and assessment

Data Collection/Assessment<br>*multiple response options total ≠ 100%	Number n=10<br>(Skipped responses)	Response%
**Measure development***	(0)	
System uses new PROM	5	50%
System uses existing PROM	7	70%
**Timing of Assessment***	(0)	
Prior to clinical assessment	4	40.0%
Set time point weekly	0	0.0%
Set time point monthly	1	10.0%
Set time point 3 monthly	3	30.0%
Set time point 6 monthly	1	10.0%
Set time point annually	0	0.0%
As required (by patient)	2	20.0%
Other	4	40.0%
**Question format**	(1)	
One question per page	3	33.3%
Multiple questions per page	0	0.0%
Mixed format of single and multiple questions	6	66.7%
**Question Advancement**	(1)	
Mouse click	6	66.7%
Automatic on completion	3	33.3%
**Analysis Metric***	(0)	
Change from baseline	7	70.0%
Final value	6	60.0%
Time to event	0	0.0%
Other (system defined)	2	20.0%

For peer review only - http://bmjopen.bmj.com/site/about/guidelines.xhtml

**Avoidance of Missing data***	(1)	
**Allows multiple logins per assessment with automatic save function**	0	0.0%
**Allows multiple logins per assessment with save and return later function**	3	33.3%
**Allows not applicable (N/A) response**	2	22.2%
**Default response pre-selected (pre-populated neutral response)**	0	0.0%
**Reminders**	5	55.6%
**Other: i.e., Mandatory fields**	4	44.4%
**Notification of completed assessments***	(0)	
**Automated submission notification to patient**	1	10.0%
**Automated submission notification to clinicians**	4	40.0%
**Email notification to patient from clinical team following review of responses**	2	20.0%
**App based notification**	0	0.0%
**Other: None/unsure**	3	30.0%
**Patient Reminder format***	(0)	
**Email**	5	50.0%
**Telephone call**	2	20.0%
**Text message: SMS**	2	20.0%
**Verbally**	5	50.0%
**By letter**	3	30.0%
**No reminder**	0	0.0%
**App based push notification**	1	10.0%
**Flexible system features***	(1)	
**Web based home and clinic login access**	7	77.8%
**Multiple assessment scheduling options**	3	33.3%
**Two or more sources for PRO selection (patient/provider)**	1	11.1%
**Self-identification of important issues by patient (CAT functionality)**	3	33.3%
**Free text availability**	8	88.9%
**Multiple language availability**	3	33.3%
**In app or push notifications**	0	0.0%
**Other: facility for multiple Lang/CAT currently not used**	1	11.1%

Half the developers reported using a newly developed PRO measure, either completely new or a mix of existing tools. Only three systems had ePRO availability in languages other than English (20, 21, 50, 54), although several developers reported plans for future translations, dependent on funding. There were a mixture of mechanisms to remind patients to complete

For peer review only - http://bmjopen.bmj.com/site/about/guidelines.xhtml

their ePROs, with email (50%) and verbal reminders (50%) being most popular. Reminders were seen as the best way to avoid missing data (55.6%), but some systems offered a save and return function.

Frequency of reporting was varied, with most common timing of assessment being prior to a clinical assessment (40%). However, some systems did have set time points ranging from one to six monthly. Two systems allowed patients to complete as required (9, 50).

System reporting

Table 4 outlines system reporting features.

Table 4: System reporting

System reporting/Integration<br>*multiple response options total ≠ 100%	Number<br>n=10<br>(Skipped responses)	% Response
ePRO report access*	(0)	
Via clinical portal/electronic patient health record	7	70.0%
Via immediate access i.e., summary print out on completion	4	40.0%
Results restricted to clinical encounter	0	0.0%
Other: Registry, emailed PDF to patient	2	20.0%
ePRO report content*	(0)	
Current scores including summary and individual scores	7	70.0%
Longitudinal change	8	80.0%
Interpretation included in report	3	30.0%
Cut scores (e.g., low, medium, high)	4	40.0%
Population norms or reference values	2	20.0%
Identification of meaningful change	1	10.0%
Modifiable reports	1	10.0%
General guidelines	4	40.0%
Other i.e., colour coding	3	30.0%
Who has access to report/summary*	(0)	
Patient	10	100.0%
Clinicians	10	100.0%

For peer review only - http://bmjopen.bmj.com/site/about/guidelines.xhtml

Care Provider	3	30.0%
Multiple care provider access	3	30.0%
Researcher	1	10.0%
**Who is responsible for initial review & action**	(1)	
Medical staff	4	44.4%
Nursing staff	5	55.6%
Administrative staff	0	0.0%
Other member of multidisciplinary team	0	0.0%
**Form of clinical response***	(0)	
Prescribed electronic response dependent on score	2	20.0%
Clinician/staff follow up; follow up type (face to face, virtual) dependent on score/decision aids	9	90.0%
Automatic referral to member of multidisciplinary tea	1	10.0%
Automated patient education/message regardless of score	1	10.0%
Self-management support resource dependent on score	1	10.0%
**Visual presentation of PROs scores***	(0)	
Graphical	8	80.0%
Tabular	3	30.0%
Numerical	3	30.0%
Emoticon	1	10.0%
Colour coded	5	50.0%
Other: No score/being developed for longitudinal data	2	20.0%
**Safety Alert System (eAlert) – dependent on PRO score**	(0)	
Yes		
No	3	30.0%
	7	70.0%
**Intended recipient for eAlert**	(7)	
Clinician/Staff	3	100%
Patients	0	0.0%
Care provider	0	0.0%
Caregiver	0	0.0%
Multiple recipients i.e., clinicians and patients	0	0.0%
**Form of eAlert***	(7)	
Email	2	66.7%
Text message/SMS	0	0.0%
Telephone call	0	0.0%
Verbal	0	0.0%
Real time alert	2	66.7%
In App notification	0	0.0%
None	0	0.0%

For peer review only - http://bmjopen.bmj.com/site/about/guidelines.xhtml

All systems allowed patients and clinicians direct access to ePRO results or summary reports. Doctors (44.4%) and nurses (55.6%) were responsible for initial review and action of results. Follow-up (face to face, virtual, telephone) was largely dependent on scores. Electronic PRO reports contained current scores (summary and individual scores) in 70% of systems, 80% presented longitudinal changes and 40% offered cut scores (low, medium, high). One system was part of an overall dashboard with the option for patients to combine ePRO scores with lab values in a graph (40). In terms of data visualisation, graphical presentation was most popular (80%) with 50% of ePROs using colour coding to highlight significance of scores. Less frequently used approaches of score presentation were tables (30%), numerical scores (30%) and emoticons (10%). Assistance with interpretation within the report was uncommon, but 30% of ePROs had developed a safety alert system dependent on respondent scores (9, 20, 25). Two of these systems could produce real-time alerts directly to clinicians (9, 20), while the AmbuFlex system keeps track by giving patients a red and yellow and non-responder status, which are presented to clinicians on an alert list (25).

Follow up interviews

Semi-structured interviews were carried out with seven ePROs developers, which allowed clarification of survey responses and explored the development process, to identify contextual factors working for and against implementation. Analysis of the interviews generated rich data which are presented in line with the CFIR key domains (32). Figure 1 outlines the conceptual framework (Illustrative quotations to support the key findings are provided in supplementary file 3). Although presented in a linear fashion, the five domains and their respective constructs together provide context for implementation.

For peer review only - http://bmjopen.bmj.com/site/about/guidelines.xhtml

Intervention Characteristics: Factors associated with design and quality of the intervention, including how intervention is perceived.

Analysis highlighted the heterogeneity of the systems. However, developers agreed that systems need to be flexible, responsive, and follow an iterative development strategy that involves a process of refinement and evaluation using stakeholder feedback. All interview participants felt that their systems would need future refinement, whether simple adaption to increase language availability or more complex programming to allow real-time symptom alerting or provision of self-management tools. Knowledge of context (population and setting) and purpose of ePRO data collection should be clearly defined and communicated to all stakeholders including patients. Developers acknowledged that the evidence base of the benefit of ePROs is less mature in nephrology than other clinical specialities. However, participants indicated ePROs could deliver a relative advantage over current care delivery: improving communication, supporting symptom management, enhancing self-care and shared decision making, with potential to reduce healthcare utilisation. However, all participants recognised the challenges, particularly of clinician engagement. They cited clinician anxieties around potential increases in workload and attendance to symptoms and problems unrelated to kidney disease. Consequently, several developers chose not to include real-time safety alerts associated with severe symptom reports.

Several systems used currently available infrastructure, utilising existing platforms, registries, and validated measures. Whilst this offered the advantage of existing expertise and reduced cost, this was offset by restrictions on local adaption and global comparability due to lack of harmonisation.

For peer review only - http://bmjopen.bmj.com/site/about/guidelines.xhtml

Outer Setting: the wider societal, economic, and organisational context

Developers all reported that their ePROs system programs had been impacted and delayed by the COVID-19 pandemic. Several developers reported that key staff including informatics teams reprioritised activity and had less availability to support ePRO implementation. However, they stated that the rapid shift to virtual consultations and the recognition of the potential impact of ePROs in managing care and particularly outpatient planning invigorated interest in ePROs.

Developers highlighted the importance of partnership and collaboration – across clinical, academic, and commercial organisations. Registries were seen as an ideal collection platform. However, developers indicated multi-organisational approaches could lead to competing interests and governance issues that can cause delays.

Developers acknowledged the value of integration of ePROs directly into the EHR, but not all systems had this functionality. Some still required manual entry, which was reported as time consuming, costly and would not support real-time care. Many systems were standalone in nephrology secondary care, meaning data would not 'follow' patients across care settings and therefore could not be used to manage transition of care needs.

Inner Setting: the structural and cultural contexts around where the implementation will take place

Developers suggested the potential unintended consequences of ePRO implementation included further widening of health inequities, due to inadequate accessibility to digital devices, variable digital competence, and lack of inclusivity caused by poor availability of

For peer review only - http://bmjopen.bmj.com/site/about/guidelines.xhtml

systems in multiple languages or failure to assess cultural compatibility. Some developers were investigating such outcomes in particularly vulnerable groups, e.g., elderly, ethnic minorities.

Some developers cited the continued prevalence of biomedical models of care, despite general acceptance of the need to move to person-centred care, as a barrier to ePRO implementation. They indicated approaches for shifting 'power' to patients by focusing on patient understanding of ePROs and involvement at all stages of design and implementation, along with patient peer support to counteract cultural barriers.

Doctors were the professional group most often involved in review and action of ePRO responses, but several systems directed ePRO scores to nursing staff or physician assistants as the initial handler. Resources needed to be available to allow the engagement of the wider multi-disciplinary team including supportive and rehabilitation services.

While clinicians were largely receptive to ePROs, workflow compatibility was seen as crucial to uptake, and this was reflected in the preference for a focus on symptom management as primary objective of capture. Several systems included free text options to report symptoms or problems and clinicians needed reassurance that this would not significantly impact workload. Participants reported that pilot study data suggested free text responses did not adversely increase workload but provided another mechanism for patient/provider communication, likewise with initial data on use of symptom report alerts.

For peer review only - http://bmjopen.bmj.com/site/about/guidelines.xhtml

Characteristics of Individuals: Factors associated with individuals involved in implementation

Participants discussed the importance of measuring outcomes which matter most to patients but emphasized the hypothetical danger of over digitising the patient experience, meaning that clinicians risk responding to scores alone rather than listening and connecting with the patient to further interpret ePRO data. Several participants looked forward to a future when ePROs might be given similar status to other forms of medical data. They noted clinicians frequently discuss burden associated with the collection and use of ePROs but did not consider similar factors associated with non-essential blood or invasive tests.

Developers reflected on the varied ability to deal effectively with responses, both at an organisational and individual level. They recognised the heavy and complex workload within the speciality, and while some systems offered management guides or further resources, participants were not all in favour of prescriptive treatment decision algorithms, some preferring to allow the exercise of clinical judgement.

The Process of Implementation

Systems were in different stages of planning, development, and feasibility to assess implementation and some were part of wider programs of research. A pragmatic approach to implementation was often described: for example, measures were chosen according to what was already being used and what patients would be prepared to complete. Existing platforms that had been previously assessed for governance and regulatory compliance were exploited. Implementation readiness for use in routine care was affected by most systems being investigated outside a real-world context. Sustainability was an issue: future

For peer review only - http://bmjopen.bmj.com/site/about/guidelines.xhtml

planning would need to include activities undertaken by research personnel including administration tasks, completion support and training. Long term IT and informatics support needs to be factored into costing for maintenance and adaption.

All developers stated the importance of patient involvement at all stages and outlined different ways patients had offered input and insight from co-design/production to research participation in usability testing. Staff dedicated to engagement were useful, ensuring extended community groups were involved.

Training associated with the education and knowledge needed to support ePROs appeared to be under-developed when compared to other aspects of system design. Several developers stated that training resources were in development and current iterations focused predominantly on how you use the system and did not extend to interpreting or actioning responses. It was recognised that training was time consuming and needed to be ongoing.

Overarching themes: Barriers and Facilitators to effective implementation

Across all CFIR constructs, analysis identified potential barriers and facilitators. Key barriers included the culture associated with biomedical models, variable clinical engagement particularly from doctors, and governance issues. Participants suggested facilitators included the development of patient driven systems, utilisation of clinical champions who need both time and belief to promote the system, and developing existing resources where adaption was possible.

For peer review only - http://bmjopen.bmj.com/site/about/guidelines.xhtml

DISCUSSION

This review summarises available data on the features and implementation facilitators and barriers of 15 nephrology specific ePRO systems used across seven countries.

The majority of surveyed systems prioritised symptom management associated with underlying CKD (70%) or treatment (50%), over other potential uses of ePROs such as benchmarking and commissioning (see table 2). While some systems were designed to collect ePROs across stages of kidney disease and treatment modalities, patients undergoing HD were the most common population for ePROs use. According to recent reviews, patients with CKD report between 56-68 common and/or severe signs and symptoms, with the exact symptom burden often determined by stage of disease and treatment (3, 64). Participants in this study reflected that management of symptoms was a good primary objective on which to concentrate early implementation efforts, with capability to add additional functionality and use in the future.

Stakeholder involvement

Our study has highlighted the increased development of nephrology ePROs in the last five years, with all developers reporting positive and negative effects of the COVID-19 pandemic. While there were delays to implementation due to reprioritisation of activity, the increased drive to deliver patient centred care via telemedicine has enhanced attention on ePROs.

To deliver person-centred care it is necessary to focus on what matters to patients, and correspondingly patients must be involved in determining which health outcomes to measure (65). All the surveyed systems had involved patients in aspects of development,

For peer review only - http://bmjopen.bmj.com/site/about/guidelines.xhtml

including evaluation. Patient and public involvement (PPI) is a prerequisite to inclusive and equitable PROs research (66) and the meaningful involvement of patients in ePROs implementation programs is crucial to effective uptake (67). Well-established PPI groups, reflective of the total population, can offer advice on measure choice, language requirements and support resources. Patient input in design of software features including reporting and data visualization can ensure data is interpreted correctly (68) using various devices such as graphs, emoticons and heat maps (69, 70). Patient 'navigators' can offer health literacy and communication peer support for successful digital completion (71).

While important contributors to ePROs implementation are quality assurance, governance, informatics, and information technology departments, any successful ePROs system will need effective clinician 'buy in'. This study identified this as a key challenge, particularly from those clinicians not involved in ePROs research. Clinical staff state they are anxious about disruptions to already tight workflows and while ePROs are seen as a useful conversation starter, clinicians fear ePROs lack clinical utility for decision making, citing the current lack of empirical evidence on the benefits of using this data in nephrology (72, 73). There is a need for research to improve the evidence-base of ePRO use at individual and aggregated levels of kidney care (74), including studies which employ cluster designs and utilise techniques to maintain allocation concealment (75). Studies included in this review are going some way to provide this evidence.

Developers described utilising the assistance of 'clinician champions'; individuals with both the time and influence to drive ePRO implementation. These champions would encourage

For peer review only - http://bmjopen.bmj.com/site/about/guidelines.xhtml

clinicians to view ePRO scores as key health data, in the same way as a biomarker or a lab test, to manage care.

Studies have highlighted tension from clinicians in their ability to respond to ePROs, whether due to workload or belief that their scope of practice should be confined to matters related to nephrology (39, 73, 76). Some clinicians indicate they are more comfortable assessing, rather than actively managing, psychosocial symptoms (50). While holistic care is advocated within kidney care, it is not necessarily supported within the system and multidisciplinary approaches and further training in managing mental health problems is needed (56). Although research from other specialities suggests patient encounters using ePROs do not take longer (77), studies on workflow changes and impact are required.

Real-world implementation

A shift to implementation science for guidance on how to promote adoption, enhance readiness and optimise use of ePROs in real-world settings will also identify any unintended consequences of use. These include exacerbation of health inequalities and potential over digitisation of the patient experience in an already highly technical speciality (78, 79).

Many groups who are already subject to disadvantage and worse health outcomes are also subject to digital exclusion (80). Flexible systems enhance accessibility, offering patients' choice on time, frequency, mode of administration and place of completion. Electronic PROs are commonly completed in a healthcare facility via tablet, where completion support is available. However, onsite completion may impact workflow and support assistance traditionally delivered by research staff will need to be factored into sustainability plans. Home completion may need to be incentivised and alternative means of completion should

For peer review only - http://bmjopen.bmj.com/site/about/guidelines.xhtml

be offered as required i.e., voice activation response systems, hardcopy by post; following assessment of local population needs. Combinations of digital inclusion approaches are likely needed, to support patients with the digital and health literacy skills required to negotiate both their electronic health records (EHR) and any corresponding ePRO data (81) and offer access to hardware (66, 80). Evaluation of current ePROs systems should investigate whether ePROs are excluding already disadvantaged groups or if these groups might benefit through access to remote follow up; perhaps saving money from reduced transport and time off work costs.

While most systems offered or were developing self-management guidance for patients, a minority offered guidelines for health professionals in the form of treatment algorithms or decision tools based on patient scores. Effective training needs analysis at the planning stage is crucial to assess the support needed by the multidisciplinary team to respond to ePROs. Generally, systems were being developed iteratively, with training and support materials being developed or expanded after launch and this may be counterproductive to early and sustained engagement from clinical teams.

A pragmatic approach was adopted by developers, recognising the need for flexible, iterative systems capable of capturing and giving responses in real time, to meet needs of local population. Three systems had chosen to collect data via an existing national renal registry (21, 60, 82) and while this existing infrastructure supports secure collection, transfer and display of ePRO data with advantages for scaling up, this has to be offset against overall registry priorities and functionality; i.e., it may not be possible to offer real-time responses

For peer review only - http://bmjopen.bmj.com/site/about/guidelines.xhtml

without local adaptions. Box 1 outlines key priorities for successful implementation.

Box 1: Key priorities for successful implementation

Macro level priorities (National) – formation of national collaborative groups to promote equitable and sustainable uptake of ePROs systems that have multiple application	Harmonisation of collection platforms e.g., National Registry – allowing data linkage to other healthcare settings i.e. primary care<br>Harmonisation of measures (including item banks)<br>Harmonisation of methods of interpretation and analysis metrics<br>High quality research to demonstrate empirical evidence of benefit including real world evaluation<br>Continued research on psychometric properties and interpretation of measures<br>Investigation of new technologies i.e. CAT<br>Endorsement of key guidelines such as the PCORI Users' Guide to Integrating Patient-Reported Outcomes in Electronic Health Records (83), ISOQOL Implementing PROs in clinical practice (84) and the ISPOR Validation of Electronic Systems to Collect Patient-Reported Outcome (PRO) Data Recommendations (85).
Meso level priorities (organisational) - key contributors to include clinicians, patients, carers, IT, informatics, QA, governance departments - Local systems specificity to maximise implementation and minimise effects on workflow	Involve all stakeholders – define and communicate key system objectives<br>Use flexible system design to facilitate local, regional and national compatability<br>EHR compatability<br>Allow varying modes of administration, including paper<br>Reminders – varying forms (email, app alert etc.)<br>Alert systems – develop pathways and algorithms for action that compliment workflow<br>High levels of automation to reduce workload<br>Data collection features – need efficient, easy user experience<br>Easy to access, save, submit and review<br>Optimise data interpretation and visualisation<br>Infrastructure support to deliver 'holistic care' – treatment and care algorithms<br>Appointment and support for clinical 'champions'<br>Patient peer support via 'Patient Navigators'<br>Training packages to support implementations for all users<br>Sustained IT support<br>Ongoing evaluation programmes
Micro level priorities (Individual)<br>Involve all key stakeholders and undertake meaningful PPI activity to ensure ePROs are accessible and inclusive for population	Identify local needs i.e., language availability requirements<br>Provide support for those with poor health or digital literacy (need an assessment of needs)<br>Optimise accessibility – allow onsite completion via kiosk or tablet, enable 'bring your own device'<br>Evalution by patients to ensure systems are not over-digitising the patient experience by not discussing responses with individuals.

For peer review only - http://bmjopen.bmj.com/site/about/guidelines.xhtml

Areas for future research

Data linkage is central to multiple usages and harmonisation of collection platforms and associated measures will support this. Integration of ePROs within the EHR can offer ‘one stop’ access for clinicians and patients, if given access. Extended accessibility across healthcare providers would allow long-term monitoring across the kidney care continuum; none of the systems currently have this level of functionality.

New technologies, such as computerized adaptive tests (CATs) offer sophisticated measure delivery using algorithms based on item response theory to personalize ePROs for patients. CAT based measures are shorter, more accurate, and efficient (86). None of the reviewed systems were currently using CAT administration, however, several developers reported this was where they saw the future of ePROs and were actively researching this area; a renal specific item bank and associated CAT is currently being developed for use in the UK (87).

There has been considerable research investment in the development of ePROs to measure CKD symptoms and HRQOL. The next step is to accelerate the implementation gap between research and practice; this study supports this objective by outlining key system features and exploring factors to optimise the delivery ePROs in routine care settings within nephrology.

Word count= 4249

Ethical Approval:

Ethics approval was issued on 06/07/2021 by University of Birmingham Science, Technology, Engineering and Mathematics Ethical Review Committee: ERN_21-0495

For peer review only - http://bmjopen.bmj.com/site/about/guidelines.xhtml

Contributorship statement:

NA, MC, CM, PC, OLA, DK: Substantial contribution to the conception and design of the study

NA: Data collection

NA, CM Analysis and interpretation of data for the work

NA, CM, DK, PC, OLA, MC, RV: Drafting and revising the work critically for important intellectual content and final approval of version to be published and agreement to be accountable for all aspects of the work in ensuring that questions related to the accuracy or integrity of any part of the work are appropriately investigated and resolved.

Competing interests:

NEA receives funding from the National Institute of Health and Care Research (NIHR) under its Clinical Doctoral Research Fellowship Programme (Grant Reference ICA-CDRF-2018-04-ST2-027) and declares personal fees from GlaxoSmithKline (GSK) outside the submitted work.

MC is Director of the Birmingham Health Partners Centre for Regulatory Science and Innovation, Director of the Centre for the Centre for Patient Reported Outcomes Research and is a National Institute for Health and Care Research (NIHR) Senior Investigator. MC receives funding from the NIHR Birmingham Biomedical Research Centre, NIHR Surgical Reconstruction and Microbiology Research Centre, NIHR Birmingham-Oxford Blood and Transplant Research Unit (BTRU) in Precision Transplant and Cellular Therapeutics, and NIHR ARC West Midlands at the University of Birmingham and University Hospitals Birmingham NHS Foundation Trust, Health Data Research UK, Innovate UK (part of UK Research and Innovation), Macmillan Cancer Support, SPINE UK,UKRI, UCB Pharma,

For peer review only - http://bmjopen.bmj.com/site/about/guidelines.xhtml

Janssen, GSK and Gilead. MC has received personal fees from Astellas, Aparito Ltd, CIS Oncology, Takeda, Merck, Daiichi Sankyo, Glaukos, GSK and the Patient-Centered Outcomes Research Institute (PCORI) outside the submitted work.

DK reports grants from Macmillan Cancer Support, Innovate UK, the NIHR, NIHR Birmingham Biomedical Research Centre, and NIHR SRMRC at the University of Birmingham and University Hospitals Birmingham NHS Foundation Trust, and personal fees from Merck and GSK outside the submitted work. DK has received funding from the NIHR and Kidney Research UK and is Chief Investigator for the RePROM and RCAT studies. CM receives funding from the National Institute for Health Research (NIHR) Surgical Reconstruction and Microbiology Research Centre, the NIHR Birmingham-Oxford Blood and Transplant Research Unit (BTRU) in Precision Transplant and Cellular Therapeutics, Innovate UK, and has received personal fees from Aparito Ltd outside the submitted work. OLA receives funding from the NIHR Birmingham Biomedical Research Centre (BRC), NIHR Applied Research Collaboration (ARC), West Midlands, NIHR Birmingham-Oxford Blood and Transplant Research Unit (BTRU) in Precision Transplant and Cellular Therapeutics at the University of Birmingham and University Hospitals Birmingham NHS Foundation, Innovate UK (part of UK Research and Innovation), Gilead Sciences Ltd, Janssen pharmaceuticals, Inc, and Sarcoma UK. OLA declares personal fees from Gilead Sciences Ltd, GlaxoSmithKline (GSK) and Merck outside the submitted work.

The views expressed are those of the author(s) and not necessarily those of the NHS, the NIHR or the Department of Health and Social Care. The study sponsor and funders have no

For peer review only

For peer review only - http://bmjopen.bmj.com/site/about/guidelines.xhtml

role in study design, including collection, management, analysis, and interpretation of data; writing of the report and the decision to submit the report for publication.

Funding:

This manuscript refers to independent research funded by the National Institute for Health and Care Research (NIHR) under its Clinical Doctoral Research Fellowship Programme (Grant Reference ICA-CDRF-2018-04-ST2-027)

Data sharing statement:

De identified data may be available upon reasonable request via the corresponding author (ORCID 0000-0002-0614-3198).

For peer review only

For peer review only - http://bmjopen.bmj.com/site/about/guidelines.xhtml

References

1. Almutary H, Bonner, A, Douglas, C. Symptom burden in Chronic Kidney Disease: a review of recent literature. Journal of Renal Care. 2013;39(3):140-50.

2. Lowney AC, Myles HT, Bristowe K, Lowney EL, Shepherd K, Murphy M, et al. Understanding What Influences the Health-Related Quality of Life of Hemodialysis Patients: A Collaborative Study in England and Ireland. J Pain Symptom Manage. 2015;50(6):778-85.

3. Fletcher BR, Damery S, Aiyegbusi OL, Anderson N, Calvert M, Cockwell P, et al. Symptom burden and health-related quality of life in chronic kidney disease: A global systematic review and meta-analysis. PLoS Med. 2022;19(4):e1003954.

4. Anderson NE, Calvert M, Cockwell P, Dutton M, Kyte D. The Use of Patient-Reported Outcomes in Patients Treated With Maintenance Hemodialysis: A Perspective. Am J Kidney Dis. 2019;74(3):399-406.

5. Tang E, Bansal A, Novak M, Mucsi I. Patient-Reported Outcomes in Patients with Chronic Kidney Disease and Kidney Transplant-Part 1. Front Med (Lausanne). 2017;4:254.

6. United States of America Department of Health and Human Services Food and Drug Administration. Guidance for industry. Patient reported outcomes measaures: Use in medical product development to support labeling claims.; 2009 Dec 2009. Report No.: http://www.fda.gov/Drugs/GuidanceComplianceRegulatoryInformation/Guidances/default.htm.

7. Hull SA, Rajabzadeh V, Thomas N, Hoong S, Dreyer G, Rainey H, et al. Do virtual renal clinics improve access to kidney care? A preliminary impact evaluation of a virtual clinic in East London. BMC Nephrol. 2020;21(1):10.

8. Young A, Orchanian-Cheff A, Chan CT, Wald R, Ong SW. Video-Based Telemedicine for Kidney Disease Care. Clinical Journal of the American Society of Nephrology. 2021;16(12):1813-23.

9. Kyte D, Anderson N, Auti R, Aiyegbusi OL, Bishop J, Bissell A, et al. Development of an electronic patient-reported outcome measure (ePROM) system to aid the management of patients with advanced chronic kidney disease. J Patient Rep Outcomes. 2020;4(1):55.

10. Lizée TB, Ethan ; Trémolières, Pierre ; Voog, Eric ; Domont, Julien ; Peyraga, Guillaume ; Urban, Thierry ; Bennouna, Jaafar ; Septans, Anne-Lise ; Balavoine, Magali ; Detournay, Bruno ; Denis, Fabrice. Cost-Effectiveness of Web-Based Patient-Reported Outcome Surveillance in Patients With Lung Cancer. Journal of Thoracic Oncology. 2019;14(6):1012-21.

11. Velikova G, Booth L, Smith AB, Brown PM, Lynch P, Brown JM, et al. Measuring quality of life in routine oncology practice improves communication and patient well-being: a randomized controlled trial. J Clin Oncol. 2004;22(4):714-24.

12. McCann L, Maguire R, Miller M, Kearney N. Patients' perceptions and experiences of using a mobile phone-based advanced symptom management system (ASyMS) to monitor and manage chemotherapy related toxicity. Eur J Cancer Care (Engl). 2009;18(2):156-64.

13. Basch E, Deal AM, Dueck AC, Scher HI, Kris MG, Hudis C, et al. Overall Survival Results of a Trial Assessing Patient-Reported Outcomes for Symptom Monitoring During Routine Cancer Treatment. JAMA. 2017;318(2):197-8.

14. Basch E, Stover , A, M, Schrag, D, Chung, M, Jansen, J, et al. Clinical Utility and User Perceptions of a Digital System for Electronic Patient-Reported Symptom Monitoring During

For peer review only - http://bmjopen.bmj.com/site/about/guidelines.xhtml

Routine Cancer Care: Findings From the PRO-TECT Trial. JCO Clinical Cancer Informatics. 2020;4:947-57.
15. Meirte J, Hellemans N, Anthonissen M, Denteneer L, Maertens K, Moortgat P, et al. Benefits and Disadvantages of Electronic Patient-reported Outcome Measures: Systematic Review. JMIR Perioper Med. 2020;3(1):e15588.
16. Schick-Makaroff K, Molzahn A. Brief Communication: patient satisfaction wit the use of tablet computers: a pilot study in two outpatient home dialysis clinics. Canadian Journal of Kidney Health and Disease. 2014;1(22).
17. Schick-Makaroff K, Molzahn AE. Evaluation of real-time use of electronic patient-reported outcome data by nurses with patients in home dialysis clinics. BMC Health Serv Res. 2017;17(1):439.
18. Aiyegbusi OL, Nair D, Peipert JD, Schick-Makaroff K, Mucsi I. A narrative review of current evidence supporting the implementation of electronic patient-reported outcome measures in the management of chronic diseases. Therapeutic Advances in Chronic Disease. 2021;12.
19. Gabbard J, McLouth CJ, Brenes G, Claudel S, Ongchuan S, Burkart J, et al. Rapid Electronic Capturing of Patient-Reported Outcome Measures in Older Adults With End-Stage Renal Disease: A Feasibility Study. Am J Hosp Palliat Care. 2021;38(5):432-40.
20. Flythe JE, Tugman MJ, Narendra JH, Dorough A, Hilbert J, Assimon MM, et al. Feasibility of Tablet-Based Patient-Reported Symptom Data Collection Among Hemodialysis Patients. Kidney Int Rep. 2020;5(7):1026-39.
21. Greenham L, Bennett PN, Dansie K, Viecelli AK, Jesudason S, Mister R, et al. The Symptom Monitoring with Feedback Trial (SWIFT): protocol for a registry-based cluster randomised controlled trial in haemodialysis. Trials. 2022;23(1):419.
22. Johnson JA, Al Sayah F, Buzinski R, Corradetti B, Davison SN, Elliott MJ, et al. A cluster randomized controlled trial for the Evaluation of routinely Measured PATient reported outcomes in HemodialYsis care (EMPATHY): a study protocol. BMC Health Services Research. 2020;20(1):1-14.
23. Kyte D, Anderson N, Bishop J, Bissell A, Brettell E, Calvert M, et al. Results of a pilot feasibility randomised controlled trial exploring the use of an electronic patient-reported outcome measure in the management of UK patients with advanced chronic kidney disease. BMJ Open. 2022;12(3):e050610.
24. Grove BE, Ivarsen P, de Thurah A, Schougaard LM, Kyte D, Hjollund NH. Remote follow-up using patient-reported outcome measures in patients with chronic kidney disease: the PROKID study - study protocol for a non-inferiority pragmatic randomised controlled trial. BMC Health Serv Res. 2019;19(1):631.
25. Schougaard LMV, Larsen LP, Jessen AMK, Sidenius P, Dørflinger L, de Thurah A, et al. AmbuFlex: tele-patient-reported outcomes (telePRO) as the basis for follow-up in chronic and malignant diseases. Quality of Life Research. 2015;25:525 - 34.
26. Tricco AC, Antony J, Zarin W, Strifler L, Ghassemi M, Ivory J, et al. A scoping review of rapid review methods. BMC Med. 2015;13:224.
27. Greenhalgh T, Peacock R. Effectiveness and efficiency of search methods in systematic reviews of complex evidence: audit of primary sources. BMJ. 2005;331(7524):1064-5.

For peer review only

For peer review only - http://bmjopen.bmj.com/site/about/guidelines.xhtml

28. Horsley T, Dingwall O, Sampson M. Checking reference lists to find additional studies for systematic reviews. Cochrane Database Syst Rev. 2011(8):MR000026.
29. Lefebvre C, Glanville J, Wieland LS, Coles B, Weightman AL. Methodological developments in searching for studies for systematic reviews: past, present and future? Syst Rev. 2013;2:78.
30. Braun V, Clarke V. Can I use TA? Should I use TA? Should I not use TA? Comparing reflexive thematic analysis and other pattern-based qualitative analytic approaches. Counselling & Psychotherapy Research. 2021;21(2):37-47.
31. Braun V, Clarke,V. One size fits all? What counts as quality practice in (reflexive) thematic analysis? Qualitative Research in Psychology. 2020:1-25.
32. Damschroder LJ, Aron DC, Keith RE, Kirsh SR, Alexander JA, Lowery JC. Fostering implementation of health services research findings into practice: a consolidated framework for advancing implementation science. Implement Sci. 2009;4:50.
33. Damschroder LJ, Reardon CM, Opra Widerquist MA, Lowery J. Conceptualizing outcomes for use with the Consolidated Framework for Implementation Research (CFIR): the CFIR Outcomes Addendum. Implement Sci. 2022;17(1):7.
34. QSR International Pty Ltd. NVivo (Version 12) 2018 [Available from: https://www.qsrinternational.com/nvivo-qualitative-data-analysis-software/home.
35. Damschroder LJ, Lowery, J. C,. Evaluation of a large-scale weight management program using the consolidated framework for implementation research (CFIR). Implement Sci. 2013;10(8).
36. Ibrahim H, Liu X, Zariffa N, Morris AD, Denniston AK. Health data poverty: an assailable barrier to equitable digital health care. The Lancet Digital Health. 2021;3(4):e260-e5.
37. Duncanson E, Bennett PN, Viecelli A, Dansie K, Handke W, Tong A, et al. Feasibility and acceptability of e-PROMs data capture and feedback among patients receiving haemodialysis in the Symptom monitoring WIth Feedback Trial (SWIFT) pilot: protocol for a qualitative study in Australia. BMJ Open.10(11):e039014.
38. Morton RL, Lioufas N, Dansie K, Palmer SC, Jose MD, Raj R, et al. Use of patient-reported outcome measures and patient-reported experience measures in renal units in Australia and New Zealand: A cross-sectional survey study. Nephrology. 2020;25(1):14-21.
39. Viecelli AK, Duncanson E, Bennett PN, D'Antoine M, Dansie K, Handke W, et al. Perspectives of Patients, Nurses, and Nephrologists About Electronic Symptom Monitoring With Feedback in Hemodialysis Care. Am J Kidney Dis. 2022;80(2):215-26.
40. Perry LM, Morken V, Peipert JD, Yanez B, Garcia SF, Barnard C, et al. Patient-Reported Outcome Dashboards Within the Electronic Health Record to Support Shared Decision-making: Protocol for Co-design and Clinical Evaluation With Patients With Advanced Cancer and Chronic Kidney Disease. JMIR Res Protoc. 2022;11(9):e38461.
41. Schick-Makaroff K, Mharapara P. Use of Electronic Patient-Reported Outcomes in the Care of Patients with Kidney Failure. Nephrology Nursing Journal: Journal of the American Nephrology Nurses' Association. 2020;47(5):465-72.
42. Schick-Makaroff K, Molzahn A. Strategies to use tablet computers for collection of electronic patient-reported outcomes. Health & Quality of Life Outcomes. 2015;13:2.
43. Schick-Makaroff K, Thummapol O, Thompson S, Flynn R, Karimi-Dehkordi M, Klarenbach S, et al. Strategies for incorporating patient-reported outcomes in the care of

For peer review only - http://bmjopen.bmj.com/site/about/guidelines.xhtml

people with chronic kidney disease (PRO kidney): a protocol for a realist synthesis. Syst Rev. 2019;8(1):20.
44. Schick-Makaroff K, Tate K, Molzahn A. Use of Electronic Patient Reported Outcomes in Clinical Nephrology Practice: A Qualitative Pilot Study. Can J Kidney Health Dis. 2019;6:2054358119879451.
45. Van Der Veer SN, Ercia A, Caskey FJ, Farrington K, Jury F, Rees M, et al. Developing an Intervention to Implement Electronic Patient-Reported Outcomes in Renal Services in the UK. Studies in Health Technology & Informatics. 2020;270:936-40.
46. Knowles SE, Ercia A, Caskey F, Rees M, Farrington K, Van der Veer SN. Participatory co-design and normalisation process theory with staff and patients to implement digital ways of working into routine care: the example of electronic patient-reported outcomes in UK renal services. BMC Health Serv Res. 2021;21(1):706.
47. Breckenridge K, Bekker HL, Gibbons E, van der Veer SN, Abbott D, Briancon S, et al. How to routinely collect data on patient-reported outcome and experience measures in renal registries in Europe: an expert consensus meeting. Nephrol Dial Transplant. 2015;30(10):1605-14.
48. Kyte D, Bishop J, Brettell E, Calvert M, Cockwell P, Dutton M, et al. Use of an electronic patient-reported outcome measure in the management of patients with advanced chronic kidney disease: the RePROM pilot trial protocol. BMJ Open. 2018;8(10):e026080.
49. Wong D, Cao S, Ford H, Richardson C, ice, Belenko D, et al. Exploring the use of tablet computer-based electronic data capture system to assess patient reported measures among patients with chronic kidney disease: a pilot study. BMC Nephrology. 2017;18:1-10.
50. Evans JM, Glazer A, Lum R, Heale E, Mackinnon M, Blake PG, et al. Implementing a patient-reported outcome measure for hemodialysis patients in routine clinical care perspectives of patients and providers on esas-r:Renal. Clinical Journal of the American Society of Nephrology. 2020;15(9):1299-309.
51. Schell JO, Lupu DE. A step in the right direction the promise of proms in routine hemodialysis care. Clinical Journal of the American Society of Nephrology. 2020;15(9):1228-30.
52. Grove BE, Schougaard LMV, Ivarsen PR, Kyte D, Hjollund NH, de Thurah A. Patient-reported outcome measures for clinical decision-making in outpatient follow-up: validity and reliability of a renal disease questionnaire. Journal of Patient-Reported Outcomes. 2021;5(1):1-11.
53. Schougaard L M LLP, Jessen A et al. AmbuFlex: tele-patient-reported outcomes (telePRO) as the basis for follow up in chronic and malignant diseases. Qual Life Res. 2016;25(3):525-34.
54. Flythe JE, Dorough A, Narendra JH, Wingard RL, Dalrymple LS, DeWalt DA. Development and content validity of a hemodialysis symptom patient-reported outcome measure. Qual Life Res. 2019;28(1):253-65.
55. Pittman ZC, John SG, McIntyre CW. Collection of daily patient reported outcomes is feasible and demonstrates differential patient experience in chronic kidney disease. Hemodial Int. 2017;21(2):265-73.
56. Schick-Makaroff K, Wozniak LA, Short H, Davison SN, Klarenbach S, Buzinski R, et al. Burden of mental health symptoms and perceptions of their management in in-centre hemodialysis care: a mixed methods study. J Patient Rep Outcomes. 2021;5(1):111.

For peer review only - http://bmjopen.bmj.com/site/about/guidelines.xhtml

57. Davison SN, Klarenbach S, Manns B, Schnick-Makaroff K, Buzinski R, Corradetti B, et al. Patient-reported outcome measures in the care of in-centre hemodialysis patients. Journal of Patient-Reported Outcomes. 2021;5(1):1-8.
58. Schick-Makaroff K, Wozniak LA, Short H, Davison SN, Klarenbach S, Buzinski R, et al. How the Routine Use of Patient-Reported Outcome Measures for Hemodialysis Care Influences Patient-Clinician Communication: A Mixed-Methods Study. Clin J Am Soc Nephrol. 2022;17(11):1631-45.
59. Guerraoui A, Prezelin-Reydit M, Kolko A, Lino-Daniel M, de Roque CD, Urena P, et al. Patient-reported outcome measures in hemodialysis patients: results of the first multicenter cross-sectional ePROMs study in France. BMC Nephrol. 2021;22(1):357.
60. van der Willik EM, Hemmelder MH, Bart HAJ, van Ittersum FJ, Hoogendijk-van den Akker JM, Bos WJW, et al. Routinely measuring symptom burden and health-related quality of life in dialysis patients: first results from the Dutch registry of patient-reported outcome measures. Clin Kidney J. 2021;14(6):1535-44.
61. van der Willik EM, Meuleman Y, Prantl K, van Rijn G, Bos WJW, van Ittersum FJ, et al. Patient-reported outcome measures: selection of a valid questionnaire for routine symptom assessment in patients with advanced chronic kidney disease - a four-phase mixed methods study. BMC Nephrol. 2019;20(1):344.
62. van der Willik EM, Milders J, Bart JAJ, Bos WJW, van Ittersum FJ, Ten Dam M, et al. Discussing results of patient-reported outcome measures (PROMs) between patients and healthcare professionals in routine dialysis care: a qualitative study. BMJ Open. 2022;12(11):e067044.
63. Schick-Makaroff K, Tate K, Molzahn A. Use of Electronic Patient Reported Outcomes in Clinical Nephrology Practice: A Qualitative Pilot Study. Canadian Journal of Kidney Health & Disease. 2019;6:2054358119879451.
64. Flythe JE, Karlsson N, Sundgren A, Cordero P, Grandinetti A, Cremisi H, et al. Development of a preliminary conceptual model of the patient experience of chronic kidney disease: a targeted literature review and analysis. BMC Nephrology. 2021;22(1):233.
65. Coulter A. Measuring what matters to patients. BMJ. 2017;356:j816.
66. Calvert MJ, Cruz Rivera S, Retzer A, Hughes SE, Campbell L, Molony-Oates B, et al. Patient reported outcome assessment must be inclusive and equitable. Nature Medicine. 2022;28:1120-4.
67. Absolom K, Holch P, Woroncow B, Wright EP, Velikova G. Beyond lip service and box ticking: how effective patient engagement is integral to the development and delivery of patient-reported outcomes. Qual Life Res. 2015;24(5):1077-85.
68. Albers EAC, Fraterman I, Walraven I, Wilthagen E, Schagen SB, van der Ploeg IM, et al. Visualization formats of patient-reported outcome measures in clinical practice: a systematic review about preferences and interpretation accuracy. J Patient Rep Outcomes. 2022;6(1):18.
69. Snyder C, Smith K, Holzner B, Rivera YM, Bantug E, Brundage M, et al. Making a picture worth a thousand numbers: recommendations for graphically displaying patient-reported outcomes data. Qual Life Res. 2019;28(2):345-56.
70. Grossman LV, Feiner SK, Mitchell EG, Masterson Creber RM. Leveraging Patient-Reported Outcomes Using Data Visualization. Appl Clin Inform. 2018;9(3):565-75.

For peer review only - http://bmjopen.bmj.com/site/about/guidelines.xhtml

71. Guha C, Lopez-Vargas P, Ju A, Gutman T, Scholes-Robertson NJ, Baumgart A, et al. Patient needs and priorities for patient navigator programmes in chronic kidney disease: a workshop report. BMJ Open. 2020;10(11):e040617.
72. Zhang R, Burgess ER, Reddy MC, Rothrock NE, Bhatt S, Rasmussen LV, et al. Provider perspectives on the integration of patient-reported outcomes in an electronic health record. JAMIA Open. 2019;2(1):73-80.
73. Anderson NE, McMullan C, Calvert M, Dutton M, Cockwell P, Aiyegbusi OL, et al. Using patient-reported outcome measures during the management of patients with end-stage kidney disease requiring treatment with haemodialysis (PROM-HD): a qualitative study. BMJ Open. 2021;11(8):e052629.
74. Schick-Makaroff K, Levay A, Thompson S, Flynn R, Sawatzky R, Thummapol O, et al. An Evidence-Based Theory About PRO Use in Kidney Care: A Realist Synthesis. The Patient. 2021;15(1):21-38.
75. Gibbons C, Porter I, Goncalves-Bradley DC, Stoilov S, Ricci-Cabello I, Tsangaris E, et al. Routine provision of feedback from patient-reported outcome measurements to healthcare providers and patients in clinical practice. Cochrane Database Syst Rev. 2021;10:CD011589.
76. Tong A, Winkelmayer WC, Wheeler DC, Biesen WV, Tugwell P, Manns B, et al. Nephrologists' perspectives on defining and applying patient-centered outcomes in hemodialysis. Clinical Journal of the American Society of Nephrology. 2017;12(3):454-66.
77. Basch E BL, Kerrigan C L, Velikova G. Implementation of Patient Reported Outcomes in routine medical care. American Society of Clinical Oncology Educational Book. 2018;38:122-34.
78. Rabin BA, Brownson RC, Haire-Joshu D, Kreuter MW, Weaver NL. A glossary for dissemination and implementation research in health. Journal of public health management and practice : JPHMP. 2008;14 2:117-23.
79. Stover AM, Haverman L, van Oers HA, Greenhalgh J, Potter CM, Group IPPiCPISW. Using an implementation science approach to implement and evaluate patient-reported outcome measures (PROM) initiatives in routine care settings. Qual Life Res. 2020;30(11):3015-33.
80. Honeyman M MD, Evans H, Davies A. Digital technology and health inequalities: a scoping review (2020) Cardiff: Public Health Wales NHS Trust; 2020 [Available from: https://phw.nhs.wales/publications/publications1/digital-technology-and-health-inequalities-a-scoping-review/?dm_i=21A8,75FES,2H1914,SZM5X,1.
81. Chapman R, Haroon S, Simms-Williams N, Bhala N, Miah F, Nirantharakumar K, et al. Socioeconomic deprivation, age and language are barriers to accessing personal health records: a cross-sectional study of a large hospital-based personal health record system. BMJ Open. 2022;12(1): e054655.
82. van der Veer SN, Couchoud C, Morton RL. The role of kidney registries in expediting large-scale collection of patient-reported outcome measures for people with chronic kidney disease. Clin Kidney J. 2021;14(6):1495-503.
83. Snyder C W, A.W., eds. Users' Guide to Integrating Patient-Reported Outcomes in Electronic Health Records Baltimore, MD: John Hopkins University; 2017 [Available from: https://www.pcori.org/document/users-guide-integrating-patient-reported-outcomes-electronic-health-records.

For peer review only - http://bmjopen.bmj.com/site/about/guidelines.xhtml

84. Aaronsen N ET, Greenhalgh J, Halyard M, Hess R, Miller D, Reeve B, Santana M, Snyder C. User's Guide to Implementing Patient-Reported Outcomes Assessment in Clinical Practice: International Society of Quality of Life Research; 2015 [Jan 2015]. Available from: https://www.isoqol.org/wp-content/uploads/2019/09/2015UsersGuide-Version2.pdf.
85. Zbrozek A, Hebert J, Gogates G, Thorell R, Dell C, Molsen E, et al. Validation of electronic systems to collect patient-reported outcome (PRO) data-recommendations for clinical trial teams: report of the ISPOR ePRO systems validation good research practices task force. Value Health. 2013;16(4):480-9.
86. Bass M, Morris, S., Neapolitan, R. Utilizing Multidimensional Computer Adaptive Testing to Mitigate Burden With Patient Reported Outcomes. AMIA Annual Symposium proceedings AMIA Symposium,. 2015:320-8.
87. Institute of Applied Health Research UoB. RCAT-Renal Computerised Adaptive Test Birmingham: University of Birmingham; 2022 [Available from: https://tinyurl.com/13vdwh0m.

For peer review only

For peer review only - http://bmjopen.bmj.com/site/about/guidelines.xhtml

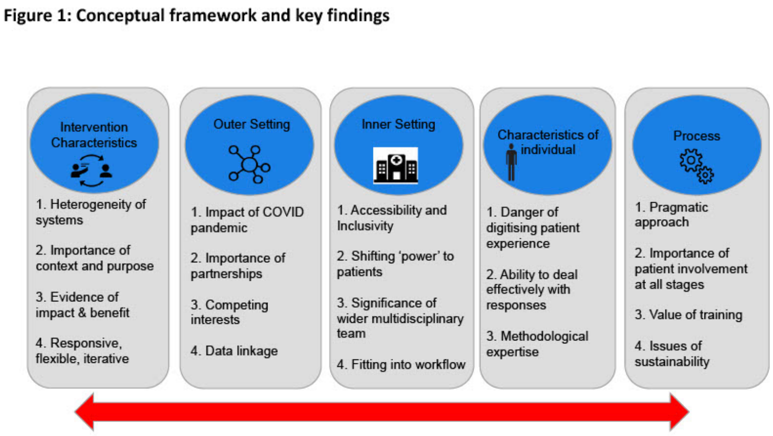

Figure 1: Conceptual Framework and Key Findings

361x256mm (72 x 72 DPI)

Electronic Patient-Reported Outcomes (ePRO) systems used in nephrology settings

1. Electronic Patient-Reported Outcomes (ePRO) Systems used in Nephrology settings

Consent Form.

Please note you may choose to save and continue your survey response at a later time. If you choose this option at the end of each page, you will be directed to enter your email address and we will email you a link for you to access the survey in the future.

1. I confirm that I have read and understood the information in the Participant information sheet version 3.0 dated 06.06.2021 and understand what is expected of me. *

2. I confirm that I have been given the opportunity to ask questions regarding the study, and if asked, the questions were answered to my full satisfaction. *

3. I understand that my participation is voluntary and that I am free to withdraw at any time, but within 5 working days following survey completion (before my data is integrated into the dataset), without giving any reason. *

4. I understand that any personal information will be anonymized but institutional or system identifiers will be used in research outputs i.e. publications/presentations. *

5. I understand that the information collected may be used to support other research in the future and may be shared anonymously with other researchers. *

6. I understand that my personal data will be processed for the purposes detailed above, in accordance with the General Data Protection Regulation and Data Protection Act 2018 *

7. I agree to take part in the survey (please note, if you answer no to this question you will be taken to the end of this survey) *

8. OPTIONAL I am willing to take part in a follow up interview following survey completion, where required.
I understand:

The interview will be audio-recorded and that the transcript of the recording will be shared with researchers working on the above study at the University of Birmingham.

My participation in this interview is voluntary and that I am free to withdraw at any time, up to 10 working days following interview (before my data is integrated into the dataset), without giving any reason.

I will have the opportunity to review any interview transcript and redact any proprietary information before analysis.

I must return reviewed transcripts within 10 working days, otherwise it will be assumed I am happy with the contents.

*

9. Thank you very much for offering to take part in the the optional interview. Please enter your preferred contact details below and a member of the team will be in contact to arrange a convenient date and time. *

First name *

Last name *

Email *

3. ePRO System Characteristics Survey

10. Please enter your name (this information is for administrative purposes only and your data will be pseudonymised) *

11. What was your job title during system development?

12. What is your system called?

Name

Version number

13. Please describe your key responsibilities in the ePRO system development/implementation, please include clinical and non-clinical responsibilities as appropriate.

14. Please state the purpose of your ePRO system (please tick those that apply, there may be more than one primary purpose)

	Primary objective of system	Secondary objective of system	Not a system objective
Improving symptom management (associated with underlying chronic kidney disease)	☐	☐	☐
Improving symptom management (associated with treatment monitoring e.g. Haemodialysis)	☐	☐	☐
Identification of psychosocial problems	☐	☐	☐
To facilitate patient-provider communication	☐	☐	☐
Research	☐	☐	☐
Benchmarking of services	☐	☐	☐
Clinical service commissioning	☐	☐	☐
Support transition of care e.g. to renal replacement therapy or conservative care	☐	☐	☐

If your ePRO system has another purpose, please state

15. What is the population focus of the system (please tick all that apply)

- ☐ Chronic Kidney Disease Stages 1-3
- ☐ Chronic Kidney Disease Stages 4-5 pre dialysis
- ☐ In-centre Haemodialysis
- ☐ Home Haemodialysis
- ☐ Peritoneal Dialysis
- ☐ Transplant
- ☐ Conservative Care

16. When was ePRO system launched?

DD/MM/YYYY

17. How was the system developed?

- ☐ Informatics team within the host organisation (single organisation)
- ☐ Collaboration across two or more organisations
- ☐ Already available Commercial product

Comments:

18. Approximate number of patient users

19. Approximate number of clinician users

20. Primary location of use (please tick all that apply)

- ☐ Primary Care (Community/General Practice clinic)
- ☐ Secondary Care (Hospital clinic)
- ☐ Dialysis Centre
- ☐ Home

21. What PRO Measure (s) are you using?

	PRO Measure
Are you using a new measure?	☐
Are you using an existing measure(s)?	☐
Do you need to use a licence for use?	☐

Comments: Please state name of PRO measure(s)

22. Has the PRO Measure been validated with your patient population? *

- ☐ Yes
- ☐ No

23. How was your ePRO system validated?

- ☐ Formal paper-electronic equivalence testing
- ☐ None
- ☐ Other (please specify):

24. What analysis metric is being used to make or inform clinical decisions?

- ☐ Change from baseline
- ☐ Final value
- ☐ Time to event
- ☐ Other (please specify):

25. How was your ePRO system funded? (you may tick more than one option if mixed model funding used)

- ☐ Commercial
- ☐ Academic
- ☐ Charitable
- ☐ Governmental

Other: Please state

26. What security features does your ePRO system employ? (please tick all that apply)

- ☐ Secure log in (password)
- ☐ Encryption
- ☐ Other (please specify):

6. Administration of PRO assessments/level of automation

27. Is your ePRO system web-based and/or App based (please tick all that apply)

- ☐ Non-responsive website i.e. for display on desk top computer
- ☐ Responsive/mobile website i.e. content displays and function across all devices
- ☐ Mobile App

28. How is the ePRO system accessed? (please tick all that apply)

- ☐ Computer
- ☐ Tablet
- ☐ Interactive Voice Response System
- ☐ Clinic based kiosk
- ☐ Smartphone
- ☐ Other (please specify):

29. Timing of ePRO assessment

- ☐ Prior to clinical assessment
- ☐ Set timepoint weekly
- ☐ Set timepoint monthly
- ☐ Set timepoint 3 monthly
- ☐ Set timepoint 6 monthly
- ☐ Set timepoint annually
- ☐ As required (by patient)
- ☐ Other (please specify):

30. PRO assessment selection: How does your ePRO system select which PRO to use?

- [ ] Automatic (by system) i.e. Provider
- [ ] Patient

Comments:

31. Question Format: How does the ePRO system present questions?

- [ ] One question per page
- [ ] Multiple questions per page
- [ ] Mixed format of single and multiple questions

32. Question advancement: how does the ePRO system move from one question to the next?

- [ ] Mouse click
- [ ] Automatic on completion

33. What page features are available? (please tick all that apply)

- [ ] Progress Bar
- [ ] Visual graphics i.e. graph, diagram, chart
- [ ] Other (please specify):

34. How does your ePRO system avoid missing data during completion? (Please tick all that apply)

- [ ] Allows multiple logins per assessment with automatic save function
- [ ] Allows multiple logins per assessment with save and return later function
- [ ] Allows not applicable (N/A) response
- [ ] Default response pre-selected (pre-populated neutral response)
- [ ] Reminders
- [ ] Other (please specify):

35. Is the ePRO available in more than one language? *

	Yes	No
Is the ePRO available in more than one language?	☐	☐

Comments: Please state available languages

7. Cross cultural validation

36. Has the ePRO measure been validated for use in multiple languages?

- ☐ Yes
- ☐ No

Comments: if yes, please specify languages

37. How does the ePRO system notify that assessments have been completed? (Please tick all that apply)

- ☐ Automated submission notification to patient
- ☐ Automated submission notification to clinicians
- ☐ Email notification to patient from clinical team following review of responses
- ☐ App based notification
- ☐ Other (please specify):

38. How are patients reminded to complete their ePRO assessment? (Please tick all that apply)

- ☐ Email
- ☐ Telephone call
- ☐ Text message: SMS
- ☐ Verbally
- ☐ By letter
- ☐ No reminder
- ☐ App based push notification

39. Does the ePRO system offer flexible system features (please tick all that apply)

- ☐ Web based home and clinic login access
- ☐ Multiple assessment scheduling options
- ☐ Two or more sources for PRO selection (patient/provider)

- [ ] Self identification of important issues by patient (computer adaptive testing functionality)
- [ ] Free text availability
- [ ] Multiple language availability
- [ ] In app or push notifications
- [ ] Other (please specify):

9. ePRO System integration and reporting

40. Please outline the ePRO report content (Please tick all that apply)

- [ ] Current scores including summary and individual scores
- [ ] Longitudinal change
- [ ] Interpretation included in report
- [ ] Cut scores (e.g. low, medium, high)
- [ ] Population norms or reference values
- [ ] Identification of meaningful change
- [ ] Modifiable reports
- [ ] General guidelines
- [ ] Other (please specify):

41. How are ePRO reports accessed? (Please tick all that apply)

- [ ] Via clinical portal/electronic patient health record
- [ ] Via immediate access i.e. summary print out on completion
- [ ] Results restricted to clinical encounter
- [ ] Other (please specify):

42. Who has access to ePRO results/summary reports (please tick all that apply)

- [ ] Patient
- [ ] Clinicians
- [ ] Care Provider
- [ ] Multiple care provider access
- [ ] Other (please specify):

43. Who is responsible for initial review and action of ePRO results?

- [ ] Medical staff
- [ ] Nursing staff
- [ ] Administrative staff
- [ ] Other member of multidisciplinary team

Comments:

44. What form does the clinical response to ePRO report take? (please tick all that apply)

- ☐ Prescribed electronic response dependent on score
- ☐ Clinician/staff follow up; follow up type (face to face, virtual) dependent on score/decision aids
- ☐ Automatic referral to member of multidisciplinary team
- ☐ Automated patient education/message regardless of score
- ☐ Other (please specify):

45. Is there visual presentation of the ePRO scores? (please tick all that apply)

- ☐ Graphical
- ☐ Tabular
- ☐ Numerical
- ☐ Emoticon
- ☐ Colour coded
- ☐ Other (please specify):

46. Does your ePRO system have a safety alert system, dependent on respondent scores? *

- ☐ Yes
- ☐ No

10. Management of safety

47. Management of safety: Who is the intended primary recipient and audience of the ePRO score alert and associated response?

- ☐ Clinician/Staff
- ☐ Patients
- ☐ Care provider
- ☐ Caregiver
- ☐ Multiple recipients i.e. clinicians and patients

Comments:

48. Management of safety: what form do ePRO score alerts take? (please tick all that apply)

- ☐ Email
- ☐ Text message/SMS
- ☐ Telephone call
- ☐ Verbal
- ☐ Real time alert
- ☐ In App notification
- ☐ None

11. Training

49. Do you offer training on the ePRO system? *

- ☐ Yes
- ☐ No

12. Training

50. Who receives training on the ePRO system? (Please tick all that apply)

- ☐ Clinical staff
- ☐ Administrative staff
- ☐ Patients
- ☐ Carers
- ☐ Training available but not specified
- ☐ No formal training available

51. What form does ePRO system training take? (Please tick all that apply)

- ☐ Face to face
- ☐ Online
- ☐ Integrated into ePRO system
- ☐ No formal training

52. Is there a facility for patient education through the ePRO system? (please tick all that may apply)

- ☐ Patient education administered within system
- ☐ Education linked to ePRO scores
- ☐ Automatic documentation of actions
- ☐ No patient education offered

Comments:

13. Evaluation

53. Has your ePRO system been evaluated? *

14. Evaluation

54. What form of evaluation did you undertake?

- ☐ Formative research
- ☐ Pre implementation feasibility testing
- ☐ Progress focused evaluation concurrent to implementation
- ☐ Retrospective evaluation

Comments:

55. Did you include patients in your evaluation?

56. What, if any, changes did you make following the evaluation process

15. Additional features

57. Is there linkage between the ePRO system and other electronic data systems? (Please tick all that apply)

- ☐ Patient Electronic Health Record
- ☐ Appointment/scheduling systems
- ☐ Patient Billing systems
- ☐ No linkage
- ☐ Other (please specify):

58. Accessibility: is the data available to areas outside nephrology settings?

- ☐ Yes
- ☐ No

Comments: If yes, please state

59. Does your ePRO system offer any additional features (please tick all that apply)

- ☐ Quality of care evaluation
- ☐ Patient satisfaction data collected
- ☐ Accreditation reporting
- ☐ None
- ☐ Other (please specify):

60. Is IT support available for your ePRO system?

- ☐ Full
- ☐ Limited
- ☐ User Manual available
- ☐ None

Comments: if Limited, please state what support is offered

16. Contact details

61. Would you like a summary of the findings of this survey?

- [ ] Yes
- [ ] No

If yes, please enter your preferred contact details below

Thank you very much for taking part in this survey.
Would you like to get in touch with the research team?
If you would like any more information, please contact Nicola Anderson at NEA451@student.bham.ac.uk

Nephrology ePRO systems review - Topic Guide

Introduction/Ice breaker

The introduction will set the tone and start building rapport:
Introduce self as a Nurse Researcher with a clinical background specialising in nephrology research and that this interview is being undertaken as part of a clinician doctoral research fellowship funded by the National Institute for Health Research.

Tell respondents the general purpose of the interview

Before the interview, check that the respondents are still happy to take part and have signed the study specific electronic consent form.

Participants will be reminded that all individual self-identifiers will be removed before transcripts are analysed but that the System/institution itself will be named.

1. Icebreaker question: Please outline your role and key responsibilities as a System Lead for the ePRO outlined in your survey responses

2. Can you describe how the ePRO is being implemented in your institution? *Prompts: What was/is your implementation plan? What stage are you at?*

3. What is the purpose of assessment? *Prompt: To inform clinical care, identify symptoms/tailor care, audit, benchmark, real-world evidence, trials Mixture?*

4. Did you use internal/external change agents to help implementation? Did you use peer support/champions to help implementation? *Prompt: Did you get endorsement or support from leaders? If yes, what form did it take, if no, why do you think not?*

5. Did you use an existing PROM? Or develop a new measure? *Prompt: Did you measure the psychometric properties of any existing measure? If a new measure was created – what methodological approach was taken? Use of core outcome sets? COSMIN assessment? Level of patient input? FDA guidance?*

6. Who are the key influential stakeholders for the ePRO? What was your engagement strategy to get these people on board? *Prompts: Stakeholders could be Health care professionals, patients, carers, senior managers, administration staff. Were these individuals involved in c0-designing the system – in particular were patients involved?*

7. What is the general level of receptivity in your organization? *Prompt: Are users (patients/Healthcare Professionals) happy to use the ePRO,*

For peer review only - http://bmjopen.bmj.com/site/about/guidelines.xhtml

are they confident? If not, do you know why not?

8. Were any supports developed to help you implement and use the ePRO?
Prompts: Training resources? Marketing material?> toolkit? Were these resources available digitally, was face to face training made available.

9. What is the priority for getting the ePRO implemented relative to other initiatives that are happening now?
Prompt: for example was it affected by the COVID 19 pandemic?

10. Was the ePRO piloted prior to full-scale implementation?
Prompt: Can you describe the context of the pilot? What worked well? What did not work well?

11. Was the ePRO available in more than one language? Were there mechanisms to allow use by as many patients as possible?
Prompt: were any translations culturally validated?

12. What mechanisms do you have in place to allow maximum accessibility?
Prompt: was the ePRO available for use with patients who have impairments, or can't access IT

13. What direct and indirect costs were incurred to implement the ePRO?
Prompts: Did you need to make changes to infrastructure i.e. IT systems, staffing to support ePRO, adaptions to existing workflow and care delivery systems

14. Did you have sufficient resources to implement the ePRO?
Prompt: if not, what should be prioritised? Links to EPR? Safety features?

15. Do you think the ePRO has improved the way care is being delivered? If yes, how do you think this takes effect?
Prompt: Improved access to services? Help with self-management?, Reduced travel time and expense? To support individual care/tailor care to individual needs, audit, benchmarking, real-world evidence generation – all of these? Other

16. Are you collecting any information to assess impact of the ePRO? If so what type of data?
Prompt: health economic data to assess cost effectiveness, average length of consultation , hospitalisations, death rate, health service interactions, patient and clinician satisfaction

17. What kind of performance measures, policies, regulations, or guidelines influenced the decision to implement the ePRO? Does it provide your organisation with an advantage?
Prompt: Local, state, national measures? Were there incentives to support the

For peer review only - http://bmjopen.bmj.com/site/about/guidelines.xhtml

ePRO

18. Overall has the ePRO been implemented according to the implementation plan?
 Prompt: What worked well (Facilitators), what did not (Barriers), what would you change?

19. Are you aware of other ePROs systems in regular/systematic use in nephrology settings?
 Prompt: If yes, which systems and what are the associated institution? Do you know who the System Lead is?

20. How does your ePRO compare to other similar/existing ePROs in your setting?
 Prompt: Are you aware of any specific advantages/disadvantages of your digital PRO?

21. Do you have anything else to add?

We have now reached the end of the interview. Please be reminded that any personal identifiers will be removed but institutional and system identifiers will be used in any subsequent outputs. You will be given a copy of the transcript after the interview and will have 10 working days to inform the lead researchers of any redactions you wish to make or study withdrawal. Many thanks for your participation.

For peer review only - http://bmjopen.bmj.com/site/about/guidelines.xhtml

Example illustrative quotations

**Intervention characteristics**<br>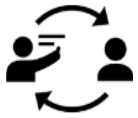	*'huge variety of different PROMs available but no single PROM measured even 50% of the symptoms that were reported by the population. So I think that kind of sends a clear message that maybe there's a gap here for a PROM that's maybe a bit more comprehensive that meets patient's needs. But of course, the challenge of that is you don't want to make it too long or burdensome.' (01)* – **Heterogeneity of measures**<br><br>*'the main learning is that if we want to make ePRO collection a success in renal or in any other specialty, we need to find concrete decisions or concrete situations where ePRO information really adds value. My learning is that just coming ePROs as a concept and saying, 'Now we're going to collect this data for anything,' I think that's not the way to go '(09).* **Importance of context and purpose**<br><br>*'It's a huge investment to make ePROS part of a registry and create that extra platform, and so we need to really provide the evidence that it's going to work before we just outlay everything, don't we?' (010)* **– Evidence of impact & benefit**
**Outer setting**<br>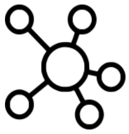	*'The way we designed the study was that we had intentionally iterations planned. We start with a certain package, then look at what works and what doesn't work and then add stuff. So related to what I said at the beginning, I think we started off with focusing on the patient side of things but acknowledging that we would need to do something to improve clinician engagement later on. So, I think I've always envisaged that being part of the later iterations, but we never got there because the study got paused halfway through the second wave' (09)* **– Impact of COVID**<br><br>*'It's really like hospital IT department, we kind of conceived of them as like a vendor who we were purchasing their services to launch something, which I guess is good, really wanted to be full partners, but that means they brought in their whole ideas about what informatics kind of launches should be like and this stuff. Which they were included in the co-production from the very beginning, which was good, but that relationship still isn't totally as good as it could be and so that's a challenge too……. there's always sort of push and pull on the priorities (02)* – **Partnerships and competing interests**<br><br>*'we were really planning to use this existing framework because this platform is integrated and linked to the local EPR and so once patients complete the questionnaires, their scores are displayed for the team' (04)* – **Data linkage**
**Inner setting**<br>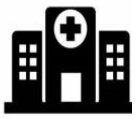	*'And that was one thing that we had thought of going into it, was that any time you have a new sort of feature that's going to be in the clinic, it can actually exacerbate disparities because the patients who are most ready to benefit from that, who are already doing the best probably will, right? And so now they've got this new thing that they can use and the patients that are already doing worse or have the most challenges or barriers, that's just another thing that they're not going to be able to take advantage of…' (02)* – **Accessibility and inclusivity**

For peer review only

	'I think giving the people in these units the opportunity to fill out a survey in their own language also then increases the inclusivity of the trial' (010) - **Accessibility and inclusivity**<br><br>*But they [patients] did use words like feeling empowered to be a partner in the discussion. And obviously bringing up things, they had permission to bring things up and so it would be easier to start a conversation on mental health for example '(012)* – **shifting power to patients**<br><br>*And if I was going to do it over again, I would have taken all the emphasis off the clinicians, and I would have put it on the patients. I would have educated the patients. I would have told them how they could use it. Initiating discussion with the clinician. Giving it to them to share with their clinician or, like I would have turned the tables (03)* **– shifting power to patients**<br><br>*'They [clinicians] felt that when you have the ePRO data in advance of a consultation it allowed them to focus in on the really important aspects around patient symptoms from the outset so maybe more efficient use of time' (01)* – **fitting into workflow**
**Characteristics of individuals**<br><br>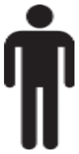	*'we also want to link the whole assessment to response pathways, both self-management support for patients and decision support for the professional teams' (04)* – **Ability to deal with responses**<br><br>*'But I think what I'm resisting is the, to quote Foucault, which is about the technology of self which is going to put it in a box. And say, this is not so bad, this is okay. This is a screaming alert and so the screaming alert gets all of our attention and all of sudden we've 'technologicalised' a life. And we don't want to do that so, I'm worried about moving to the technological simplification of something that isn't simple……….Yes, particularly in our context right, particularly where we see high technological dependence primarily in the nursing role…' (03)* – **Danger of digitising the patient experience**
**Process**<br><br>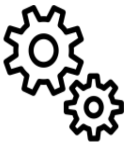	*'so the registry is a really important tool. We have this huge platform where we collect this information and it's already put in there in real time for the most part' (010)* – **Pragmatic approach**<br><br>*'The patient advisory group was really important especially during development. There are a number of times where we sort of went in with a plan and for the intervention, for the ePRO and we changed our plans based on what they were telling us in terms of how this would actually work in practice' (01)* – **Importance of patient involvement**<br><br>*'They got face-to-face training because they needed to understand the system really well and they needed to be able to explain what certain things meant. We did train nurses but that was more in groups. We maybe joined a multidisciplinary team meeting or a team meeting for the nurses in the morning, explaining where they could find the results' (09)* – **Value of training**

For peer review only - http://bmjopen.bmj.com/site/about/guidelines.xhtml

	'I guess the sustainability is the question because you can envision training and so-called patient [involvement] at the launch of this initiative and hoping that it will be cascaded down further and sustained but probably some kind of ongoing training or at least continuing education in services for nurses is going to be necessary. That's also part of a sustainability plan. It needs to become part of the quality systems (04) **– Sustainability**

For peer review only

Reporting checklist for quality improvement in health care.

Based on the SQUIRE guidelines.

Instructions to authors

Complete this checklist by entering the page numbers from your manuscript where readers will find each of the items listed below.

Your article may not currently address all the items on the checklist. Please modify your text to include the missing information. If you are certain that an item does not apply, please write "n/a" and provide a short explanation.

Upload your completed checklist as an extra file when you submit to a journal.

In your methods section, say that you used the SQUIREreporting guidelines, and cite them as:

Ogrinc G, Davies L, Goodman D, Batalden P, Davidoff F, Stevens D. SQUIRE 2.0 (Standards for QUality Improvement Reporting Excellence): revised publication guidelines from a detailed consensus process

		Reporting Item	Page Number
Title			
	#1	Indicate that the manuscript concerns an initiative to improve healthcare (broadly defined to include the quality, safety, effectiveness, patientcenteredness, timeliness, cost, efficiency, and equity of healthcare)	1
Abstract			
	#02a	Provide adequate information to aid in searching and indexing	4
	#02b	Summarize all key information from various sections of the text using the abstract format of the intended publication or a structured summary such as: background, local problem, methods, interventions, results, conclusions	2-3
Introduction			
Problem	#3	Nature and significance of the local problem	5

For peer review only

description			
Available knowledge	#4	Summary of what is currently known about the problem, including relevant previous studies	5-6
Rationale	#5	Informal or formal frameworks, models, concepts, and / or theories used to explain the problem, any reasons or assumptions that were used to develop the intervention(s), and reasons why the intervention(s) was expected to work	8
Specific aims	#6	Purpose of the project and of this report	6
Methods			
Context	#7	Contextual elements considered important at the outset of introducing the intervention(s)	5-6
Intervention(s)	#08a	Description of the intervention(s) in sufficient detail that others could reproduce it	Tables 2-4
Intervention(s)	#08b	Specifics of the team involved in the work	1
Study of the Intervention(s)	#09a	Approach chosen for assessing the impact of the intervention(s)	8
Study of the Intervention(s)	#09b	Approach used to establish whether the observed outcomes were due to the intervention(s)	7-8
Measures	#10a	Measures chosen for studying processes and outcomes of the intervention(s), including rationale for choosing them, their operational definitions, and their validity and reliability	n/a – not assessing success of single intervention
Measures	#10b	Description of the approach to the ongoing assessment of contextual elements that contributed to the success, failure, efficiency, and cost	7-8
Measures	#10c	Methods employed for assessing completeness and accuracy of data	Utilization of mixed methods approach 7-8
Analysis	#11a	Qualitative and quantitative methods used to draw inferences from the data	7-8
Analysis	#11b	Methods for understanding variation within the data, including the effects of time as a variable	7-8
Ethical considerations	#12	Ethical aspects of implementing and studying the intervention(s) and how they were addressed, including, but not limited to, formal ethics review and potential conflict(s) of	31

For peer review only

		interest	
Results			
	#13a	Initial steps of the intervention(s) and their evolution over time (e.g., time-line diagram, flow chart, or table), including modifications made to the intervention during the project	n/a – not reporting single intervention but relevant data in tables 1-4
	#13b	Details of the process measures and outcome	n/a
	#13c	Contextual elements that interacted with the intervention(s)	Purpose of study – reported in pages 9-25
	#13d	Observed associations between outcomes, interventions, and relevant contextual elements	9-25
	#13e	Unintended consequences such as unexpected benefits, problems, failures, or costs associated with the intervention(s).	22
	#13f	Details about missing data	Skipped responses listed in tables reporting survey data
Discussion			
Summary	#14a	Key findings, including relevance to the rationale and specific aims	26-27
Summary	#14b	Particular strengths of the project	4
Interpretation	#15a	Nature of the association between the intervention(s) and the outcomes	n/a – not reporting single intervention
Interpretation	#15b	Comparison of results with findings from other publications	n/a first comparison of different ePRO systems
Interpretation	#15c	Impact of the project on people and systems	26-29
Interpretation	#15d	Reasons for any differences between observed and anticipated outcomes, including the influence of context	26-29
Interpretation	#15e	Costs and strategic trade-offs, including opportunity costs	29

For peer review only

Limitations	#16a	Limits to the generalizability of the work	4
Limitations	#16b	Factors that might have limited internal validity such as confounding, bias, or imprecision in the design, methods, measurement, or analysis	4
Limitations	#16c	Efforts made to minimize and adjust for limitations	Mixed methods approach
Conclusion	#17a	Usefulness of the work	27
Conclusion	#17b	Sustainability	24,28
Conclusion	#17c	Potential for spread to other contexts	30
Conclusion	#17d	Implications for practice and for further study in the field	30-31
Conclusion	#17e	Suggested next steps	31
Other information			
Funding	#18	Sources of funding that supported this work. Role, if any, of the funding organization in the design, implementation, interpretation, and reporting	34

None The SQUIRE 2.0 checklist is distributed under the terms of the Creative Commons Attribution License CC BY-NC 4.0. This checklist can be completed online using https://www.goodreports.org/, a tool made by the EQUATOR Network in collaboration with Penelope.ai

For peer review only

BMJ Open

Global Use of Electronic Patient-Reported Outcome Systems in Nephrology: A Mixed-Methods Study

Journal:	*BMJ Open*
Manuscript ID	bmjopen-2022-070927.R1
Article Type:	Original research
Date Submitted by the Author:	02-May-2023
Complete List of Authors:	Anderson, Nicola; University of Birmingham, Centre for Patient Reported Outcomes Research; University Hospitals Birmingham NHS Foundation Trust, Renal Research<br>Kyte, Derek; University of Worcester,<br>McMullan, Christel; University of Birmingham, Institute of Applied Health Research, Centre for Patient Reported Outcomes Research<br>Cockwell, Paul; Queen Elizabeth Hospital Birmingham, Department of Renal Medicine; University of Birmingham, Division of Infection and Immunity<br>Aiyegbusi, Olalekan Lee; University of Birmingham, Centre for Patient-Reported Outcomes Research; University of Birmingham, NIHR Birmingham Biomedical Research Centre, University of Birmingham and University Hospitals Birmingham NHS Foundation Trust<br>Verdi, Rav; University of Birmingham, Patient Advisory Group Member, Centre for Patient-Reported Outcomes Research (CPROR)<br>Calvert , Melanie ; University of Birmingham, Centre for Patient Reported Outcomes Research and Institute of Applied Health Research; University of Birmingham, NIHR Birmingham Biomedical Research Centre
**Primary Subject Heading**:	Renal medicine
Secondary Subject Heading:	Patient-centred medicine, Qualitative research
Keywords:	Telemedicine < BIOTECHNOLOGY & BIOINFORMATICS, Dialysis < NEPHROLOGY, Quality of Life, REGISTRIES, Patient Reported Outcome Measures, QUALITATIVE RESEARCH

SCHOLARONE™ Manuscripts

I, the Submitting Author has the right to grant and does grant on behalf of all authors of the Work (as defined in the below author licence), an exclusive licence and/or a non-exclusive licence for contributions from authors who are: i) UK Crown employees; ii) where BMJ has agreed a CC-BY licence shall apply, and/or iii) in accordance with the terms applicable for US Federal Government officers or employees acting as part of their official duties; on a worldwide, perpetual, irrevocable, royalty-free basis to BMJ Publishing Group Ltd ("BMJ") its licensees and where the relevant Journal is co-owned by BMJ to the co-owners of the Journal, to publish the Work in this journal and any other BMJ products and to exploit all rights, as set out in our licence.

The Submitting Author accepts and understands that any supply made under these terms is made by BMJ to the Submitting Author unless you are acting as an employee on behalf of your employer or a postgraduate student of an affiliated institution which is paying any applicable article publishing charge ("APC") for Open Access articles. Where the Submitting Author wishes to make the Work available on an Open Access basis (and intends to pay the relevant APC), the terms of reuse of such Open Access shall be governed by a Creative Commons licence – details of these licences and which Creative Commons licence will apply to this Work are set out in our licence referred to above.

Other than as permitted in any relevant BMJ Author's Self Archiving Policies, I confirm this Work has not been accepted for publication elsewhere, is not being considered for publication elsewhere and does not duplicate material already published. I confirm all authors consent to publication of this Work and authorise the granting of this licence.

For peer review only

Global Use of Electronic Patient-Reported Outcome Systems in Nephrology: A Mixed Methods Study

Nicola E Anderson PhD[1,2], Derek Kyte PhD[3] Christel McMullan PhD[1,4.5], Paul Cockwell PhD[7], Olalekan L Aiyegbusi PhD [1,5,6,8,9] Ravinder Verdi [10,] Melanie J Calvert PhD[1,4,5,6,8,9]

[1] Institute of Applied Health Research, Centre for Patient Reported Outcomes Research, University of Birmingham, Birmingham, United Kingdom

[2]Research and Development, University Hospitals Birmingham NHS Foundation Trust

[3] School of Allied Health and Community, University of Worcester, Worcester, UK

[4] NIHR SRMRC, University Hospitals Birmingham NHS Foundation Trust and University of Birmingham, UK

[5]NIHR Blood and Transplant Research Unit (BTRU) in Precision Transplant and Cellular Therapeutics

[6]NIHR Birmingham Biomedical Research Centre, University Hospitals Birmingham NHS Foundation Trust and University of Birmingham, UK

[7]Department of Nephrology, University Hospitals Birmingham NHS Foundation Trust

[8]Birmingham Health Partners Centre for Regulatory Science and Innovation

[9]NIHR Applied Research Collaboration West Midlands, University of Birmingham

[10] Patient Partner, University of Birmingham, UK

Corresponding author: Nicola Anderson n.e.anderson.1@bham.ac.uk

For peer review only

ABSTRACT

Objectives

The use of electronic patient-reported outcome (ePRO) systems to support the management of patients with chronic kidney disease is increasing. This mixed-methods study aimed to comprehensively identify existing and developing ePRO systems, used in nephrology settings globally, ascertaining key characteristics and factors for successful implementation.

Study design

ePRO systems and developers were identified through a scoping review of the literature and contact with field experts. Developers were invited to participate in a structured survey, to summarise key system characteristics including: (i) system objectives; (ii) population; (iii) PRO measures used; (iv) level of automation; (v) reporting; (vi) integration into workflow; and (vii) links to electronic health records/national registries. Subsequent semi-structured interviews were conducted to explore responses.

Setting and participants

Eligible systems included those being developed or used in nephrology settings to assess ePROs and summarise results to care providers. System developers included those with a key responsibility for aspects of the design, development, or implementation of an eligible system.

Analytical Approach

Structured survey data was summarised using descriptive statistics. Interview transcripts were analysed using Codebook Thematic Analysis using domains from the Consolidated Framework for Implementation Research.

For peer review only - http://bmjopen.bmj.com/site/about/guidelines.xhtml

Results

Fifteen unique ePRO systems were identified across seven countries; ten system developers completed the structured survey and seven participated in semi-structured interviews. Despite system heterogeneity, reported features required for effective implementation included early and sustained patient involvement, clinician champions, and expanding existing electronic platforms to integrate ePROs. Systems demonstrated several common features, with the majority being implemented within research settings thereby affecting system implementation readiness for real-world application.

Conclusions

There has been considerable research investment in ePRO systems. The findings of this study outline key system features and factors to support the successful implementation of ePROs in routine kidney care.

For peer review only

For peer review only - http://bmjopen.bmj.com/site/about/guidelines.xhtml

Key Words:

Nephrology, Chronic Kidney Disease, Haemodialysis, Patients Reported Outcomes, Electronic Patient Reported Outcomes, Health Related Quality of Life

Article Summary

Strengths and limitations of this study

- The mixed-methods approach offered a greater understanding of the barriers and facilitators of ePROs implementation, supplementing survey findings on key system characteristics with in-depth interview data from ePROs system developers.
- The electronic survey and interview topic guide was developed with input from stakeholders including clinicians, PROs methodologists and patients.
- The review was restricted to English language publications of ePRO systems. Therefore, it is possible that potentially relevant publications published in other languages were excluded.
- Purposive sampling methods were used due to the limited numbers of people who could act as a primary data source i.e., survey and interview participants could have a clinical or non-clinical role but must have carried key responsibility for aspects of design, development, or implementation of an eligible ePRO system.
- It is possible that other ePRO systems that could be used in nephrology settings were not identified by this study.

For peer review only - http://bmjopen.bmj.com/site/about/guidelines.xhtml

INTRODUCTION

Patients at all stages of chronic kidney disease (CKD), and particularly those undertaking kidney replacement therapy, experience a high symptom burden and often report diminished health-related quality of life (HRQOL) (1-3). However, some symptoms remain under-recognized and unalleviated, leading to increased patient burden (4). HRQOL, is not only an important outcome in itself, it is associated with clinical outcomes such as healthcare utilisation and mortality (5). Symptom burden and impact on HRQOL can be assessed using patient-reported outcomes (PROs), defined as "any report of the status of a patient's health condition that comes directly from the patient, without interpretation of the patient's response by a clinician or anyone" (6). PROs assessing the impact of CKD and associated treatments can be collected electronically (ePROs).

With the increase in telemedicine, accelerated by the COVID-19 pandemic (7, 8), the collection of ePROs has increasing significance. Web-based systems and 'bring your own device' schemes offer patients new ways to report symptoms, function, and HRQOL; they can report PROs in 'real-time' from home or in clinic prior to their consultation. Such use could assist in the management of symptoms, whilst ensuring that healthcare resources are used effectively to maximize impact (9).

There is increasing evidence, particularly from oncology, that ePROs are cost-effective (10), can facilitate shared-decision making, promote self-management, improve symptoms and HRQOL, reduce unplanned hospitalisation and enhance long-term outcomes including survival (11-14). In contrast to paper-based collection, ePRO systems offer enhanced ease of

For peer review only - http://bmjopen.bmj.com/site/about/guidelines.xhtml

use and reduced burden, increased user satisfaction (patient and clinician) and lower rates of missing data. (15-18).

Evidence around implementation of ePROs in nephrology is growing. Studies from North America demonstrate the feasibility of ePRO collection in haemodialysis (HD) settings (16, 19, 20), while the Australian SWIFT (Symptom monitoring With Feedback Trial) (21) and Canadian EMPATHY studies (22) are cluster randomised controlled trials exploring the usefulness of integrating PRO assessments in the clinical management of patients receiving HD. In advanced CKD populations, the RePROM study in the UK piloted the use, and established feasibility, of an ePRO for remote symptom monitoring in real-time (9, 23). The AmbuFlex telepro system is used in practice to manage renal outpatient follow up in Denmark and as a complimentary tool in consultations (24, 25), and is currently being evaluated through the PRO-KID trial: a non-inferiority pragmatic randomised controlled trial investigating the effectiveness of the quality of care, use of resources and patient outcomes associated with PRO-based follow-up in patients with CKD (24).

With this growth of ePRO system research and implementation in CKD globally, there is an opportunity to explore the key characteristics associated with successful implementation to support widespread adoption. The objectives of this study were to (1) comprehensively identify ePRO systems designed specifically for use with CKD populations, including those under development, (2) to explore system characteristics including methods of administration, levels of integration into existing workflow and electronic health records (EHR) and the reporting of ePROs to manage patient safety.

For peer review only

For peer review only - http://bmjopen.bmj.com/site/about/guidelines.xhtml

The aim being to provide a comprehensive classification of core factors which contribute to successful implementation of ePROs in nephrology, including common facilitators and barriers.

STUDY DESIGN

Methods

Predicated upon the research paradigm of pragmatism, with a focus on analysis of study data through the lens of its practical consequences and actionable knowledge (26) this three phased study used mixed methods:

Phase 1 comprised a scoping review identifying ePRO systems and corresponding developers (27). Eligible systems were those being developed, used or under study that were designed for or reconfigured specifically for use in adult nephrology settings but excluding those developed for the management of acute kidney injury and paediatric CKD populations. Systems were also excluded if PROs were not assessed electronically and did not provide a summary of the patients' responses to their care provider for use in routine care, i.e., were being used solely to collect research or population level data or to assess the effectiveness of an intervention.

Eligible systems from any country were included. A system developer could have a clinical or non-clinical role but must have carried key responsibility for aspects of design, development, or implementation of an eligible ePRO system.

Systems were identified through publications in English, including conference abstracts and grey literature; adapting a previously applied search strategy used to investigate nephrology

For peer review only - http://bmjopen.bmj.com/site/about/guidelines.xhtml

PROs measures (28). Databases (OVID Medline, EMBASE, APA Psychinfo and CINAHL) were searched from dates of inception to 15th Dec 2021 (see supplementary file 1 for example search strategy) followed by ongoing 'pearl' and 'snowballing' methodology, i.e., searching from known key references and checking reference lists (29-31). Field experts were also consulted. Data from identified publications were used to create a structured survey exploring key system characteristics (See supplementary file 2), and to identify system developers.

Phase 2 involved survey administration via an online survey platform Smart Survey ™ (www.smartsurvey.co.uk) which was piloted prior to use. System developers were invited to participate via email. The survey focused on system design and software features, integration of e-PRO collection and reporting in clinical care.

Phase 3: Optional follow up semi-structured interviews were undertaken online via videoconferencing software with survey participants, or a suitable team member nominated by the original survey respondent. The purpose of these interviews was to expand on survey responses and allow more detailed system/organisational enquiry. All follow up interviews used a piloted study-specific topic guide (see supplementary file 3). Participants were given the opportunity to check and review transcripts, to comment and avoid disclosure of any proprietary information. Interviews were conducted by NEA, a renal research nurse with previous experience of conducting qualitative research (32). This study was undertaken as part of a Doctoral Research Fellowship, which was disclosed in the participant information sheet and during the consent process. Some interview participants were previously known to the interviewer, as experts in the field of study. Maintenance of a reflective diary, memos

For peer review only

For peer review only - http://bmjopen.bmj.com/site/about/guidelines.xhtml

and field notes, along with discussions with the study management team, were used to minimise the influence of any prior relationships on data analysis.

Analysis

Data derived from the Phase 1 review were abstracted and charted (see table 1), and descriptive statistics from the Phase 2 structured survey were tabulated (see tables 2-4). Analysis of Phase 3 interview data was undertaken using Codebook Thematic Analysis (33, 34) and the domains from the Consolidated Framework for Implementation Research (CFIR) (Figure 1). Primary data analysis was conducted by lead author (NEA) with a second investigator reviewing coding (CMcM) for consistency and appropriateness. The CFIR is a determinant framework that can be used to identify and delineate contextual factors (i.e., barriers or facilitators) that influence the outcome of implementation efforts. (35, 36). Computer Assisted Qualitative Data Analysis Software CASDAQ (QSR NVIVO 12 (37)) was used to facilitate qualitative data analysis. Thematic analysis using a framework approach was chosen to systematically identify and analyse patterns of meaning within data, with the aim of highlighting the most salient features (33). The CFIR was used to gain insight into the overall effectiveness of the ePRO systems and associated implementation strategies. It is possible to rate constructs using the CFIR to undertake organisational comparison via Qualitative Comparative Analysis (QCA) (38). Due to the heterogeneity of systems, their context and stage of development, there was a danger of oversimplifying complex, dynamic descriptions of implementation processes and contexts so this aggregated data approach was not taken. Due to time constraints participant checking of findings was undertaken by one participant only.

For peer review only

For peer review only - http://bmjopen.bmj.com/site/about/guidelines.xhtml

Patient and Public Involvement (PPI)

Patient partners were involved in the design of this study, and co-authored the final manuscript; where they specifically highlighted the importance of ensuring ePROs do not exacerbate existing health inequalities due to digital inequalities (lack of digital access or skills).

RESULTS

The scoping review identified 14 ePRO systems across seven countries from 43 papers and one developing system identified by field experts, totalling 15 systems: Canada n=4, United States of America n=3, United Kingdom n=4, Denmark n=1, France n=1, The Netherlands n=1, Australia n=1 (Table 1). Supplementary file S1 includes the PRISMA ScR Diagram

For peer review only

Table 1: Overview of ePRO systems (see supplementary file 4 for additional system data)

Name of System	Country	Population	In current use Yes/No	System ePROs
**ANZDATA SWIFT PROMs Module (21, 39-42)**	Australia	In-centre HD	Yes – pilot study reported, Cluster RCT in progress	SONG-HD fatigue measure<br>IPOS-Renal<br>EQ-5D-5L
**cPRO-Collaborate Kidney Care (43)**	United States of America	CKD Stages 1-3<br>CKD Stages 4-5 pre dialysis	Yes – clinical demonstration model	PROMIS and FACIT measures
**Cambian (ePRO-KIDNEY) (16, 17, 44-48)**	Canada	Home HD, PD	No – platform used within reported research projects	Patient Assessment of Care for Chronic Conditions-20 questionnaire (PACIC-20)<br>Kidney Disease Quality of Life-36 (KDQOL-36)<br>Edmonton Symptom Assessment System renal (ESAS-r), EQ-5D-5L
**OPT-ePRO (49-52)**	United Kingdom	CKD Stages 1-3, CKD Stages 4-5 pre dialysis,In-centre HD, Home HD, PD<br>Transplant, Conservative Care	No – pilot study reported	POS-S-RENAL, EQ-5D-5L
**Penguin (Cievert Ltd)<br>(no published data)**	United Kingdom	Transplant	Yes- Research pilot in progress	New study specific measure
**RePROM (9, 23, 53-55)**	United Kingdom	CKD Stages 4-5 pre dialysis	No – pilot study reported.	New study specific measure
**Unnamed System 1 (56)**	Canada	In-centre HD, Transplant	No – in development	PROMIS CATs (unspecified)(56)
**'Your symptoms matter' (57, 58)**	Canada	In-centre HD	Yes – pilot study reported	ESAS-r: Renal

For peer review only - http://bmjopen.bmj.com/site/about/guidelines.xhtml

***Survey data only - developers declined participation in optional FU interview**				
**Ambuflex (PRO-KID** Non-inferiority pragmatic randomised controlled trial evaluating incorporating Ambuflex platform) **(24, 59-63)**	Denmark	**Ambuflex**<br>CKD Stages 4-5 pre dialysis, Conservative Care<br><br>**(PRO-KID**<br>CKD Stages 4-5 pre dialysis, home)	**Ambuflex** - Yes<br><br>**(PRO-KID** – trial in progress,	**Ambuflex** –<br>27 item Renal Disease questionnaire (59)<br>**(PRO-KID**<br>1. Renal-specific domains, items from • KDQOL-SF • EORTC • SF-GH1<br>2. Additional research PROs (24)
**SMaRRT-HD™ (20, 64)**	United States of America	In-centre HD	Unknown – feasibility study reported	SMaRRT-HD ™ – Study specific measure
****System identified only – no survey or interview data included in evidence synthesis**				
**"Derby Evaluation of Illness" (65)**	United Kingdom	CKD stages 4/5 pre-dialysis, in-centre HD, PD	Unknown – feasibility study reported	Six separate domains assessed by VAS: general wellbeing, pain, sleep, breathing, energy, fistula function and appetite.
**EMPATHY study (22, 66-68)**	Canada	In-centre HD	Yes – Cluster RCT in progress	ESAS-r: Renal/IPOS-Renal and/or the EQ-5D-5L
**K-Pal (19)**	United States of America	Patient's ≥60 years of age with ESRD on HD	Unknown – feasibility study reported	Short-Form McGill Pain Questionnaire 2 (SF-MPQ-2), Patient Health Questionnaire-9 (PHQ-9), Generalized Anxiety Disorder 7 Item Survey (GAD-7), Dialysis Symptom Index (DSI), KDQOL-36
**eNephro (69)**	France	CKD stage 3B/4, stage 5D CKD on dialysis (PD/HD), Transplant	Unknown – Pragmatic RCT (anticipated date of study completion Dec 2018 NCTO 2082093)	Symptoms, Hospitalization Anxiety Depression Scale (HADS) KDQoL 36 , ReTransQoL (for transplant patients) to assess trial outcomes
**Dutch Renal Registry (70-72)**	The Netherlands	Patients undergoing dialysis	Yes – part of development of national registry of PROMs	Dialysis Symptom Index (DSI), SF-12

For peer review only - http://bmjopen.bmj.com/site/about/guidelines.xhtml

Ten system developers responded to the survey (66% response rate). System developers held varying academic and clinical academic research posts, all demonstrating outcomes methodology expertise. Key responsibilities ranged from funding acquisition and stakeholder engagement, to overseeing system development, piloting, and evaluation. Seven developers agreed to a follow up interview: one expert delegated this task to a suitable member of their study team; three declined. Reasons for not taking part in the follow up interview were not collected. Interviews ranged from 42 to 82 minutes in length (mean 63 minutes).

Key system characteristics

Table 2 outlines characteristics of surveyed systems and system objectives.

For peer review only

Table 2: System Characteristics

System Characteristic/Feature<br>*multiple response options total ≠ 100%	Number<br>n=10<br>(Skipped response)	Response %
System launched	(0)	
In development	1	10%
<1 year	1	10%
1-5 years	5	50%
>5 years	3	30%
Primary Objective of System*	(0)	
Improving symptom assoc with CKD	7	70%
Improving symptom assoc with treatment	5	50%
Psychosocial care	0	0%
Facilitate communication	4	40%
Research	3	30%
Benchmarking	0	0%
Commissioning	0	0%
Support transition of care	0	0%
Secondary Objective of System*	(0)	
Improving symptom assoc CKD	2	20%
Improving symptom assoc treatment	4	40%
Psychosocial care	8	80%
Facilitate communication	6	60%
Research	4	40%
Benchmarking	2	20%
Commissioning	1	10%
Support transition of care	2	20%
How developed?	(0)	
In house informatics team	5	50%
Collaboration across sectors	4	40%
Commercial product	1	10%
Funding Source*	(0)	
Commercial	1	10.0%
Academic	4	40.0%
Charitable	3	30.0%
Government	3	30.0%
Primary location of use*	(0)	
Primary Care (Community/General Practice clinic)	10.0%	1
Secondary Care (Hospital clinic)	80.0%	8
Dialysis Centre	60.0%	6
Home	20.0%	2
System platform*	(0)	
Non-responsive website	2	20.0%
Responsive/mobile website	8	80.0%
Mobile application	2	20.0%

For peer review only - http://bmjopen.bmj.com/site/about/guidelines.xhtml

System Access*	(0)	
Computer	7	70.0%
Tablet	10	100.0%
Interactive Voice Response System	0	0.0%
Clinic Based kiosk	2	20.0%
Smartphone	7	70.0%
PRO selection*	(2)	
Automatic (by system) i.e., Provider	8	100%
By Patient	0	0.0%
System security features*	(1)	
Secure Log in	9	100.0
Encryption	7	77.8%
two factor log in	1	11.1%
Unsure	1	11.1%
Page features*	(1)	
Progress Bar	6	66.7%
Visual graphics i.e., graph, diagram, chart	4	44.4%
Automatic save function, Option to save and return later	1	11.1%
Training available	(0)	
Yes	9	90%
No	1	10%
Who receives training? *	(1)	
Clinical staff	9	100.0%
Administrative staff	4	44.4%
Patients	7	77.8%
Carers	0	0.0%
Form of e PRO system training*	(1)	
Face to face	7	77.8%
Online	7	77.8%
Integrated into ePRO system	2	22.2%
Facility for patient education*	(2)	
Administered in system	4	50.0%
Education linked to ePRO scores	1	12.5%
Automatic documentation of action	0	0.0%
No patient education offered	4	50.0%
In development	1	12.5%
Has the system been evaluated?	(0)	
Yes	7	70%
No	3	30%

The most common primary objective was symptom management associated with CKD and/or renal replacement therapy by HD. However, systems were implemented across kidney disease stages and treatment modalities (see Table 1 and supplementary file 4 for

For peer review only - http://bmjopen.bmj.com/site/about/guidelines.xhtml

additional system data). Aggregate uses of ePROs for benchmarking or commissioning of services were not common; one system collected satisfaction with care data (73), and two others assessed quality of care (21, 43). There was variation in funding source; academic (40%), government (30%) and charitable funding (30%), with 70% of systems being developed in the last five years. Half of the systems were developed in-house by local informatics teams, with the rest reporting collaborations across sectors with registries and technology companies. Systems allowed ePROs to be completed in different settings; the most common setting for completion was hospital clinic (80%). Only one system could be accessed by primary care (43).

One surveyed system, Ambuflex, had been implemented into routine practice, but was undergoing additional evaluation (25); all other systems were at varying stages of study with most having conducted pilot/feasibility testing. Two identified systems were being evaluated within a cluster randomised controlled trial (21, 22); while another was being evaluated in a co-designed demonstration trial (43). Tablet was the most popular mode of administration. Two systems offered a clinic-based kiosk facility (25, 57). Training was available or planned for clinical staff in all systems but focused largely on system access and functionality. Seven systems had developed training materials for patients and four for administrative staff. Four systems had patient education material administered within the system, only one of these linked self-management guidelines to scores (9). Only 60% of systems were currently linked with the EHR.

All the surveyed systems involved patients during design and implementation; from measure choice to evaluation. The ePRO Collaborate system used a co-design process to refine the

For peer review only

For peer review only - http://bmjopen.bmj.com/site/about/guidelines.xhtml

244 dashboard and the constructs being assessed in their demonstration trial, combining the use
245 of healthcare coproduction frameworks with stakeholder engagement (43). Developers of
246 the OPT-ePRO system used participatory co-design guided by Normalisation Process Theory
247 to guide design and planning with clinicians, patients and caregivers (48).

248 Patients with CKD at varying stages were involved in the decision to create a study specific
249 questionnaire for the RePROM study; in order to capture outcomes they felt were most
250 relevant (9).

251 **Data collection and assessment**

252 Table 3 summarises key data collection functions.

253 **Table 3: Data collection and assessment**

Data Collection/Assessment *multiple response options total ≠ 100%	Number n=10 (Skipped responses)	Response%
**Measure development***	(0)	
System uses new PROM	5	50%
System uses existing PROM	7	70%
**Timing of Assessment***	(0)	
Prior to clinical assessment	4	40.0%
Set time point weekly	0	0.0%
Set time point monthly	1	10.0%
Set time point 3 monthly	3	30.0%
Set time point 6 monthly	1	10.0%
Set time point annually	0	0.0%
As required (by patient)	2	20.0%
Other	4	40.0%
**Question format**	(1)	
One question per page	3	33.3%
Multiple questions per page	0	0.0%
Mixed format of single and multiple questions	6	66.7%
**Question Advancement**	(1)	
Mouse click	6	66.7%

For peer review only - http://bmjopen.bmj.com/site/about/guidelines.xhtml

Automatic on completion	3	33.3%
Analysis Metric*	(0)	
Change from baseline	7	70.0%
Final value	6	60.0%
Time to event	0	0.0%
Other (system defined)	2	20.0%
Avoidance of Missing data*	(1)	
Allows multiple logins per assessment with automatic save function	0	0.0%
Allows multiple logins per assessment with save and return later function	3	33.3%
Allows not applicable (N/A) response	2	22.2%
Default response pre-selected (pre-populated neutral response)	0	0.0%
Reminders	5	55.6%
Other: i.e., Mandatory fields	4	44.4%
Notification of completed assessments*	(0)	
Automated submission notification to patient	1	10.0%
Automated submission notification to clinicians	4	40.0%
Email notification to patient from clinical team following review of responses	2	20.0%
App based notification	0	0.0%
Other: None/unsure	3	30.0%
Patient Reminder format*	(0)	
Email	5	50.0%
Telephone call	2	20.0%
Text message: SMS	2	20.0%
Verbally	5	50.0%
By letter	3	30.0%
No reminder	0	0.0%
App based push notification	1	10.0%
Flexible system features*	(1)	
Web based home and clinic login access	7	77.8%
Multiple assessment scheduling options	3	33.3%
Two or more sources for PRO selection (patient/provider)	1	11.1%
Self-identification of important issues by patient (CAT functionality)	3	33.3%
Free text availability	8	88.9%
Multiple language availability	3	33.3%
In app or push notifications	0	0.0%
Other: facility for multiple Lang/CAT currently not used	1	11.1%

254

For peer review only - http://bmjopen.bmj.com/site/about/guidelines.xhtml

Similarly, half the developers reported using a newly developed PRO measure, either completely new or a mix of existing tools. Only three surveyed systems had ePRO availability in languages other than English (20, 21, 57, 64), although several developers reported plans for future translations, dependent on funding. There were a mixture of mechanisms to remind patients to complete their ePROs, with email (50%) and verbal reminders (50%) being most popular. Reminders were seen as the best way to avoid missing data (55.6%), but some systems offered a save and return function.

Frequency of reporting was varied, with most common timing of assessment being prior to a clinical assessment (40%). Some systems did have set time points ranging from one to six monthly. Two systems allowed patients to complete as required (9, 57).

System reporting

Table 4 outlines system reporting features.

Table 4: System reporting

System reporting/Integration<br>*multiple response options total ≠ 100%	Number<br>n=10<br>(Skipped responses)	% Response
ePRO report access*	(0)	
Via clinical portal/electronic patient health record	7	70.0%
Via immediate access i.e., summary print out on completion	4	40.0%
Results restricted to clinical encounter	0	0.0%
Other: Registry, emailed PDF to patient	2	20.0%
ePRO report content*	(0)	
Current scores including summary and individual scores	7	70.0%
Longitudinal change	8	80.0%
Interpretation included in report	3	30.0%
Cut scores (e.g., low, medium, high)	4	40.0%

For peer review only

For peer review only - http://bmjopen.bmj.com/site/about/guidelines.xhtml

Population norms or reference values<br>Identification of meaningful change<br>Modifiable reports<br>General guidelines<br>Other i.e., colour coding	2<br>1<br>1<br>4<br>3	20.0%<br>10.0%<br>10.0%<br>40.0%<br>30.0%
**Who has access to report/summary***<br>Patient<br>Clinicians<br>Care Provider<br>Multiple care provider access<br>Researcher	(0)<br>10<br>10<br>3<br>3<br>1	 100.0%<br>100.0%<br>30.0%<br>30.0%<br>10.0%
**Who is responsible for initial review & action**<br>Medical staff<br>Nursing staff<br>Administrative staff<br>Other member of multidisciplinary team	(1)<br>4<br>5<br>0<br>0	 44.4%<br>55.6%<br>0.0%<br>0.0%
**Form of clinical response***<br>Prescribed electronic response dependent on score<br>Clinician/staff follow up; follow up type (face to face, virtual) dependent on score/decision aids<br>Automatic referral to member of multidisciplinary tea<br>Automated patient education/message regardless of score<br>Self-management support resource dependent on score	(0)<br>2<br>9<br><br>1<br>1<br><br>1	 20.0%<br>90.0%<br><br>10.0%<br>10.0%<br><br>10.0%
**Visual presentation of PROs scores***<br>Graphical<br>Tabular<br>Numerical<br>Emoticon<br>Colour coded<br>Other: No score/being developed for longitudinal data	(0)<br>8<br>3<br>3<br>1<br>5<br>2	 80.0%<br>30.0%<br>30.0%<br>10.0%<br>50.0%<br>20.0%
**Safety Alert System (eAlert) – dependent on PRO score**<br><br>Yes<br>No	(0)<br><br>3<br>7	 <br>30.0%<br>70.0%
**Intended recipient for eAlert**<br>Clinician/Staff<br>Patients<br>Care provider<br>Caregiver<br>Multiple recipients i.e., clinicians and patients	(7)<br>3<br>0<br>0<br>0<br>0	 100%<br>0.0%<br>0.0%<br>0.0%<br>0.0%
**Form of eAlert***<br>Email	(7)<br>2	 66.7%

For peer review only - http://bmjopen.bmj.com/site/about/guidelines.xhtml

**Text message/SMS**	0	0.0%
**Telephone call**	0	0.0%
**Verbal**	0	0.0%
**Real time alert**	2	66.7%
**In App notification**	0	0.0%
**None**	0	0.0%

All systems allowed patients and clinicians direct access to ePRO results or summary reports. Doctors (44.4%) and nurses (55.6%) were responsible for initial review and action of results. Follow-up (face to face, virtual, telephone) was largely dependent on scores. Electronic PRO reports contained current scores (summary and individual scores) in 70% of systems, 80% presented longitudinal changes and 40% offered cut scores (low, medium, high). One system was part of an overall dashboard with the option for patients to combine ePRO scores with lab values in a graph (43) and the Ambuflex PRO questionnaire was also viewed alongside other available clinical data, such as biochemistry, blood pressure, body weight, as a decision aid together to determine whether the patient needs a phone call or a face-to-face visit (24).

In terms of data visualisation, graphical presentation was most popular (80%) with 50% of ePROs using colour coding to highlight significance of scores. Less frequently used approaches of score presentation were tables (30%), numerical scores (30%) and emoticons (10%). Assistance with interpretation within the report was uncommon, but 30% of ePROs had developed a safety alert system dependent on respondent scores (9, 20, 25). The RePROM system produced real-time email alerts directly to the kidney nursing team (9), while the SMaRRT HD^{TM} system generated email alerts, for which the severity alert thresholds required adaption so that they were frequent enough to ensure symptom capture without overburdening the workflow with emails (range 2-22/week) (20). The

For peer review only

AmbuFlex system keeps track by giving patients a red, yellow and green status with non-responders presented to clinicians on an alert list (25). The eNephro study by Thilly et al (2017) was investigating the cost-effectiveness of home telemonitoring in patients with CKD. This system included a clinical decision support algorithm to detect situations of risk; relying largely on biological parameters, it could alert in cases of a complication associated with predefined symptoms (69)

Follow up interviews

Semi-structured interviews were undertaken with seven ePROs developers, allowing clarification of survey responses: to explore the development process, and identify contextual factors working for and against implementation. All interviews took place online using videoconferencing software (Zoom) with no-one else present. Analysis generated rich data which are presented in line with the CFIR key domains (35). Figure 1 outlines the conceptual framework (Illustrative quotations to support the key findings are provided in supplementary file 5).

Intervention Characteristics:

Analysis highlighted the heterogeneity of the systems. However, developers agreed that systems need to be flexible, responsive, and follow an iterative development strategy. All interview participants felt that their systems would need future refinement, whether simple adaption to increase language availability or more complex programming to allow real-time symptom alerting or provision of self-management tools. Knowledge of context (population and setting) and purpose of ePRO data collection should be clearly defined and

For peer review only - http://bmjopen.bmj.com/site/about/guidelines.xhtml

communicated to all stakeholders including patients. Developers acknowledged that the evidence of ePROs benefit is less mature in nephrology than other clinical specialities. However, participants indicated ePROs could deliver a relative advantage over current care delivery: improving communication, supporting symptom management, enhancing self-care and shared decision making, with potential to reduce healthcare utilisation. However, all participants recognised the challenges, particularly of clinician engagement. They cited clinician anxieties around potential increases in workload, attendance to symptoms and problems unrelated to kidney disease. Consequently, several developers chose not to include real-time safety alerts associated with severe symptom reports.

Several systems used currently available infrastructure, utilising existing platforms, registries, and validated measures. Whilst this offered the advantage of existing expertise and reduced cost, this was offset by restrictions on local adaption and global comparability due to lack of harmonisation.

Outer Setting:

All ePROs system programs had been impacted and delayed by the COVID-19 pandemic. Several developers reported that key staff including informatics teams had reprioritised activity and had less availability to support ePRO implementation. However, they stated that the rapid shift to virtual consultations and recognition of the potential impact of ePROs in managing care, particularly outpatient planning, invigorated interest in ePROs.

Developers highlighted the importance of partnership and collaboration – across clinical, academic, and commercial organisations. Registries were seen as an ideal collection

For peer review only

For peer review only - http://bmjopen.bmj.com/site/about/guidelines.xhtml

platform. However, developers indicated multi-organisational approaches could lead to competing interests and governance issues that can cause delays.

Developers acknowledged the value of integration of ePROs directly into the EHR, but not all systems had this functionality. Some still required manual entry, which was reported as time consuming, costly and would not support real-time care. Many systems were standalone in nephrology secondary care, meaning data would not ‘follow’ patients across care settings and could not be used to manage transition of care needs.

Inner Setting:

Developers suggested potential unintended consequences of ePRO implementation included further widening of health inequities, due to inadequate accessibility to digital devices, variable digital competence, and lack of inclusivity caused by poor availability of systems in multiple languages or failure to assess cultural compatibility. Some developers were investigating such outcomes in particularly vulnerable groups, e.g., elderly, ethnic minorities.

Developers cited the continued prevalence of biomedical models of care, despite general acceptance of the need to move to person-centred care, as a barrier to ePRO implementation. They indicated approaches for shifting ‘power’ to patients by focusing on patient understanding of ePROs and involvement at all stages of design and implementation, along with patient peer support to counteract cultural barriers.

Doctors were the professional group most often involved in review and action of ePRO responses, but several systems directed ePRO scores to nursing staff or physician assistants

For peer review only - http://bmjopen.bmj.com/site/about/guidelines.xhtml

as the initial handler. Resources needed to be available to allow the engagement of the wider multi-disciplinary team including supportive and rehabilitation services.

While clinicians were largely receptive to ePROs, workflow compatibility was seen as crucial to uptake, and this was reflected in the preference for a focus on symptom management as primary objective of capture. Several systems included free text options to report symptoms or problems and clinicians needed reassurance that this would not significantly impact workload. Participants reported that pilot study data suggested free text responses did not adversely increase workload but provided another mechanism for patient/provider communication, likewise with initial data on use of symptom report alerts.

Characteristics of Individuals:

Participants discussed the importance of measuring outcomes which matter most to patients but emphasized the hypothetical danger of over digitising the patient experience, meaning that clinicians risk responding to scores alone rather than listening and connecting with the patient to further interpret ePRO data. Several participants looked forward to a future when ePROs might be given similar status to other forms of medical data. They noted clinicians frequently discuss burden associated with the collection and use of ePROs but did not consider similar factors associated with non-essential blood or invasive tests.

Developers reflected on the varied ability to deal effectively with responses, both at an organisational and individual level. They recognised the heavy and complex workload within the speciality, and while some systems offered management guides or further resources,

For peer review only

participants were not all in favour of prescriptive treatment decision algorithms, some preferring to allow the exercise of clinical judgement.

The Process of Implementation:

Systems were in different stages of development and use, with some being part of wider research or quality improvement programmes. A pragmatic approach to implementation was often described: for example, measures were chosen according to what was already being used and what patients would be prepared to complete. Existing platforms that had been previously assessed for governance and regulatory compliance were exploited. Implementation readiness for use in routine care was affected by most systems being investigated outside a real-world context. Sustainability was an issue: future planning would need to include activities undertaken by research personnel including administration tasks, completion support and training. Long term IT and informatics support needs to be factored into costing for maintenance and adaption.

All developers stated the importance of patient involvement at all stages and outlined different ways patients had offered input and insight from co-design/production to research participation in usability testing. Staff dedicated to engagement were useful, ensuring extended community groups were involved.

Education and training associated with the knowledge needed to support ePROs appeared to be under-developed when compared to other aspects of system design. Training resources were often in development and current iterations focused predominantly on system use and

For peer review only

For peer review only - http://bmjopen.bmj.com/site/about/guidelines.xhtml

did not extend to interpreting or actioning responses. It was recognised that training was time consuming and needed to be ongoing.

Overarching themes: Barriers and Facilitators to effective implementation

Across all CFIR constructs, analysis identified potential barriers and facilitators. Barriers included the culture associated with biomedical models, variable clinical engagement particularly from doctors, and governance issues. Participants suggested facilitators included the development of patient driven systems, utilisation of clinical champions, who need both time and belief to promote the system, and adapting existing resources.

DISCUSSION

This review summarises and integrates survey and interview data on the features and implementation factors of 15 nephrology ePRO systems used across seven countries with Box 1 outlining key priorities for successful implementation.

Box 1: Key priorities for successful implementation

Macro level priorities (National) – formation of national collaborative groups to promote equitable and sustainable uptake of ePROs systems that have multiple applicationsincluding support from professional bodies	Harmonisation of collection platforms and goverance systems e.g., National Registry – allowing data linkage to other healthcare settings i.e. primary care<br>Harmonisation of measures (including item banks)<br>Harmonisation of methods of interpretation and analysis metrics<br>High quality research to demonstrate empirical evidence of benefit including real world evaluation<br>Continued research on psychometric properties and interpretation of measures<br>Investigation of new technologies i.e. CAT<br>Endorsement of key guidelines such as the PCORI Users' Guide to Integrating Patient-Reported Outcomes in Electronic Health Records (74), ISOQOL Implementing PROs in clinical practice (75) and the ISPOR Validation of Electronic Systems to Collect Patient-Reported

For peer review only - http://bmjopen.bmj.com/site/about/guidelines.xhtml

	Outcome (PRO) Data Recommendations (76).<br>Provision of adequate resources
Meso level priorities (organisational) - key contributors to include clinicians, patients, carers, IT, informatics, QA, governance departments - Local systems specificity to maximise implementation and minimise effects on workflow	Involve all stakeholders – define and communicate key system objectives<br>Use flexible system design to facilitate local, regional and national compatability<br>Support localised adaption i.e. clinic level and resourcing<br>EHR compatability<br>Allow varying modes of administration, including paper<br>Reminders – varying forms (email, app alert etc.)<br>Alert systems – develop pathways and algorithms for action that compliment workflow<br>High levels of automation to reduce workload<br>Data collection features – need efficient, easy user experience<br>Easy to access, save, submit and review<br>Optimise data interpretation and visualisation<br>Infrastructure support to deliver 'holistic care' – treatment and care algorithms<br>Appointment and support for clinical 'champions'<br>Patient peer support via 'Patient Navigators'<br>Training packages to support implementations for all users<br>Sustained IT support<br>Ongoing evaluation programmes<br>Localised education on the management of long term conditions
Micro level priorities (Individual)<br>Involve all key stakeholders and undertake meaningful PPI activity to ensure ePROs are accessible and inclusive for population	Identify local needs i.e., language availability requirements<br>Provide support for those with poor health or digital literacy (need an assessment of needs) Consider Multi-media PROMs<br>Optimise accessibility – allow onsite completion via kiosk or tablet, enable 'bring your own device'<br>Evalution by patients to ensure systems are not over-digitising the patient experience by not discussing responses with individuals.<br>Meaningful follow up of PROs responses, tailored to individual needs

For peer review only

The majority of surveyed systems prioritised symptom management over other uses of ePROs such as benchmarking and commissioning (see table 2). While some systems were designed to collect ePROs across CKD stages and treatment modalities, patients undergoing HD were the most common population for ePROs use. According to recent reviews, patients with CKD report between 56-68 common and/or severe signs and symptoms, with the exact symptom burden often determined by stage of disease and treatment (3, 77). Participants in

For peer review only - http://bmjopen.bmj.com/site/about/guidelines.xhtml

418 this study reflected that symptom management was a good primary objective on which to
419 concentrate early implementation efforts, with capability to add additional functionality and
420 use.

421 None of the surveyed systems were reported as being developed to align with national
422 quality measures, such as twice yearly In-Center Hemodialysis Consumer Assessment of
423 Healthcare Providers and Systems (ICH CAHPS) (78) and annual assessment of HRQOL using
424 the KDQOL-36 included in the U.S. Medicare End Stage Renal Disease Quality Incentive
425 Program (79). However, increased utilisation of national registries to collect ePROs for
426 routine care and population level use (80), highlights the potential to assess HRQoL and
427 symptoms through a quality registry.

428 **Stakeholder involvement**

429 This study highlights the increased development of nephrology ePROs in the last five years,
430 with all developers reporting positive and negative effects of the COVID-19 pandemic. While
431 there were delays to implementation due to reprioritisation of activity, the increased drive
432 to deliver patient centred care via telemedicine has enhanced attention on ePROs.

433 Patient and public involvement (PPI) is a prerequisite to inclusive and equitable PROs
434 research (81) and the meaningful involvement of patients in ePROs implementation
435 programs is crucial to effective uptake (82). Well-established PPI groups, reflective of the
436 total population, can offer advice on measure choice, language requirements and support
437 resources. Patient input in design of software features including reporting and data
438 visualization can ensure data is interpreted correctly (83) using various devices such as
439 graphs, emoticons and heat maps (84, 85). Patient 'navigators' can offer health literacy and

review only

For peer review only - http://bmjopen.bmj.com/site/about/guidelines.xhtml

communication peer support for successful digital completion (86). All the surveyed systems had involved patients in aspects of development from measure choice to evaluation.

While other important contributors to ePROs implementation are quality assurance, governance, informatics, and information technology departments, any successful ePROs system will need effective clinician ‘buy in’. This study identified this as a key challenge, particularly from those clinicians not involved in ePROs research. Clinical staff state they are anxious about disruptions to already tight workflows and while ePROs are seen as a useful conversation starter, clinicians fear ePROs lack clinical utility for decision making, citing the current lack of empirical evidence on the benefits of using this data in nephrology (32, 87). Indicating a need for research to improve the evidence-base of ePROs use at individual and aggregated levels (88), including studies which employ cluster designs and utilise techniques to maintain allocation concealment (89). Studies included in this review are going some way to provide this evidence.

For peer review only

Developers described utilising the assistance of ‘clinician champions’; individuals with both the time and influence to drive ePRO implementation. These champions would encourage clinicians to view ePRO scores as key health data, in the same way as a biomarker or a lab test, to manage care.

Studies have highlighted tension from clinicians in their ability to respond to ePROs, whether due to workload or belief that their scope of practice should be confined to matters related to nephrology (32, 41, 90). Some clinicians indicate they are more comfortable assessing, rather than actively managing, psychosocial symptoms (57). While holistic care is advocated within kidney care, it is not necessarily supported within the system and multidisciplinary

For peer review only - http://bmjopen.bmj.com/site/about/guidelines.xhtml

approaches and further training in managing mental health problems is needed (66). Although research from other specialities suggests patient encounters using ePROs do not take longer (91), studies on workflow changes and impact are required. Training in all systems was predominantly linked to ePROs system functionality; formal interpretation guidance beyond provision of cut scores or basic colour coding was rare or 'to be developed'. Electronic questionnaires which use and calculate scores or colour codes specifically for decision aids for treatment are considered medical devices, and must comply with relevant legislation (61). Most systems offered or were developing self-management guidance for patients. Effective training needs analysis at the planning stage is crucial to assess the support needed by the multidisciplinary team to respond to ePROs. Generally, systems were being developed iteratively, with training and support materials being developed or expanded after launch and this may be counterproductive to early and sustained engagement from clinical teams.

Further research is needed to investigate how we interpret PROs for CKD management and event prediction; such as dialysis start time, the impact of changing dialysis modality, or the decision to undertake a conservative care pathway.

Real-world implementation

A shift to implementation science for guidance on how to promote adoption, enhance readiness and optimise use of ePROs in real-world settings will also identify any unintended consequences of use, such as exacerbation of health inequalities and potential over digitisation of the patient experience (92, 93).

For peer review only - http://bmjopen.bmj.com/site/about/guidelines.xhtml

Many groups who are already subject to disadvantage and worse health outcomes are also subject to digital exclusion (94). Flexible systems enhance accessibility, offering patients' choice on time, frequency, mode of administration and place of completion. Electronic PROs are commonly completed in a healthcare facility via tablet, where completion support is available. However, onsite completion may impact workflow and support assistance traditionally delivered by research staff will need to be factored into sustainability plans. Home completion may need to be incentivised and alternative means of completion should be offered as required i.e., voice activation response systems, hardcopy by post; following assessment of local population needs. Combinations of digital inclusion approaches are likely needed, to support patients with the digital and health literacy skills required to negotiate both their electronic health records (EHR) and any corresponding ePRO data (95) and offer access to hardware (81, 94).

Early findings from the EMPATHY study stress the difficulty in implementing ePROs in HD settings, indicating that routine PROs use failed to demonstrate a significant improvement in patient-clinician communication (68). Whilst these findings suggested relatively good communication pre-trial, the qualitative data offered potential reasons for no effect: insufficient patient and clinician understanding of the purpose of PROs, challenges with administration, inconsistencies with PROs as communication tools, and limited perceived value. Highlighting that evaluation of current ePROs systems should be investigating implementation outcomes (i.e., fidelity, appropriateness, acceptability, feasibility, reach, adoption, and sustainability) (20, 43), as well as whether ePROs are excluding already disadvantaged groups (49). Evaluation using implementation science methodology, while formally investigating the effectiveness of ePROs interventions, offers a potential avenue to

For peer review only

For peer review only - http://bmjopen.bmj.com/site/about/guidelines.xhtml

build the evidence base on benefit while supporting adoption, adaption and sustainability (96).

While effort was made to comprehensively identify nephrology ePRO systems for inclusion, a key methodological limitation was the possibility that systems which were not referenced in the literature, available in English, would be missed. This method placed reliance on appropriate and reliable indexing systems. However, consultation with experts in the field, including study participants, to identify suitable systems aimed to minimise these effects. It is suggested that as ePROs systems proliferate in healthcare settings, an international prospective register of clinical ePROs systems could offer one solution for knowledge transfer and collaboration, but issues of ownership, resource and up-keep would need to be addressed. Additionally, while the sample sizes were small, sampling was purposive, with cases being selected by virtue of their capacity to provide richly-textured information, relevant to the phenomenon under investigation (97). The prevailing concept for sample size in qualitative research is 'saturation', however this study has been guided by the concept of 'information power' i.e., the larger information power the sample holds, the lower N is needed (98). The specificity of experiences and knowledge offered by the participants for an in-depth analysis meant the actual sample held adequate information power to develop new knowledge, referring to the aim of this study.

Areas for future research

Data linkage is central to multiple usages and harmonisation of collection platforms and associated measures will support this. Integration of ePROs within the EHR can offer 'one stop' access for clinicians and patients, if given access. Extended accessibility across

For peer review only

For peer review only - http://bmjopen.bmj.com/site/about/guidelines.xhtml

healthcare providers would allow long-term monitoring across the kidney care continuum. A study of daily ePROs collection reported specific events such as fistula formation and modality changes led to PRO changes, demonstrating PROs can capture differential patient experience in CKD (65). This study highlights the need to both implement measures that are sensitive and responsive enough to detect clinically relevant change, such as disease progression, over both short and long term, whilst considering how best to minimise respondent burden.

New technologies offer a potential solution, computerized adaptive tests (CATs) offer sophisticated measure delivery using algorithms based on item response theory to personalize ePROs for patients. CAT based measures are shorter, more accurate, and efficient (99). None of the reviewed systems were currently using CAT administration, however, several developers reported this was where they saw the future of ePROs and were actively researching this area; a renal specific item bank and associated CAT is currently being developed for use in the UK (100).

To conclude, whilst there has been considerable research investment in the development of ePROs, to measure CKD symptoms and HRQOL, the next step is to accelerate the implementation gap between research and practice; this study supports this objective by outlining key system features and exploring factors to optimise the delivery ePROs in routine care settings within nephrology.

For peer review only

For peer review only - http://bmjopen.bmj.com/site/about/guidelines.xhtml

Ethical Approval:

Ethics approval was issued on 06/07/2021 by University of Birmingham Science, Technology, Engineering and Mathematics Ethical Review Committee: ERN_21-0495

Contributorship statement:

NEA, MC, CMcM, PC, OLA and DK were involved in the conception of the study, and methodological design. NEA conducted data acquisition. NEA and CMcM undertook analysis and interpretation. NEA wrote the first and revised drafts of the manuscript. RV is a Patient Partner, involved in study design and review of key study documents including protocol, participant information sheet, survey and topic guide. All authors were involved in the final approval of the version to be submitted and published, and agree to be accountable for all aspects of the work in ensuring that questions related to the accuracy or integrity of any part of the work were appropriately investigated and resolved. NEA is responsible for the overall content as the guarantor.

Competing interests:

NEA receives funding from the National Institute of Health and Care Research (NIHR) under its Clinical Doctoral Research Fellowship Programme (Grant Reference ICA-CDRF-2018-04-ST2-027), NIHR Applied Research Collaboration (ARC), West Midlands and declares personal fees from GlaxoSmithKline (GSK) outside the submitted work.

MC is Director of the Birmingham Health Partners Centre for Regulatory Science and Innovation, Director of the Centre for the Centre for Patient Reported Outcomes Research and is a National Institute for Health and Care Research (NIHR) Senior

For peer review only

For peer review only - http://bmjopen.bmj.com/site/about/guidelines.xhtml

Investigator. MC receives funding from the NIHR Birmingham Biomedical Research Centre, NIHR Surgical Reconstruction and Microbiology Research Centre, NIHR Blood and Transplant Research Unit (BTRU) in Precision Transplant and Cellular Therapeutics, and NIHR ARC West Midlands at the University of Birmingham and University Hospitals Birmingham NHS Foundation Trust, Health Data Research UK, Innovate UK (part of UK Research and Innovation), Macmillan Cancer Support, SPINE UK,UKRI, UCB Pharma, Janssen, GSK and Gilead. MC has received personal fees from Astellas, Aparito Ltd, CIS Oncology, Takeda, Merck, Daiichi Sankyo, Glaukos, GSK and the Patient-Centered Outcomes Research Institute (PCORI) outside the submitted work.

DK reports grants from Macmillan Cancer Support, Innovate UK, the NIHR, NIHR Birmingham Biomedical Research Centre, and NIHR SRMRC at the University of Birmingham and University Hospitals Birmingham NHS Foundation Trust, and personal fees from Merck and GSK outside the submitted work. DK has received funding from the NIHR and Kidney Research UK and is Chief Investigator for the RePROM and RCAT studies. CM receives funding from the National Institute for Health Research (NIHR) Surgical Reconstruction and Microbiology Research Centre, the NIHR Birmingham-Oxford Blood and Transplant Research Unit (BTRU) in Precision Transplant and Cellular Therapeutics, Innovate UK, and has received personal fees from Aparito Ltd outside the submitted work. OLA receives funding from the NIHR Birmingham Biomedical Research Centre (BRC), NIHR Applied Research Collaboration (ARC), West Midlands, NIHR Birmingham-Oxford Blood and Transplant Research Unit (BTRU) in Precision Transplant and Cellular Therapeutics at the University of Birmingham and University Hospitals Birmingham NHS Foundation, Innovate UK (part of UK Research and Innovation), Gilead Sciences Ltd, Janssen pharmaceuticals, Inc., and Sarcoma UK. OLA

For peer review only

For peer review only - http://bmjopen.bmj.com/site/about/guidelines.xhtml

declares personal fees from Gilead Sciences Ltd, GlaxoSmithKline (GSK) and Merck outside the submitted work.

The views expressed are those of the author(s) and not necessarily those of the NHS, the NIHR or the Department of Health and Social Care. The study sponsor and funders have no role in study design, including collection, management, analysis, and interpretation of data; writing of the report and the decision to submit the report for publication.

Funding:

This manuscript refers to independent research funded by the National Institute for Health and Care Research (NIHR) under its Clinical Doctoral Research Fellowship Programme (Grant Reference ICA-CDRF-2018-04-ST2-027)

Data sharing statement:

De identified data may be available upon reasonable request via the corresponding author (ORCID 0000-0002-0614-3198).

Supplementary material

File 1: Nephrology ePROs search strategy_PRISMA ScR diagram

File 2: Structured survey

File 3: ePRO System Review CFIR Interview Topic Guide version 4.0 final

File 4: Table Overview of ePROs systems with additional data

File 5: Example illustrative quotations

For peer review only

For peer review only - http://bmjopen.bmj.com/site/about/guidelines.xhtml

613 **Word count: 5048**

For peer review only - http://bmjopen.bmj.com/site/about/guidelines.xhtml

References

1. Almutary H, Bonner, A, Douglas, C. Symptom burden in Chronic Kidney Disease: a review of recent literature. Journal of Renal Care. 2013;39(3):140-50.

2. Lowney AC, Myles HT, Bristowe K, Lowney EL, Shepherd K, Murphy M, et al. Understanding What Influences the Health-Related Quality of Life of Hemodialysis Patients: A Collaborative Study in England and Ireland. J Pain Symptom Manage. 2015;50(6):778-85.

3. Fletcher BR, Damery S, Aiyegbusi OL, Anderson N, Calvert M, Cockwell P, et al. Symptom burden and health-related quality of life in chronic kidney disease: A global systematic review and meta-analysis. PLoS Med. 2022;19(4):e1003954.

4. Anderson NE, Calvert M, Cockwell P, Dutton M, Kyte D. The Use of Patient-Reported Outcomes in Patients Treated With Maintenance Hemodialysis: A Perspective. Am J Kidney Dis. 2019;74(3):399-406.

5. Tang E, Bansal A, Novak M, Mucsi I. Patient-Reported Outcomes in Patients with Chronic Kidney Disease and Kidney Transplant-Part 1. Front Med (Lausanne). 2017;4:254.

6. United States of America Department of Health and Human Services Food and Drug Administration. Guidance for industry. Patient reported outcomes measaures: Use in medical product development to support labeling claims.; 2009 Dec 2009. Report No.: http://www.fda.gov/Drugs/GuidanceComplianceRegulatoryInformation/Guidances/default.htm.

7. Hull SA, Rajabzadeh V, Thomas N, Hoong S, Dreyer G, Rainey H, et al. Do virtual renal clinics improve access to kidney care? A preliminary impact evaluation of a virtual clinic in East London. BMC Nephrol. 2020;21(1):10.

8. Young A, Orchanian-Cheff A, Chan CT, Wald R, Ong SW. Video-Based Telemedicine for Kidney Disease Care. Clinical Journal of the American Society of Nephrology. 2021;16(12):1813-23.

9. Kyte D, Anderson N, Auti R, Aiyegbusi OL, Bishop J, Bissell A, et al. Development of an electronic patient-reported outcome measure (ePROM) system to aid the management of patients with advanced chronic kidney disease. J Patient Rep Outcomes. 2020;4(1):55.

10. Lizée TB, Ethan ; Trémolières, Pierre ; Voog, Eric ; Domont, Julien ; Peyraga, Guillaume ; Urban, Thierry ; Bennouna, Jaafar ; Septans, Anne-Lise ; Balavoine, Magali ; Detournay, Bruno ; Denis, Fabrice. Cost-Effectiveness of Web-Based Patient-Reported Outcome Surveillance in Patients With Lung Cancer. Journal of Thoracic Oncology. 2019;14(6):1012-21.

11. Velikova G, Booth L, Smith AB, Brown PM, Lynch P, Brown JM, et al. Measuring quality of life in routine oncology practice improves communication and patient well-being: a randomized controlled trial. J Clin Oncol. 2004;22(4):714-24.

12. McCann L, Maguire R, Miller M, Kearney N. Patients' perceptions and experiences of using a mobile phone-based advanced symptom management system (ASyMS) to monitor and manage chemotherapy related toxicity. Eur J Cancer Care (Engl). 2009;18(2):156-64.

13. Basch E, Deal AM, Dueck AC, Scher HI, Kris MG, Hudis C, et al. Overall Survival Results of a Trial Assessing Patient-Reported Outcomes for Symptom Monitoring During Routine Cancer Treatment. JAMA. 2017;318(2):197-8.

14. Basch E, Stover , A, M, Schrag, D, Chung, M, Jansen, J, et al. Clinical Utility and User Perceptions of a Digital System for Electronic Patient-Reported Symptom Monitoring During

For peer review only

For peer review only - http://bmjopen.bmj.com/site/about/guidelines.xhtml

Routine Cancer Care: Findings From the PRO-TECT Trial. JCO Clinical Cancer Informatics. 2020;4:947-57.
15. Meirte J, Hellemans N, Anthonissen M, Denteneer L, Maertens K, Moortgat P, et al. Benefits and Disadvantages of Electronic Patient-reported Outcome Measures: Systematic Review. JMIR Perioper Med. 2020;3(1):e15588.
16. Schick-Makaroff K, Molzahn A. Brief Communication: patient satisfaction wit the use of tablet computers: a pilot study in two outpatient home dialysis clinics. Canadian Journal of Kidney Health and Disease. 2014;1(22).
17. Schick-Makaroff K, Molzahn AE. Evaluation of real-time use of electronic patient-reported outcome data by nurses with patients in home dialysis clinics. BMC Health Serv Res. 2017;17(1):439.
18. Aiyegbusi OL, Nair D, Peipert JD, Schick-Makaroff K, Mucsi I. A narrative review of current evidence supporting the implementation of electronic patient-reported outcome measures in the management of chronic diseases. Therapeutic Advances in Chronic Disease. 2021;12.
19. Gabbard J, McLouth CJ, Brenes G, Claudel S, Ongchuan S, Burkart J, et al. Rapid Electronic Capturing of Patient-Reported Outcome Measures in Older Adults With End-Stage Renal Disease: A Feasibility Study. Am J Hosp Palliat Care. 2021;38(5):432-40.
20. Flythe JE, Tugman MJ, Narendra JH, Dorough A, Hilbert J, Assimon MM, et al. Feasibility of Tablet-Based Patient-Reported Symptom Data Collection Among Hemodialysis Patients. Kidney Int Rep. 2020;5(7):1026-39.
21. Greenham L, Bennett PN, Dansie K, Viecelli AK, Jesudason S, Mister R, et al. The Symptom Monitoring with Feedback Trial (SWIFT): protocol for a registry-based cluster randomised controlled trial in haemodialysis. Trials. 2022;23(1):419.
22. Johnson JA, Al Sayah F, Buzinski R, Corradetti B, Davison SN, Elliott MJ, et al. A cluster randomized controlled trial for the Evaluation of routinely Measured PATient reported outcomes in HemodialYsis care (EMPATHY): a study protocol. BMC Health Services Research. 2020;20(1):1-14.
23. Kyte D, Anderson N, Bishop J, Bissell A, Brettell E, Calvert M, et al. Results of a pilot feasibility randomised controlled trial exploring the use of an electronic patient-reported outcome measure in the management of UK patients with advanced chronic kidney disease. BMJ Open. 2022;12(3):e050610.
24. Grove BE, Ivarsen P, de Thurah A, Schougaard LM, Kyte D, Hjollund NH. Remote follow-up using patient-reported outcome measures in patients with chronic kidney disease: the PROKID study - study protocol for a non-inferiority pragmatic randomised controlled trial. BMC Health Serv Res. 2019;19(1):631.
25. Schougaard LMV, Larsen LP, Jessen AMK, Sidenius P, Dørflinger L, de Thurah A, et al. AmbuFlex: tele-patient-reported outcomes (telePRO) as the basis for follow-up in chronic and malignant diseases. Quality of Life Research. 2015;25:525 - 34.
26. Kelly LM, Cordeiro M. Three principles of pragmatism for research on organizational processes. Methodological Innovations. 2020;13(2).
27. Tricco AC, Antony J, Zarin W, Strifler L, Ghassemi M, Ivory J, et al. A scoping review of rapid review methods. BMC Med. 2015;13:224.

For peer review only - http://bmjopen.bmj.com/site/about/guidelines.xhtml

28. Aiyegbusi OL, Kyte D, Cockwell P, Marshall T, Gheorghe A, Keeley T, et al. Measurement properties of patient-reported outcome measures (PROMs) used in adult patients with chronic kidney disease: A systematic review. PLoS One. 2017;12(6):e0179733.

29. Greenhalgh T, Peacock R. Effectiveness and efficiency of search methods in systematic reviews of complex evidence: audit of primary sources. BMJ. 2005;331(7524):1064-5.

30. Horsley T, Dingwall O, Sampson M. Checking reference lists to find additional studies for systematic reviews. Cochrane Database Syst Rev. 2011(8):MR000026.

31. Lefebvre C, Glanville J, Wieland LS, Coles B, Weightman AL. Methodological developments in searching for studies for systematic reviews: past, present and future? Syst Rev. 2013;2:78.

32. Anderson NE, McMullan C, Calvert M, Dutton M, Cockwell P, Aiyegbusi OL, et al. Using patient-reported outcome measures during the management of patients with end-stage kidney disease requiring treatment with haemodialysis (PROM-HD): a qualitative study. BMJ Open. 2021;11(8):e052629.

33. Braun V, Clarke V. Can I use TA? Should I use TA? Should I not use TA? Comparing reflexive thematic analysis and other pattern-based qualitative analytic approaches. Counselling & Psychotherapy Research. 2021;21(2):37-47.

34. Braun V, Clarke,V. One size fits all? What counts as quality practice in (reflexive) thematic analysis? Qualitative Research in Psychology. 2020:1-25.

35. Damschroder LJ, Aron DC, Keith RE, Kirsh SR, Alexander JA, Lowery JC. Fostering implementation of health services research findings into practice: a consolidated framework for advancing implementation science. Implement Sci. 2009;4:50.

36. Damschroder LJ, Reardon CM, Opra Widerquist MA, Lowery J. Conceptualizing outcomes for use with the Consolidated Framework for Implementation Research (CFIR): the CFIR Outcomes Addendum. Implement Sci. 2022;17(1):7.

37. QSR International Pty Ltd. NVivo (Version 12) 2018 [Available from: https://www.qsrinternational.com/nvivo-qualitative-data-analysis-software/home.

38. Damschroder LJ, Lowery, J. C,. Evaluation of a large-scale weight management program using the consolidated framework for implementation research (CFIR). Implement Sci. 2013;10(8).

39. Duncanson E, Bennett PN, Viecelli A, Dansie K, Handke W, Tong A, et al. Feasibility and acceptability of e-PROMs data capture and feedback among patients receiving haemodialysis in the Symptom monitoring WIth Feedback Trial (SWIFT) pilot: protocol for a qualitative study in Australia. BMJ Open.10(11):e039014.

40. Morton RL, Lioufas N, Dansie K, Palmer SC, Jose MD, Raj R, et al. Use of patient-reported outcome measures and patient-reported experience measures in renal units in Australia and New Zealand: A cross-sectional survey study. Nephrology. 2020;25(1):14-21.

41. Viecelli AK, Duncanson E, Bennett PN, D'Antoine M, Dansie K, Handke W, et al. Perspectives of Patients, Nurses, and Nephrologists About Electronic Symptom Monitoring With Feedback in Hemodialysis Care. Am J Kidney Dis. 2022;80(2):215-26.

42. Duncanson EB, P. N.; Viecelli, A. K.; Dansie, K.; Greenham, L. M.; Tong, A.; Jesudason, S.; McDonald, S. P.; Morton, R. L. Mini Orals: Feasibility and acceptability of electronic patient reported outcome measures (e-proms) data capture in the symptom monitoring

For peer review only - http://bmjopen.bmj.com/site/about/guidelines.xhtml

with feedback trial (SWIFT) pilot: Perspectives of nephrologists, nurses and patients. Nephrology. 2020;25:35.
43. Perry LM, Morken V, Peipert JD, Yanez B, Garcia SF, Barnard C, et al. Patient-Reported Outcome Dashboards Within the Electronic Health Record to Support Shared Decision-making: Protocol for Co-design and Clinical Evaluation With Patients With Advanced Cancer and Chronic Kidney Disease. JMIR Res Protoc. 2022;11(9):e38461.
44. Schick-Makaroff K, Mharapara P. Use of Electronic Patient-Reported Outcomes in the Care of Patients with Kidney Failure. Nephrology Nursing Journal: Journal of the American Nephrology Nurses' Association. 2020;47(5):465-72.
45. Schick-Makaroff K, Molzahn A. Strategies to use tablet computers for collection of electronic patient-reported outcomes. Health & Quality of Life Outcomes. 2015;13:2.
46. Schick-Makaroff K, Tate K, Molzahn A. Use of Electronic Patient Reported Outcomes in Clinical Nephrology Practice: A Qualitative Pilot Study. Can J Kidney Health Dis. 2019;6:2054358119879451.
47. Silbernagel P, Sawatzky R, Klarenbach S, Czupryn J, Iradukunda R, Palfreyman S, et al. Patient-Reported Outcomes in Clinical Kidney Practice: Development of Education Sessions for Clinicians. Journal of Pain and Symptom Management. 2018;56(6).
48. Schick-Makaroff K, Sawatzky R, Kwon JY, Lee L, Cohen SR, Czupryn J, et al. A process evaluation in home dialysis: Electronic patient reported outcomes in clinical kidney practice (ePRO Kidney). Quality of Life Research. 2022;31(Supplement 2):S67.
49. Van Der Veer SN, Ercia A, Caskey FJ, Farrington K, Jury F, Rees M, et al. Developing an Intervention to Implement Electronic Patient-Reported Outcomes in Renal Services in the UK. Studies in Health Technology & Informatics. 2020;270:936-40.
50. Knowles SE, Ercia A, Caskey F, Rees M, Farrington K, Van der Veer SN. Participatory co-design and normalisation process theory with staff and patients to implement digital ways of working into routine care: the example of electronic patient-reported outcomes in UK renal services. BMC Health Serv Res. 2021;21(1):706.
51. van der Veer SN, Couchoud C, Morton RL. The role of kidney registries in expediting large-scale collection of patient-reported outcome measures for people with chronic kidney disease. Clin Kidney J. 2021;14(6):1495-503.
52. van der Veer SN, Aresi G, Gair R. Incorporating patient-reported symptom assessments into routine care for people with chronic kidney disease. Clinical Kidney Journal. 2017;10(6):783-7.
53. Aiyegbusi OL, Kyte D, Cockwell P, Marshall T, Dutton M, Slade A, et al. Using Patient-Reported Outcome Measures (PROMs) to promote quality of care and safety in the management of patients with Advanced Chronic Kidney disease (PRO-trACK project): a mixed-methods project protocol. BMJ Open.7(6):e016687.
54. Kyte D, Bishop J, Brettell E, Calvert M, Cockwell P, Dutton M, et al. Use of an electronic patient-reported outcome measure in the management of patients with advanced chronic kidney disease: the RePROM pilot trial protocol. BMJ Open. 2018;8(10):e026080.
55. Aiyegbusi OL, Kyte D, Cockwell P, Marshall T, Dutton M, Walmsley-Allen N, et al. Development and usability testing of an electronic patient-reported outcome measure (ePROM) system for patients with advanced chronic kidney disease. Computers in Biology & Medicine.101:120-7.

For peer review only - http://bmjopen.bmj.com/site/about/guidelines.xhtml

56. Wong D, Cao S, Ford H, Richardson C, ice, Belenko D, et al. Exploring the use of tablet computer-based electronic data capture system to assess patient reported measures among patients with chronic kidney disease: a pilot study. BMC Nephrology. 2017;18:1-10.

57. Evans JM, Glazer A, Lum R, Heale E, Mackinnon M, Blake PG, et al. Implementing a patient-reported outcome measure for hemodialysis patients in routine clinical care perspectives of patients and providers on esas-r:Renal. Clinical Journal of the American Society of Nephrology. 2020;15(9):1299-309.

58. Schell JO, Lupu DE. A step in the right direction the promise of proms in routine hemodialysis care. Clinical Journal of the American Society of Nephrology. 2020;15(9):1228-30.

59. Grove BE, Schougaard LMV, Ivarsen PR, Kyte D, Hjollund NH, de Thurah A. Patient-reported outcome measures for clinical decision-making in outpatient follow-up: validity and reliability of a renal disease questionnaire. Journal of Patient-Reported Outcomes. 2021;5(1):1-11.

60. Schougaard L M LLP, Jessen A et al. AmbuFlex: tele-patient-reported outcomes (telePRO) as the basis for follow up in chronic and malignant diseases. Qual Life Res. 2016;25(3):525-34.

61. Hjollund NHI, Larsen LP, de Thurah AL, Grove BE, Skuladottir H, Linnet H, et al. Patient-reported outcome (PRO) measurements in chronic and malignant diseases: ten years' experience with PRO-algorithm-based patient-clinician interaction (telePRO) in AmbuFlex. Qual Life Res. 2023.

62. Grove BE, Valen Schougaard LM, Ivarsen P, Hjollund NH, de Thurah A, Mejdahl CT. Remote follow-up based on patient-reported outcomes in patients with chronic kidney disease: A qualitative study of patient perspectives. PLoS One. 2023;18(2):e0281393.

63. Grove B, Ivarsen P, De Thurah A, Kyte D, Henrik HN. Tele follow-up using patient-reported outcomes (PRO) measures in patients with chronic kidney disease-the PRO-KID study: A study protocol for a non-inferiority randomised controlled trial in Denmark. Quality of Life Research.27:S143-S4.

64. Flythe JE, Dorough A, Narendra JH, Wingard RL, Dalrymple LS, DeWalt DA. Development and content validity of a hemodialysis symptom patient-reported outcome measure. Qual Life Res. 2019;28(1):253-65.

65. Pittman ZC, John SG, McIntyre CW. Collection of daily patient reported outcomes is feasible and demonstrates differential patient experience in chronic kidney disease. Hemodial Int. 2017;21(2):265-73.

66. Schick-Makaroff K, Wozniak LA, Short H, Davison SN, Klarenbach S, Buzinski R, et al. Burden of mental health symptoms and perceptions of their management in in-centre hemodialysis care: a mixed methods study. J Patient Rep Outcomes. 2021;5(1):111.

67. Davison SN, Klarenbach S, Manns B, Schnick-Makaroff K, Buzinski R, Corradetti B, et al. Patient-reported outcome measures in the care of in-centre hemodialysis patients. Journal of Patient-Reported Outcomes. 2021;5(1):1-8.

68. Schick-Makaroff K, Wozniak LA, Short H, Davison SN, Klarenbach S, Buzinski R, et al. How the Routine Use of Patient-Reported Outcome Measures for Hemodialysis Care Influences Patient-Clinician Communication: A Mixed-Methods Study. Clin J Am Soc Nephrol. 2022;17(11):1631-45.

For peer review only

For peer review only - http://bmjopen.bmj.com/site/about/guidelines.xhtml

69. Thilly N, Chanliau J, Frimat L, Combe C, Merville P, Chauveau P, et al. Cost-effectiveness of home telemonitoring in chronic kidney disease patients at different stages by a pragmatic randomized controlled trial (eNephro): rationale and study design. BMC Nephrology. 2017;18(1):126.
70. van der Willik EM, Hemmelder MH, Bart HAJ, van Ittersum FJ, Hoogendijk-van den Akker JM, Bos WJW, et al. Routinely measuring symptom burden and health-related quality of life in dialysis patients: first results from the Dutch registry of patient-reported outcome measures. Clin Kidney J. 2021;14(6):1535-44.
71. van der Willik EM, Meuleman Y, Prantl K, van Rijn G, Bos WJW, van Ittersum FJ, et al. Patient-reported outcome measures: selection of a valid questionnaire for routine symptom assessment in patients with advanced chronic kidney disease - a four-phase mixed methods study. BMC Nephrol. 2019;20(1):344.
72. van der Willik EM, Milders J, Bart JAJ, Bos WJW, van Ittersum FJ, Ten Dam M, et al. Discussing results of patient-reported outcome measures (PROMs) between patients and healthcare professionals in routine dialysis care: a qualitative study. BMJ Open. 2022;12(11):e067044.
73. Schick-Makaroff K, Tate K, Molzahn A. Use of Electronic Patient Reported Outcomes in Clinical Nephrology Practice: A Qualitative Pilot Study. Canadian Journal of Kidney Health & Disease. 2019;6:2054358119879451.
74. Snyder C W, A.W., eds. Users' Guide to Integrating Patient-Reported Outcomes in Electronic Health Records Baltimore, MD: John Hopkins University; 2017 [Available from: https://www.pcori.org/document/users-guide-integrating-patient-reported-outcomes-electronic-health-records.
75. Aaronsen N ET, Greenhalgh J, Halyard M, Hess R, Miller D, Reeve B, Santana M, Snyder C. User's Guide to Implementing Patient-Reported Outcomes Assessment in Clinical Practice: International Society of Quality of Life Research; 2015 [Jan 2015]. Available from: https://www.isoqol.org/wp-content/uploads/2019/09/2015UsersGuide-Version2.pdf.
76. Zbrozek A, Hebert J, Gogates G, Thorell R, Dell C, Molsen E, et al. Validation of electronic systems to collect patient-reported outcome (PRO) data-recommendations for clinical trial teams: report of the ISPOR ePRO systems validation good research practices task force. Value Health. 2013;16(4):480-9.
77. Flythe JE, Karlsson N, Sundgren A, Cordero P, Grandinetti A, Cremisi H, et al. Development of a preliminary conceptual model of the patient experience of chronic kidney disease: a targeted literature review and analysis. BMC Nephrology. 2021;22(1):233.
78. Dad T, Tighiouart H, Fenton JJ, Lacson E, Jr., Meyer KB, Miskulin DC, et al. Evaluation of non-response to the In-Center Hemodialysis Consumer Assessment of Healthcare Providers and Systems (ICH CAHPS) survey. BMC Health Services Research.18(1):790.
79. Cohen DE, Lee A, Sibbel S, Benner D, Brunelli SM, Tentori F. Use of the KDQOL-36™ for assessment of health-related quality of life among dialysis patients in the United States. BMC Nephrology. 2019;20(1):112.
80. Nimmo A, Bell S, Brunton C, Campbell J, Doyle A, MacKinnon B, et al. Collection and determinants of patient reported outcome measures in haemodialysis patients in Scotland. QJM. 2018;111(1):15-21.

For peer review only - http://bmjopen.bmj.com/site/about/guidelines.xhtml

81. Calvert MJ, Cruz Rivera S, Retzer A, Hughes SE, Campbell L, Molony-Oates B, et al. Patient reported outcome assessment must be inclusive and equitable. Nature Medicine. 2022;28:1120-4.

82. Absolom K, Holch P, Woroncow B, Wright EP, Velikova G. Beyond lip service and box ticking: how effective patient engagement is integral to the development and delivery of patient-reported outcomes. Qual Life Res. 2015;24(5):1077-85.

83. Albers EAC, Fraterman I, Walraven I, Wilthagen E, Schagen SB, van der Ploeg IM, et al. Visualization formats of patient-reported outcome measures in clinical practice: a systematic review about preferences and interpretation accuracy. J Patient Rep Outcomes. 2022;6(1):18.

84. Snyder C, Smith K, Holzner B, Rivera YM, Bantug E, Brundage M, et al. Making a picture worth a thousand numbers: recommendations for graphically displaying patient-reported outcomes data. Qual Life Res. 2019;28(2):345-56.

85. Grossman LV, Feiner SK, Mitchell EG, Masterson Creber RM. Leveraging Patient-Reported Outcomes Using Data Visualization. Appl Clin Inform. 2018;9(3):565-75.

86. Guha C, Lopez-Vargas P, Ju A, Gutman T, Scholes-Robertson NJ, Baumgart A, et al. Patient needs and priorities for patient navigator programmes in chronic kidney disease: a workshop report. BMJ Open. 2020;10(11):e040617.

87. Zhang R, Burgess ER, Reddy MC, Rothrock NE, Bhatt S, Rasmussen LV, et al. Provider perspectives on the integration of patient-reported outcomes in an electronic health record. JAMIA Open. 2019;2(1):73-80.

88. Schick-Makaroff K, Levay A, Thompson S, Flynn R, Sawatzky R, Thummapol O, et al. An Evidence-Based Theory About PRO Use in Kidney Care: A Realist Synthesis. The Patient. 2021;15(1):21-38.

89. Gibbons C, Porter I, Goncalves-Bradley DC, Stoilov S, Ricci-Cabello I, Tsangaris E, et al. Routine provision of feedback from patient-reported outcome measurements to healthcare providers and patients in clinical practice. Cochrane Database Syst Rev. 2021;10:CD011589.

90. Tong A, Winkelmayer WC, Wheeler DC, Biesen WV, Tugwell P, Manns B, et al. Nephrologists' perspectives on defining and applying patient-centered outcomes in hemodialysis. Clinical Journal of the American Society of Nephrology. 2017;12(3):454-66.

91. Basch E BL, Kerrigan C L, Velikova G. Implementation of Patient Reported Outcomes in routine medical care. American Society of Clinical Oncology Educational Book. 2018;38:122-34.

92. Rabin BA, Brownson RC, Haire-Joshu D, Kreuter MW, Weaver NL. A glossary for dissemination and implementation research in health. Journal of public health management and practice : JPHMP. 2008;14 2:117-23.

93. Stover AM, Haverman L, van Oers HA, Greenhalgh J, Potter CM, Group IPPiCPISW. Using an implementation science approach to implement and evaluate patient-reported outcome measures (PROM) initiatives in routine care settings. Qual Life Res. 2020;30(11):3015-33.

94. Honeyman M MD, Evans H, Davies A. Digital technology and health inequalities: a scoping review (2020) Cardiff: Public Health Wales NHS Trust; 2020 [Available from: https://phw.nhs.wales/publications/publications1/digital-technology-and-health-inequalities-a-scoping-review/?dm_i=21A8,75FES,2H1914,SZM5X,1.

For peer review only - http://bmjopen.bmj.com/site/about/guidelines.xhtml

95. Chapman R, Haroon S, Simms-Williams N, Bhala N, Miah F, Nirantharakumar K, et al. Socioeconomic deprivation, age and language are barriers to accessing personal health records: a cross-sectional study of a large hospital-based personal health record system. BMJ Open. 2022;12(1): e054655.

96. Flythe JE. Integrating PROMs in Routine Dialysis Care: The Devil Is in the (Implementation) Details. Clin J Am Soc Nephrol. 2022;17(11):1580-2.

97. Vasileiou K, Barnett J, Thorpe S, Young T. Characterising and justifying sample size sufficiency in interview-based studies: systematic analysis of qualitative health research over a 15-year period. BMC Med Res Methodol. 2018;18(1):148.

98. Malterud K, Siersma VD, Guassora AD. Sample Size in Qualitative Interview Studies: Guided by Information Power. Qual Health Res. 2016;26(13):1753-60.

99. Bass M, Morris, S., Neapolitan, R. Utilizing Multidimensional Computer Adaptive Testing to Mitigate Burden With Patient Reported Outcomes. AMIA Annual Symposium proceedings AMIA Symposium,. 2015:320-8.

100. Institute of Applied Health Research UoB. RCAT-Renal Computerised Adaptive Test Birmingham: University of Birmingham; 2022 [Available from: https://tinyurl.com/13vdwh0m.

For peer review only - http://bmjopen.bmj.com/site/about/guidelines.xhtml

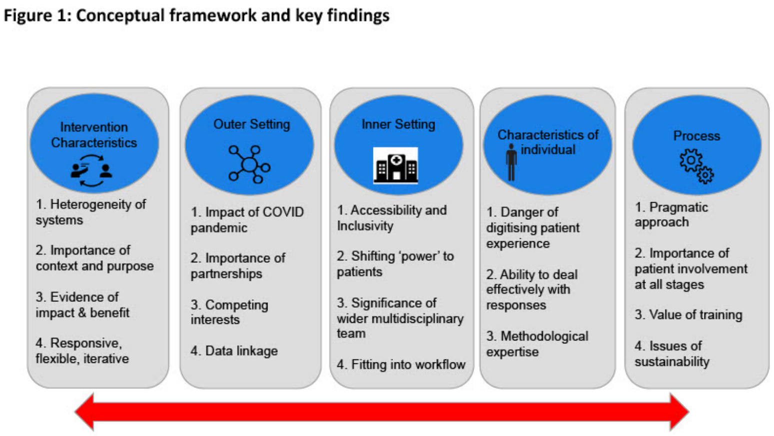

Figure 1: Conceptual framework and key findings

361x256mm (72 x 72 DPI)

Supplementary file S1

Nephrology ePROs search strategy and PRISM ScR diagram

MEDLINE (R) (Ovid) 1946 to 15/12/21

Note: Sets 1 to 6 are based on the Oxford PROM Filter (1) and sets 9-11 and 13 were developed for systematic review exploring measurement properties of PROs used in CKD (2)

1. (HR-PRO or HRPRO or HRQL or HRQoL or QL or QoL).ti,ab.

2. quality of life.mp.

3. (health index* or health indices or health profile*).ti,ab.

4. health status.mp.

5. ((patient or self or child or parent or carer or proxy) adj (appraisal* or appraised or report or reported or reporting or rated or rating or based or assessed or assessment*)).ti,ab.

6. ((disability or function or functional or functions or subjective or utility or utilities or wellbeing or well being) adj2 (index or indices or instrument or instruments or measure or measures or questionnaire* or profile or profiles or scale or scales or score or scores or status or survey or surveys)).ti,ab.

7. ((((patient adj reported adj outcome adj measure*) or patient) adj reported adj outcome*) or capability or capabilities).mp. [mp=title, abstract, original title, name of substance word, subject heading word, keyword heading word, protocol supplementary concept word, rare disease supplementary concept word, unique identifier]

8. 1 or 2 or 3 or 4 or 5 or 6 or 7

9. (Renal replacement therapy or APD or Automated Peritoneal Dialysis or CAPD, Continuous Ambulatory Peritoneal Dialysis or CCPD or Continuous cyclic peritoneal dialysis or dialysis or h*emofiltration or h*emodiafiltration or h*emodialysis or kidney transplant* or predialysis or renal replacement or renal transplant*).mp.

10. (CRF or chronic renal failure or CKF or chronic kidney failure or kidney disease* or renal disease or kidney failure or renal failure or CKD or chronic kidney disease or ESKD or end stage kidney disease or ESKF or end stage kidney failure or ESRF or end stage renal failure or ESRD or end stage renal disease or kidney insufficiency).mp.

11. Renal Insufficiency, Chronic/

12. 9 or 10 or 11

For peer review only

13. (PRO integration or Clinical PRO application* or telePRO or automated PRO algorithm* or screening purpose* or PRO questionnaire* or Patient-reported outcome questionnaire* or Patient-reported symptom* or Patient-centred care or Patient self-report* or Self-report health or Self-rated health or Self-reported measure* of health or Health outcome* or Health communication* or Hospital performance evaluation* or Automated telephone survey system* or paper-based survey* or web-based survey* or web-based PRO platform* or web-based system* or PRO collection* or PRO measure* or PRO intervention* or PRO assessment intervention* or PRO data or PRO assessment* or Routine PRO assessment* or Routine PRO collection or Symptom assessment* or Symptom monitoring or Symptom data or Functional status or Electronic PRO assessment* or Electronic PRO system* or ePRO orePRO* or ePRO system* or PRO system* or Generic PRO system* or PRO-based clinical alert system*).mp.

14. 8 and 12 and 13

For peer review only

EMBASE (Ovid) 1974 to 15/12/21

Note: Sets 1-6 based on search strategy developed for systematic review exploring measurement properties of PROs used in CKD (2).

1. (Renal replacement therapy or APD or Automated Peritoneal Dialysis or CAPD, Continuous Ambulatory Peritoneal Dialysis or CCPD or Continuous cyclic peritoneal dialysis or dialysis or h*emofiltration or h*emodiafiltration or h*emodialysis or kidney transplant* or predialysis or renal replacement or renal transplant*).mp.

2. (CRF or chronic renal failure or CKF or chronic kidney failure or kidney disease* or renal disease or kidney failure or renal failure or CKD or chronic kidney disease or ESKD or end stage kidney disease or ESKF or end stage kidney failure or ESRF or end stage renal failure or ESRD or end stage renal disease or kidney insufficiency).mp.

3. (PRO integration or Clinical PRO application* or telePRO or automated PRO algorithm* or screening purpose* or PRO questionnaire* or Patient-reported outcome questionnaire* or Patient-reported symptom* or Patient-centred care or Patient self-report* or Self-report health or Self-rated health or Self-reported measure* of health or Health outcome* or Health communication* or Hospital performance evaluation* or Automated telephone survey system* or paper-based survey* or web-based survey* or web-based PRO platform* or web-based system* or PRO collection* or PRO measure* or PRO intervention* or PRO assessment intervention* or PRO data or PRO assessment* or Routine PRO assessment* or Routine PRO collection or Symptom assessment* or Symptom monitoring or Symptom data or Functional status or Electronic PRO assessment* or Electronic PRO system* or ePRO or ePRO* or ePRO system* or PRO system* or Generic PRO system* or PRO-based clinical alert system*).mp.

4. *patient/ or *outcome assessment/ or *questionnaire/ or *"quality of life"/ or *self report/

5. 1 or 2

6. 3 and 4 and 5

For peer review only

PsycINFO (Ovid) 1967 to 15/12/21

Note: Sets 1 to 6 are based on the Oxford PROM Filter (1). Sets 7, 9-11 and 13 based on search strategy developed for systematic review exploring measurement properties of PROs used in CKD (2)

1.(HR-PRO or HRPRO or HRQL or HRQoL or QL or QoL).ti,ab.

2.quality of life.mp. 103501

3.(health index* or health indices or health profile*).ti,ab.

4.health status.mp.

5.((patient or self or child or parent or carer or proxy) adj (appraisal* or appraised or report or reported or reporting or rated or rating or based or assessed or assessment*)).ti,ab.

6.((disability or function or functional or functions or subjective or utility or utilities or wellbeing or well being) adj2 (index or indices or instrument or instruments or measure or measures or questionnaire* or profile or profiles or scale or scales or score or scores or status or survey or surveys)).ti,ab.

7.((((patient adj reported adj outcome adj measure*) or patient) adj reported adj outcome*) or capability or capabilities).mp. [mp=title, abstract, heading word, table of contents, key concepts, original title, tests & measures, mesh word]

8.1 or 2 or 3 or 4 or 5 or 6 or 7

9.(Renal replacement therapy or APD or Automated Peritoneal Dialysis or CAPD, Continuous Ambulatory Peritoneal Dialysis or CCPD or Continuous cyclic peritoneal dialysis or dialysis or h*emofiltration or h*emodiafiltration or h*emodialysis or kidney transplant* or predialysis or renal replacement or renal transplant*).mp.

10.(CRF or chronic renal failure or CKF or chronic kidney failure or kidney disease* or renal disease or kidney failure or renal failure or CKD or chronic kidney disease or ESKD or end stage kidney disease or ESKF or end stage kidney failure or ESRF or end stage renal failure or ESRD or end stage renal disease or kidney insufficiency).mp.

11.*Organ Transplantation/ or *Hemodialysis/ or *Kidneys/ or *Kidney Diseases/ or Renal Insufficiency, Chronic.mp.

12.9 or 10 or 11

13.(PRO integration or Clinical PRO application* or telePRO or automated PRO algorithm* or screening purpose* or PRO questionnaire* or Patient-reported outcome questionnaire* or Patient-reported symptom* or Patient-centred care or Patient self-report* or Self-report health or Self-rated health or Self-reported measure* of health or Health outcome* or Health communication* or Hospital performance evaluation* or Automated telephone survey system* or paper-based survey* or web-based survey* or web-based PRO platform* or web-based system* or PRO collection* or PRO measure* or PRO intervention* or PRO

For peer review only

assessment intervention* or PRO data or PRO assessment* or Routine PRO assessment* or Routine PRO collection or Symptom assessment* or Symptom monitoring or Symptom data or Functional status or Electronic PRO assessment* or Electronic PRO system* or ePRO or ePRO* or ePRO system* or PRO system* or Generic PRO system* or PRO-based clinical alert system*).mp.

14.8 and 12 and 13

For peer review only

CINAHL (via EBSCO host) inception to 15/12/21

Sets 1 and 2 based on search strategy developed for systematic review exploring measurement properties of PROs used in CKD (2)

S1. (MH "Kidney Failure, Chronic/TH/TM/TD/SU/SS/RF/RH/PF/PR/PC/PP/PA/NU/MO/ME/ET/EI/EP/ED/EC/DT/DI/CO/CL") OR "((Renal replacement therapy or APD or Automated Peritoneal Dialysis or CAPD, Continuous Ambulatory Peritoneal Dialysis or CCPD or Continuous cyclic peritoneal dialysis or dialysis or h*emofiltration or h*emodiafiltration or h*emodialysis or kidney transplant* or predialysis or renal replacement or renal transplant*).mp.) OR ((CRF or chronic renal failure or CKF or chronic kidney failure or kidney disease* or renal disease or kidney failure or renal failure or CKD or chronic kidney disease or ESKD or end stage kidney disease or ESKF or end stage kidney failure or ESRF or end stage renal failure or ESRD or end stage renal disease or kidney insufficiency).mp.) OR (*Organ Transplantation/ or *Hemodialysis/ or *Kidneys/ or *Kidney Diseases/ or Renal Insufficiency, Chronic.mp.)"

S2. ((PRO integration or Clinical PRO application* or telePRO or automated PRO algorithm* or screening purpose* or PRO questionnaire* or Patient-reported outcome questionnaire* or Patient-reported symptom* or Patient-centred care or Patient self-report* or Self-report health or Self-rated health or Self-reported measure* of health or Health outcome* or Health communication* or Hospital performance evaluation* or Automated telephone survey system* or paper-based survey* or web-based survey* or web-based PRO platform* or web-based system* or PRO collection* or PRO measure* or PRO intervention* or PRO assessment intervention* or PRO data or PRO assessment* or Routine PRO assessment* or Routine PRO collection or Symptom assessment* or Symptom monitoring or Symptom data or Functional status or Electronic PRO assessment* or Electronic PRO system* or ePRO or ePRO* or ePRO system* or PRO system* or Generic PRO system* or PRO-based clinical alert system*).mp.)""

S3. S1 and S2

References

(1) PROM Group, A.M., Carolina Casañas i Comabella, Monica Hadi, Elizabeth Gibbons, Ray Fitzpatrick, Nia Roberts, *PROM GROUP CONSTRUCT & INSTRUMENT TYPE FILTERS.* 2010.

(2) Aiyegbusi, O. L., Kyte, D., Cockwell, P., Marshall, T., Gheorghe, A., Keeley, T., Slade, A., & Calvert, M. (2017). Measurement properties of patient-reported outcome measures (PROMs) used in adult patients with chronic kidney disease: A systematic review. *PloS one*, *12*(6), e0179733. https://doi.org/10.1371/journal.pone.0179733

For peer review only

PRISMA diagram

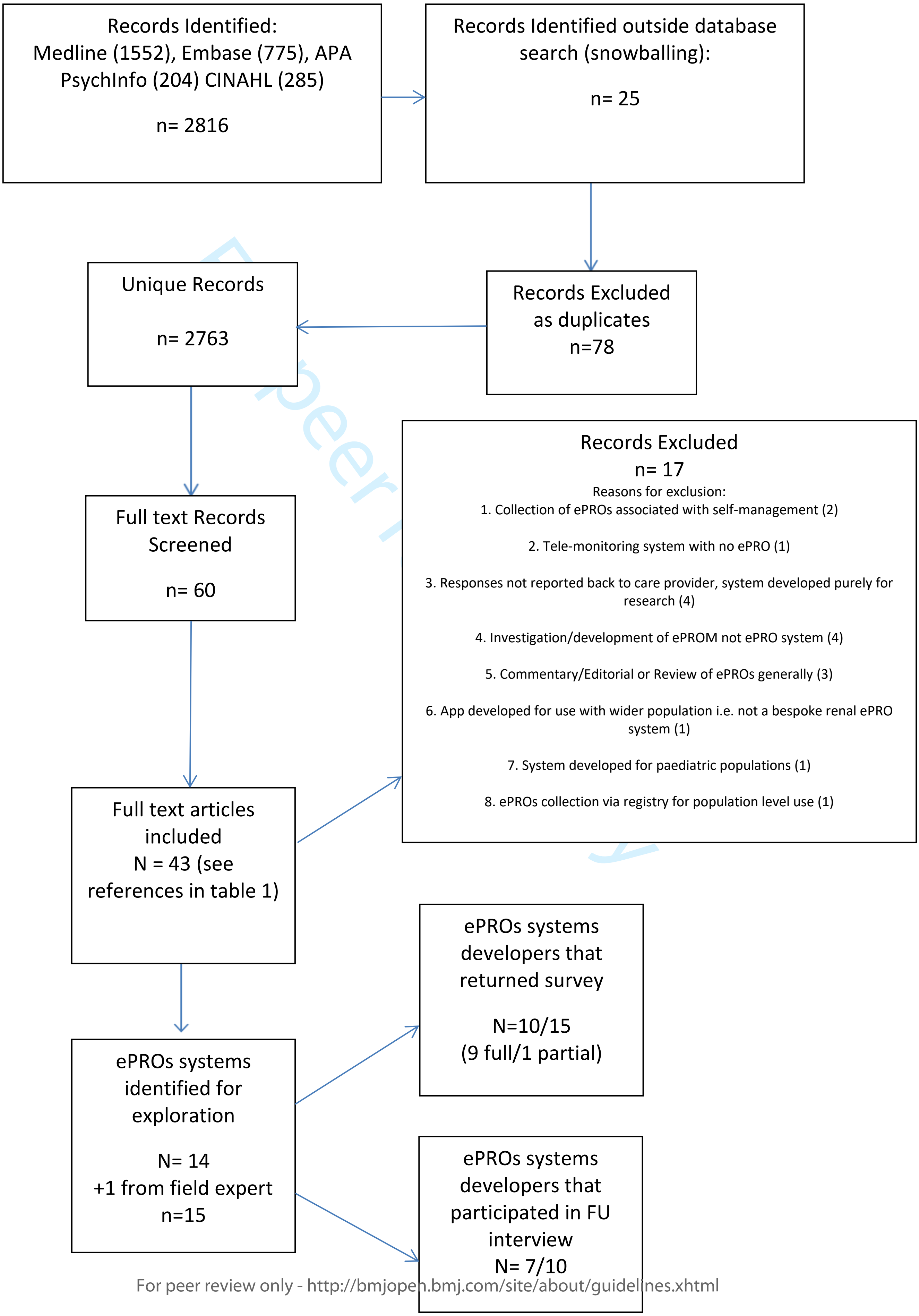

For peer review only - http://bmjopen.bmj.com/site/about/guidelines.xhtml

Electronic Patient-Reported Outcomes (ePRO) systems used in nephrology settings

1. Electronic Patient-Reported Outcomes (ePRO) Systems used in Nephrology settings

Consent Form.

Please note you may choose to save and continue your survey response at a later time. If you choose this option at the end of each page, you will be directed to enter your email address and we will email you a link for you to access the survey in the future.

1. I confirm that I have read and understood the information in the Participant information sheet version 3.0 dated 06.06.2021 and understand what is expected of me. *

2. I confirm that I have been given the opportunity to ask questions regarding the study, and if asked, the questions were answered to my full satisfaction. *

3. I understand that my participation is voluntary and that I am free to withdraw at any time, but within 5 working days following survey completion (before my data is integrated into the dataset), without giving any reason. *

4. I understand that any personal information will be anonymized but institutional or system identifiers will be used in research outputs i.e. publications/presentations. *

5. I understand that the information collected may be used to support other research in the future and may be shared anonymously with other researchers. *

6. I understand that my personal data will be processed for the purposes detailed above, in accordance with the General Data Protection Regulation and Data Protection Act 2018 *

7. I agree to take part in the survey (please note, if you answer no to this question you will be taken to the end of this survey) *

8. OPTIONAL I am willing to take part in a follow up interview following survey completion, where required.
I understand:

The interview will be audio-recorded and that the transcript of the recording will be shared with researchers working on the above study at the University of Birmingham.

My participation in this interview is voluntary and that I am free to withdraw at any time, up to 10 working days following interview (before my data is integrated into the dataset), without giving any reason.

I will have the opportunity to review any interview transcript and redact any proprietary information before analysis.

I must return reviewed transcripts within 10 working days, otherwise it will be assumed I am happy with the contents.

*

9. Thank you very much for offering to take part in the the optional interview. Please enter your preferred contact details below and a member of the team will be in contact to arrange a convenient date and time. *

First name *

Last name *

Email *

3. ePRO System Characteristics Survey

10. Please enter your name (this information is for administrative purposes only and your data will be pseudonymised) *

11. What was your job title during system development?

12. What is your system called?

Name

Version number

13. Please describe your key responsibilities in the ePRO system development/implementation, please include clinical and non-clinical responsibilities as appropriate.

14. Please state the purpose of your ePRO system (please tick those that apply, there may be more than one primary purpose)

	Primary objective of system	Secondary objective of system	Not a system objective
Improving symptom management (associated with underlying chronic kidney disease)	☐	☐	☐
Improving symptom management (associated with treatment monitoring e.g. Haemodialysis)	☐	☐	☐
Identification of psychosocial problems	☐	☐	☐
To facilitate patient-provider communication	☐	☐	☐
Research	☐	☐	☐
Benchmarking of services	☐	☐	☐
Clinical service commissioning	☐	☐	☐
Support transition of care e.g. to renal replacement therapy or conservative care	☐	☐	☐

If your ePRO system has another purpose, please state

15. What is the population focus of the system (please tick all that apply)

- ☐ Chronic Kidney Disease Stages 1-3
- ☐ Chronic Kidney Disease Stages 4-5 pre dialysis
- ☐ In-centre Haemodialysis
- ☐ Home Haemodialysis
- ☐ Peritoneal Dialysis
- ☐ Transplant
- ☐ Conservative Care

16. When was ePRO system launched?

DD/MM/YYYY

17. How was the system developed?

- ☐ Informatics team within the host organisation (single organisation)
- ☐ Collaboration across two or more organisations
- ☐ Already available Commercial product

Comments:

18. Approximate number of patient users

19. Approximate number of clinician users

20. Primary location of use (please tick all that apply)

- ☐ Primary Care (Community/General Practice clinic)
- ☐ Secondary Care (Hospital clinic)
- ☐ Dialysis Centre
- ☐ Home

21. What PRO Measure (s) are you using?

	PRO Measure
Are you using a new measure?	☐
Are you using an existing measure(s)?	☐
Do you need to use a licence for use?	☐

Comments: Please state name of PRO measure(s)

22. Has the PRO Measure been validated with your patient population? *

- ☐ Yes
- ☐ No

23. How was your ePRO system validated?

- ☐ Formal paper-electronic equivalence testing
- ☐ None
- ☐ Other (please specify):

24. What analysis metric is being used to make or inform clinical decisions?

- ☐ Change from baseline
- ☐ Final value
- ☐ Time to event
- ☐ Other (please specify):

25. How was your ePRO system funded? (you may tick more than one option if mixed model funding used)

- ☐ Commercial
- ☐ Academic
- ☐ Charitable
- ☐ Governmental

Other: Please state

26. What security features does your ePRO system employ? (please tick all that apply)

- ☐ Secure log in (password)
- ☐ Encryption
- ☐ Other (please specify):

6. Administration of PRO assessments/level of automation

27. Is your ePRO system web-based and/or App based (please tick all that apply)

- ☐ Non-responsive website i.e. for display on desk top computer
- ☐ Responsive/mobile website i.e. content displays and function across all devices
- ☐ Mobile App

28. How is the ePRO system accessed? (please tick all that apply)

- ☐ Computer
- ☐ Tablet
- ☐ Interactive Voice Response System
- ☐ Clinic based kiosk
- ☐ Smartphone
- ☐ Other (please specify):

29. Timing of ePRO assessment

- ☐ Prior to clinical assessment
- ☐ Set timepoint weekly
- ☐ Set timepoint monthly
- ☐ Set timepoint 3 monthly
- ☐ Set timepoint 6 monthly
- ☐ Set timepoint annually
- ☐ As required (by patient)
- ☐ Other (please specify):

30. PRO assessment selection: How does your ePRO system select which PRO to use?

- [ ] Automatic (by system) i.e. Provider
- [ ] Patient

Comments:

31. Question Format: How does the ePRO system present questions?

- [ ] One question per page
- [ ] Multiple questions per page
- [ ] Mixed format of single and multiple questions

32. Question advancement: how does the ePRO system move from one question to the next?

- [ ] Mouse click
- [ ] Automatic on completion

33. What page features are available? (please tick all that apply)

- [ ] Progress Bar
- [ ] Visual graphics i.e. graph, diagram, chart
- [ ] Other (please specify):

34. How does your ePRO system avoid missing data during completion? (Please tick all that apply)

- [ ] Allows multiple logins per assessment with automatic save function
- [ ] Allows multiple logins per assessment with save and return later function
- [ ] Allows not applicable (N/A) response
- [ ] Default response pre-selected (pre-populated neutral response)
- [ ] Reminders
- [ ] Other (please specify):

35. Is the ePRO available in more than one language? *

	Yes	No
Is the ePRO available in more than one language?	☐	☐

Comments: Please state available languages

7. Cross cultural validation

36. Has the ePRO measure been validated for use in multiple languages?

- ☐ Yes
- ☐ No

Comments: if yes, please specify languages

37. How does the ePRO system notify that assessments have been completed? (Please tick all that apply)

- ☐ Automated submission notification to patient
- ☐ Automated submission notification to clinicians
- ☐ Email notification to patient from clinical team following review of responses
- ☐ App based notification
- ☐ Other (please specify):

38. How are patients reminded to complete their ePRO assessment? (Please tick all that apply)

- ☐ Email
- ☐ Telephone call
- ☐ Text message: SMS
- ☐ Verbally
- ☐ By letter
- ☐ No reminder
- ☐ App based push notification

39. Does the ePRO system offer flexible system features (please tick all that apply)

- ☐ Web based home and clinic login access
- ☐ Multiple assessment scheduling options
- ☐ Two or more sources for PRO selection (patient/provider)

- [ ] Self identification of important issues by patient (computer adaptive testing functionality)
- [ ] Free text availability
- [ ] Multiple language availability
- [ ] In app or push notifications
- [ ] Other (please specify):

9. ePRO System integration and reporting

40. Please outline the ePRO report content (Please tick all that apply)

- [ ] Current scores including summary and individual scores
- [ ] Longitudinal change
- [ ] Interpretation included in report
- [ ] Cut scores (e.g. low, medium, high)
- [ ] Population norms or reference values
- [ ] Identification of meaningful change
- [ ] Modifiable reports
- [ ] General guidelines
- [ ] Other (please specify):

41. How are ePRO reports accessed? (Please tick all that apply)

- [ ] Via clinical portal/electronic patient health record
- [ ] Via immediate access i.e. summary print out on completion
- [ ] Results restricted to clinical encounter
- [ ] Other (please specify):

42. Who has access to ePRO results/summary reports (please tick all that apply)

- [ ] Patient
- [ ] Clinicians
- [ ] Care Provider
- [ ] Multiple care provider access
- [ ] Other (please specify):

43. Who is responsible for initial review and action of ePRO results?

- [ ] Medical staff
- [ ] Nursing staff
- [ ] Administrative staff
- [ ] Other member of multidisciplinary team

Comments:

44. What form does the clinical response to ePRO report take? (please tick all that apply)

- ☐ Prescribed electronic response dependent on score
- ☐ Clinician/staff follow up; follow up type (face to face, virtual) dependent on score/decision aids
- ☐ Automatic referral to member of multidisciplinary team
- ☐ Automated patient education/message regardless of score
- ☐ Other (please specify):

45. Is there visual presentation of the ePRO scores? (please tick all that apply)

- ☐ Graphical
- ☐ Tabular
- ☐ Numerical
- ☐ Emoticon
- ☐ Colour coded
- ☐ Other (please specify):

46. Does your ePRO system have a safety alert system, dependent on respondent scores? *

- ☐ Yes
- ☐ No

10. Management of safety

47. Management of safety: Who is the intended primary recipient and audience of the ePRO score alert and associated response?

- ☐ Clinician/Staff
- ☐ Patients
- ☐ Care provider
- ☐ Caregiver
- ☐ Multiple recipients i.e. clinicians and patients

Comments:

48. Management of safety: what form do ePRO score alerts take? (please tick all that apply)

- ☐ Email
- ☐ Text message/SMS
- ☐ Telephone call
- ☐ Verbal
- ☐ Real time alert
- ☐ In App notification
- ☐ None

11. Training

49. Do you offer training on the ePRO system? *

- ☐ Yes
- ☐ No

12. Training

50. Who receives training on the ePRO system? (Please tick all that apply)

- ☐ Clinical staff
- ☐ Administrative staff
- ☐ Patients
- ☐ Carers
- ☐ Training available but not specified
- ☐ No formal training available

51. What form does ePRO system training take? (Please tick all that apply)

- ☐ Face to face
- ☐ Online
- ☐ Integrated into ePRO system
- ☐ No formal training

52. Is there a facility for patient education through the ePRO system? (please tick all that may apply)

- [ ] Patient education administered within system
- [ ] Education linked to ePRO scores
- [ ] Automatic documentation of actions
- [ ] No patient education offered

Comments:

13. Evaluation

53. Has your ePRO system been evaluated? *

14. Evaluation

54. What form of evaluation did you undertake?

- [ ] Formative research
- [ ] Pre implementation feasibility testing
- [ ] Progress focused evaluation concurrent to implementation
- [ ] Retrospective evaluation

Comments:

55. Did you include patients in your evaluation?

56. What, if any, changes did you make following the evaluation process

15. Additional features

57. Is there linkage between the ePRO system and other electronic data systems? (Please tick all that apply)

- ☐ Patient Electronic Health Record
- ☐ Appointment/scheduling systems
- ☐ Patient Billing systems
- ☐ No linkage
- ☐ Other (please specify):

58. Accessibility: is the data available to areas outside nephrology settings?

- ☐ Yes
- ☐ No

Comments: If yes, please state

59. Does your ePRO system offer any additional features (please tick all that apply)

- ☐ Quality of care evaluation
- ☐ Patient satisfaction data collected
- ☐ Accreditation reporting
- ☐ None
- ☐ Other (please specify):

60. Is IT support available for your ePRO system?

- ☐ Full
- ☐ Limited
- ☐ User Manual available
- ☐ None

Comments: if Limited, please state what support is offered

16. Contact details

61. Would you like a summary of the findings of this survey?

- ☐ Yes
- ☐ No

If yes, please enter your preferred contact details below

Thank you very much for taking part in this survey.
Would you like to get in touch with the research team?
If you would like any more information, please contact Nicola Anderson at NEA451@student.bham.ac.uk

Nephrology ePRO systems review - Topic Guide

Introduction/Ice breaker

The introduction will set the tone and start building rapport:
Introduce self as a Nurse Researcher with a clinical background specialising in nephrology research and that this interview is being undertaken as part of a clinician doctoral research fellowship funded by the National Institute for Health Research.

Tell respondents the general purpose of the interview

Before the interview, check that the respondents are still happy to take part and have signed the study specific electronic consent form.

Participants will be reminded that all individual self-identifiers will be removed before transcripts are analysed but that the System/institution itself will be named.

1. Icebreaker question: Please outline your role and key responsibilities as a System Lead for the ePRO outlined in your survey responses

2. Can you describe how the ePRO is being implemented in your institution?
   Prompts: What was/is your implementation plan? What stage are you at?

3. What is the purpose of assessment?
   Prompt: To inform clinical care, identify symptoms/tailor care, audit, benchmark, real-world evidence, trials Mixture?

4. Did you use internal/external change agents to help implementation?
   Did you use peer support/champions to help implementation?
   Prompt: Did you get endorsement or support from leaders? If yes, what form did it take, if no, why do you think not?

5. Did you use an existing PROM? Or develop a new measure?
   Prompt: Did you measure the psychometric properties of any existing measure? If a new measure was created – what methodological approach was taken? Use of core outcome sets? COSMIN assessment? Level of patient input? FDA guidance?

6. Who are the key influential stakeholders for the ePRO? What was your engagement strategy to get these people on board?
   Prompts: Stakeholders could be Health care professionals, patients, carers, senior managers, administration staff. Were these individuals involved in c0-designing the system – in particular were patients involved?

7. What is the general level of receptivity in your organization?
   Prompt: Are users (patients/Healthcare Professionals) happy to use the ePRO,

For peer review only - http://bmjopen.bmj.com/site/about/guidelines.xhtml

are they confident? If not, do you know why not?

8. Were any supports developed to help you implement and use the ePRO?
Prompts: Training resources? Marketing material?> toolkit? Were these resources available digitally, was face to face training made available.

9. What is the priority for getting the ePRO implemented relative to other initiatives that are happening now?
Prompt: for example was it affected by the COVID 19 pandemic?

10. Was the ePRO piloted prior to full-scale implementation?
Prompt: Can you describe the context of the pilot? What worked well? What did not work well?

11. Was the ePRO available in more than one language? Were there mechanisms to allow use by as many patients as possible?
Prompt: were any translations culturally validated?

12. What mechanisms do you have in place to allow maximum accessibility?
Prompt: was the ePRO available for use with patients who have impairments, or can't access IT

13. What direct and indirect costs were incurred to implement the ePRO?
Prompts: Did you need to make changes to infrastructure i.e. IT systems, staffing to support ePRO, adaptions to existing workflow and care delivery systems

14. Did you have sufficient resources to implement the ePRO?
Prompt: if not, what should be prioritised? Links to EPR? Safety features?

15. Do you think the ePRO has improved the way care is being delivered? If yes, how do you think this takes effect?
Prompt: Improved access to services? Help with self-management?, Reduced travel time and expense? To support individual care/tailor care to individual needs, audit, benchmarking, real-world evidence generation – all of these? Other

16. Are you collecting any information to assess impact of the ePRO? If so what type of data?
Prompt: health economic data to assess cost effectiveness, average length of consultation , hospitalisations, death rate, health service interactions, patient and clinician satisfaction

17. What kind of performance measures, policies, regulations, or guidelines influenced the decision to implement the ePRO? Does it provide your organisation with an advantage?
Prompt: Local, state, national measures? Were there incentives to support the

For peer review only - http://bmjopen.bmj.com/site/about/guidelines.xhtml

ePRO

18. Overall has the ePRO been implemented according to the implementation plan?
Prompt: What worked well (Facilitators), what did not (Barriers), what would you change?

19. Are you aware of other ePROs systems in regular/systematic use in nephrology settings?
Prompt: If yes, which systems and what are the associated institution? Do you know who the System Lead is?

20. How does your ePRO compare to other similar/existing ePROs in your setting?
Prompt: Are you aware of any specific advantages/disadvantages of your digital PRO?

21. Do you have anything else to add?

We have now reached the end of the interview. Please be reminded that any personal identifiers will be removed but institutional and system identifiers will be used in any subsequent outputs. You will be given a copy of the transcript after the interview and will have 10 working days to inform the lead researchers of any redactions you wish to make or study withdrawal. Many thanks for your participation.

For peer review only - http://bmjopen.bmj.com/site/about/guidelines.xhtml

Table S1: Overview of ePRO systems with additional data

Name of System	Country<br>No of patient users<br>Population	Primary objective of system<br><br>Main place of completion	In current use<br>Yes/No<br>Timing of assessment	System Measures and summary of measured domains or items
**ANZDATA SWIFT PROMs Module (1-5)**	Australia<br><br>Collection via national registry<br>143 dialysis centres<br>Target n=2400<br><br>In-centre haemodialysis	Improving symptom management associated with CKD and treatment<br>Research<br><br>Dialysis centres	Yes – pilot study reported, Cluster RCT in progress<br>3 monthly for 12 months	**SONG-HD fatigue measure** (6)<br>Three items that assess (1) the effect of fatigue on life participation, (2) tiredness, and (3) level of energy.<br><br>**IPOS-Renal** (7)<br>IPOS-Renal is a short measure , combining the most common symptoms (pain, shortness of breath, weakness, nausea, vomiting, poor appetite, constipation, sore/dry mouth, drowsiness, poor mobility, itching, difficulty sleeping, restless legs, changes in skin, diarrhoea) renal patients experience plus additional items from IPOS on concerns beyond symptoms, such as information needs, practical issues, family anxiety<br><br>**EuroQoL 5-Dimension 5-Level (EQ-5D-5L)** (8)<br>Measures quality of life across 5 domains: mobility, self-care, usual activities, pain/discomfort, and anxiety/depression. Each dimension is scored on a 5-level severity ranking that ranges from "no problems" through "extreme problems."
**cPRO-Collaborate Kidney Care (9)**	United States of America<br>Single renal unit<br><br>n= approximately 350<br>CKD Stages 1-3<br>CKD Stages 4-5 pre dialysis	Improving symptom management associated with CKD, Patient-provider communication<br>Community/general practice/hospital clinic	Yes – clinical demonstration model<br>72 hours prior to clinical assessment visit and 3 monthly FU	**PROMIS and FACIT measures** (9)<br>Items from PROMIS and FACIT to measure: Health perception and global health, fatigue, anxiety, pain, depression, physical function, shortness of breath, urinary frequency, oedema, Appetite, itching, side effect bother, shared decision-making, self-efficacy managing treatments, self-efficacy managing symptoms, treatment satisfaction

**Cambian (ePRO-KIDNEY) (10-16)**	Canada<br>Regional<br><br>ePRO Kidney: n= 543 home dialysis patients<br><br>Home Haemodiaylsis, Peritoneal dialysis	Enhance and support person centred kidney care via research program on ePRO use and implementation<br><br>Secondary Care (Hospital clinic), Dialysis Centre<br>Home	No – platform used within reported research projects<br>Prior to clinical assessment<br>Set time point 3 monthly	**Patient Assessment of Care for Chronic Conditions-20 questionnaire (PACIC-20) (17, 18)**<br>Patient Assessment of Chronic Illness Care (PACIC) assesses the implementation of the chronic care model (CCM) from the patient perspective that focuses on the receipt of patient-centred care and self-management behaviours, this 20 item instrument has five pre-defined domains: patient activation (3 items), delivery system/practice design (3 items), goal setting/tailoring (5 items)<br><br>**Kidney Disease Quality of Life-36 (KDQOL-36) (19, 20)**<br>Based on the KDQOL-SF™. It includes the SF-12 as generic core plus the burden of kidney disease, symptoms/problems of kidney disease, and effects of kidney disease scales from the KDQOL-SF™v1.3.36 items, and two cores: the SF-12 (i.e., generic QOL) and the disease-specific core. The generic core consists of two domains: the physical component summary (PCS) [6 items] and the Mental Component Summary (MCS) [6 items]. The disease-specific core consists of 24 items with 3 domains: symptoms and problems (12 items), burden of kidney disease (4 items) and effects of kidney disease (8 items).<br><br>**Edmonton Symptom Assessment System renal (ESAS-r RENAL) (21)**<br>ESAS-R Renal 10 items (symptoms - pain, tiredness, drowsiness, nausea, lack of appetite, shortness of breath, depression, anxiety, well-being, other problem - Can be modified to include Restless Legs Syndrome single question, RLS defined as 'uncontrollable urge to move your legs')<br><br>**EQ-5D-5L (8)**<br>As above
**OPT-ePRO (22-25)**	United Kingdom<br>Collection via national registry across 3 renal units<br><br>Target n = Approx. 600 potential users	Improving symptom management associated with CKD and treatment, Research	No – pilot study reported<br>Prior to clinical assessment	**POS-S-RENAL (26)**<br>POS-S can also be used on its own, if only a symptom assessment is required, POS-S is available as a renal card - this version assesses 17 symptoms (symptoms (pain, shortness of breath, weakness, nausea, vomiting, poor appetite, constipation, sore/dry mouth, drowsiness, poor mobility, itching, difficulty sleeping, restless legs, changes in skin, diarrhoea, feeling anxious, feeling depressed), plus open fields to list main symptoms

	CKD Stages 1-3, CKD Stages 4-5 pre dialysis, In-centre HD, Home HD, PD Transplant, Conservative Care	HD units and outpatient clinics (Tx, PD, low clearance), home		**EQ-5D-5L (8)**<br>As above
**Penguin (Cievert Ltd)<br>(no published data)**	United Kingdom<br>Single renal unit<br><br>Pilot n=5<br><br>Transplant	Facilitate patient-provider communication<br><br>Secondary care – aim is to complete from home	Yes- Research pilot in progress<br>Prior to clinical assessment visit or annual review	**Check-up questionnaire (copy obtained from developer)**<br>Main concern, pain, breathing, Changes in urine output, swelling, skin/mouth problems, bone/joint problems, fertility (as well as domains on medications/appointments)<br><br>**Annual review (copy obtained from developer)**<br>Main problems, emotional health (Body changes, Mood swings)<br>Physical health (medication, activity, skin heath, smoking & drinking)<br>Sexual health and fertility
**RePROM (27-31)**	United Kingdom<br>Single renal unit<br><br>n=24<br><br>CKD Stages 4-5 pre dialysis	Improving symptom management associated with CKD and treatment<br><br>Secondary care (hospital clinic), home	No – pilot study reported.<br>Monthly reporting	**New study specific measure – RePROM (27)**<br>Items on fatigue; shortness of breath; loss of appetite; nausea or vomiting; itchiness or dry skin; pain; problems with fistula; faintness or dizziness; difficulty sleeping; restless legs; diarrhoea and ankle swelling. The questionnaire<br>also includes an open text item
**Unnamed System 1 (32)**	Canada<br>Single renal unit<br><br>n=approx. 60<br><br>In-centre HD, Transplant	Improving symptom management associated with CKD Research<br><br>Secondary care (hospital clinic), Dialysis centre	No – in development: pre-defined set time-points within research protocol	**PROMIS CATs (33) (unspecified)**<br>Previous pilot study to assess usability collected data from several measures (32)

**'Your symptoms matter' (34, 35)**	Canada<br>1 provincial region<br><br>n=1459<br><br>In-centre HD	Improving symptom management associated with CKD and treatment<br><br>Secondary Care (Hospital clinic), Dialysis Centre	Yes – pilot study reported<br>Every 4-6 weeks	**ESAS-r: Renal (21)**<br>As above
***Survey data only - developers declined participation in optional FU interview**				
**Ambuflex (PRO-KID** Non-inferiority pragmatic randomised controlled trial evaluating incorporating Ambuflex platform where clinicians review responses) **(36-41)**	Denmark<br>**Ambuflex** – National<br>**PRO-KID** – one hospital renal unit<br><br>Ambuflex<br>CKD Stages 4-5 pre dialysis, Conservative Care<br><br>PRO-KID<br>CKD Stages 4-5 pre dialysis	Improving symptom management associated with CKD<br>Patient-provider communication<br><br>**Ambuflex**<br>Home<br><br>**PRO-KID -**<br>home	**Ambuflex** - Yes every 3rd month. PRO and laboratory tests inform clinicians whether the patient needs contact with outpatient clinic<br><br>**(PRO-KID** – trial in progress, every 3 months)	**Ambuflex** 27 item Renal Disease questionnaire (37)L including domains on kidney disease (Lack of appetite, food aversion, feeling of unease, nausea, vomiting, itchy skin, shortness of breath, swollen legs, dizziness, difficulty remembering, difficulty concentrating, restless legs, feeling tired, bodily pain, limited in regular daily activities, constipation, diarrhoea, nocturnal urination, trouble sleeping, worried about future health, difficulties remembering to take medication) and Quality of life (2 items), free text space for 3 topics to would like to discuss<br>**PRO-KID**<br>1. Renal-specific domains, items from • KDQOL-SF • EORTC • SF-GH1 • Need/wish for consultation • Free textbox<br>2. Additional research PROs not available to clinicians administered at baseline, 3, 6 and 18 months (36)
**SMaRRT-HD™ (42, 43)**	United States of America<br>Regional (clinic and dialysis unit)<br><br>N=62<br><br>In-centre HD	Improving symptom management associated with treatment<br><br>Dialysis Centre (Clinic + treatment area)	Unknown – feasibility study reported Twice monthly administered during first 30 minutes of HD (recall period of the last HD treatment for each symptom	**New Study specific measure - SMaRRT-HD™** (42)<br>Symptom Monitoring on Renal Replacement Therapy-Hemodialysis (SMaRRT-HD™) is a 14-item PROM intended for use in hemodialysis patents. SMaRRT-HD™ uses a single treatment recall period and a 5-point Likert scale to assess symptom severity across 13 Symptoms (12 specific: Cramping<br>Feeling washed out, Light headedness, Restless legs, Nausea, Vomiting, Headache, Tingling Thirst, Shortness of breath Chest pain, Heart palpitations Itching, 1 free text type in); time to recovery question

For peer review only

****System identified only – no survey or interview data included in evidence synthesis**				
"Derby Evaluation of Illness" (44)	United Kingdom<br>Single renal unit<br><br>n=43 (19 HD, 5 PD, and 19 CKD)<br><br>CKD stages 4/5 pre-dialysis, in-centre HD, PD	Did not participate in survey<br><br><br>Home	Unknown – feasibility study reported<br>Daily (no reminders)	**New Study specific measure – unnamed (44)**<br>Six separate domains assessed by visual analogue scale (VAS): general wellbeing, pain, sleep, breathing, energy, fistula function and appetite.
EMPATHY study (45-48)	Canada<br>Three provincial regions<br><br>n=approx. 3,000<br><br>In-centre HD	Did not participate in survey<br><br>Questionnaires administered in-centre (precise timing not specified). PROMs are administered by pen and paper or through an electronic platform on a tablet in accordance with HD unit resources	Yes – Cluster RCT in progress<br>2 monthly	**ESAS-r: Renal (21)**<br>As above<br><br>**IPOS-Renal (7)**<br>As above<br><br>**And/or**<br>**EQ-5D-5L (8)**<br>As above<br>.
K-Pal (49)	United States of America<br>Single dialysis unit<br><br>n=22<br><br>Patient's ≥60 years of age with ESRD on HD	Did not participate in survey<br><br>During dialysis session (precise timing not specified)	Unknown – feasibility study reported<br>Baseline, 3 & 6 monthly	**Short-Form McGill Pain Questionnaire 2 (SF-MPQ-2)(50)**<br>The pain rating index has 2 subscales: Sensory subscale with 11 words, and affective subscale with 4 words from the original MPQ. Also one item for present pain intensity and one item for a 10 cm visual analogue scale (VAS) for average pain.[3] The SF-MPQ-2 includes 7 additional symptoms related to neuropathic pain, for a total of 22 items with 0-10 numerical response options<br><br>**Patient Health Questionnaire-9 (PHQ-9) (51)**<br>9 item questionnaire To measure depression severity and to diagnose depressive disorders

				**Generalized Anxiety Disorder 7 Item Survey (GAD-7) (52)**<br>7 item questionnaire exploring generalised anxiety<br><br>**Dialysis Symptom Index (DSI) (53)**<br>A 30-item measure designed to assess symptom prevalence and severity in patients on haemodialysis. 21 items relate to physical symptoms, 9 to emotional symptoms, plus open-ended question to report additional symptoms<br><br>**Kidney Disease Quality of Life (KDQOL-36) (19, 20)**<br>As above
eNephro (54)	France<br>Five Hospitals, 3 not for profit providers<br><br>Target stage 3B/4 (n = 320); stage 5D CKD on dialysis (n = 260); stage 5 T CKD treated with transplantation (n= 260).<br><br>CKD stage 3B/4, stage 5D CKD on dialysis (PD/HD), Transplant	Did not participate in survey<br><br>Home tele-monitoring	Unknown – Pragmatic RCT (anticipated date of study completion Dec 2018 NCTO 2082093)<br>Patient assessed symptoms reported every 3 days	**Patient assessed presence of symptoms** including vertigo, short breath, fatigue, oedema, abdominal pain, deterioration of general health status<br><br>PROs to assess study outcomes:<br>**KDQOL-36** (19, 20)<br>As above<br><br>**Hospitalization Anxiety Depression Scale (HADS)** (55)<br>14 questions in total (seven covering depression and seven covering anxiety).<br><br>**ReTransQoL (for transplant patients)(56)**<br>A 45-item measure designed to assess QOL in renal transplant patients. There are 5 dimensions: (i) physical health (ii) social functioning (iii) medical care (iv) treatment (v) fear of losing graft
Dutch Renal Registry (57-59)	The Netherlands<br><br>Collection via national registry pilot study in 16 centres<br><br>Advanced CKD with and without renal replacement therapy	Did not participate in the survey<br><br>Home<br>During dialysis session (precise timing not specified)	Yes – part of development of national registry of PROMs<br>Baseline, 3 & 6 months during study period	**Dialysis Symptom Index (53)**<br>As above<br><br>**SF-12 (60)**<br>A generic HRQOL questionnaire: 12 items to measure HRQOL. Domains: Physical functioning (PF) (2 items), Role physical (RP) (2 items), Bodily pain (BP) (1 item), General health (GH) (1 item) Vitality (VT) (1 item) Social functioning (SF) (1 item) Role emotional (RE) (2 items) Mental health (MH) (2 items)

References

1. Duncanson E, Bennett PN, Viecelli A, Dansie K, Handke W, Tong A, et al. Feasibility and acceptability of e-PROMs data capture and feedback among patients receiving haemodialysis in the Symptom monitoring WIth Feedback Trial (SWIFT) pilot: protocol for a qualitative study in Australia. BMJ Open.10(11):e039014.
2. Morton RL, Lioufas N, Dansie K, Palmer SC, Jose MD, Raj R, et al. Use of patient-reported outcome measures and patient-reported experience measures in renal units in Australia and New Zealand: A cross-sectional survey study. Nephrology. 2020;25(1):14-21.
3. Viecelli AK, Duncanson E, Bennett PN, D'Antoine M, Dansie K, Handke W, et al. Perspectives of Patients, Nurses, and Nephrologists About Electronic Symptom Monitoring With Feedback in Hemodialysis Care. Am J Kidney Dis. 2022;80(2):215-26.
4. Greenham L, Bennett PN, Dansie K, Viecelli AK, Jesudason S, Mister R, et al. The Symptom Monitoring with Feedback Trial (SWIFT): protocol for a registry-based cluster randomised controlled trial in haemodialysis. Trials. 2022;23(1):419.
5. Duncanson EB, P. N.; Viecelli, A. K.; Dansie, K.; Greenham, L. M.; Tong, A.; Jesudason, S.; McDonald, S. P.; Morton, R. L. Mini Orals: Feasibility and acceptability of electronic patient reported outcome measures (e-proms) data capture in the symptom monitoring with feedback trial (SWIFT) pilot: Perspectives of nephrologists, nurses and patients. Nephrology. 2020;25:35.
6. Ju A, Teixeira-Pinto A, Tong A, Smith AC, Unruh M, Davison SN, et al. Validation of a Core Patient-Reported Outcome Measure for Fatigue in Patients Receiving Hemodialysis. The SONG-HD Fatigue Instrument. 2020;15(11):1614-21.
7. Raj R, Ahuja K, Fr, sen M, Murtagh FE, Jose M. Validation of the IPOS-renal symptom survey in advanced kidney disease: A cross-sectional study. Journal of Pain and Symptom Management. 2018;56(2):281-7.
8. Devlin NJ, Brooks R. EQ-5D and the EuroQol Group: Past, Present and Future. Appl Health Econ Health Policy. 2017;15(2):127-37.
9. Perry LM, Morken V, Peipert JD, Yanez B, Garcia SF, Barnard C, et al. Patient-Reported Outcome Dashboards Within the Electronic Health Record to Support Shared Decision-making: Protocol for Co-design and Clinical Evaluation With Patients With Advanced Cancer and Chronic Kidney Disease. JMIR Res Protoc. 2022;11(9):e38461.
10. Schick-Makaroff K, Molzahn A. Brief Communication: patient satisfaction wit the use of tablet computers: a pilot study in two outpatient home dialysis clinics. Canadian Journal of Kidney Health and Disease. 2014;1(22).
11. Schick-Makaroff K, Molzahn AE. Evaluation of real-time use of electronic patient-reported outcome data by nurses with patients in home dialysis clinics. BMC Health Serv Res. 2017;17(1):439.

For peer review only

12. Schick-Makaroff K, Mharapara P. Use of Electronic Patient-Reported Outcomes in the Care of Patients with Kidney Failure. Nephrology Nursing Journal: Journal of the American Nephrology Nurses' Association. 2020;47(5):465-72.
13. Schick-Makaroff K, Molzahn A. Strategies to use tablet computers for collection of electronic patient-reported outcomes. Health & Quality of Life Outcomes. 2015;13:2.
14. Schick-Makaroff K, Tate K, Molzahn A. Use of Electronic Patient Reported Outcomes in Clinical Nephrology Practice: A Qualitative Pilot Study. Can J Kidney Health Dis. 2019;6:2054358119879451.
15. Silbernagel P, Sawatzky R, Klarenbach S, Czupryn J, Iradukunda R, Palfreyman S, et al. Patient-Reported Outcomes in Clinical Kidney Practice: Development of Education Sessions for Clinicians. Journal of Pain and Symptom Management. 2018;56(6).
16. Schick-Makaroff K, Sawatzky R, Kwon JY, Lee L, Cohen SR, Czupryn J, et al. A process evaluation in home dialysis: Electronic patient reported outcomes in clinical kidney practice (ePRO Kidney). Quality of Life Research. 2022;31(Supplement 2):S67.
17. Glasgow RE, Wagner EH, Schaefer J, Mahoney LD, Reid RJ, Greene SM. Development and validation of the Patient Assessment of Chronic Illness Care (PACIC). Med Care. 2005;43(5):436-44.
18. Seo AR, Kim BK, Park KS. Psychometric Properties and Effects on Health Outcomes of the Patient Assessment of Chronic Illness Care (PACIC) in Korean Hemodialysis Patients. Healthcare. 2022;10(6):20.
19. Chao S, Yen M, Lin T-C, Sung J-M, Wang M-C, Hung S-Y. Psychometric Properties of the Kidney Disease Quality of Life–36 Questionnaire (KDQOL-36™). Western Journal of Nursing Research. 2016;38(8):1067-82.
20. Peipert JD, Bentler PM, Klicko K, Hays RD. Psychometric Properties of the Kidney Disease Quality of Life 36-Item Short-Form Survey (KDQOL-36) in the United States. Am J Kidney Dis. 2018;71(4):461-8.
21. Tang E, Dano S, Edwards N, Macanovic S, Ford H, Bartlett S, et al. Screening for symptoms of anxiety and depression in patients treated with renal replacement therapy: utility of the Edmonton Symptom Assessment System-Revised. Qual Life Res. 2021;31(2):597-605.
22. Van Der Veer SN, Ercia A, Caskey FJ, Farrington K, Jury F, Rees M, et al. Developing an Intervention to Implement Electronic Patient-Reported Outcomes in Renal Services in the UK. Studies in Health Technology & Informatics. 2020;270:936-40.
23. Knowles SE, Ercia A, Caskey F, Rees M, Farrington K, Van der Veer SN. Participatory co-design and normalisation process theory with staff and patients to implement digital ways of working into routine care: the example of electronic patient-reported outcomes in UK renal services. BMC Health Serv Res. 2021;21(1):706.
24. van der Veer SN, Couchoud C, Morton RL. The role of kidney registries in expediting large-scale collection of patient-reported outcome measures for people with chronic kidney disease. Clin Kidney J. 2021;14(6):1495-503.
25. van der Veer SN, Aresi G, Gair R. Incorporating patient-reported symptom assessments into routine care for people with chronic kidney disease. Clinical Kidney Journal. 2017;10(6):783-7.

For peer review only

26. Gutierrez-Sanchez D, Leiva-Santos JP, Sanchez-Hern, ez R, Hern, ez-Marrero D, et al. Spanish modified version of the palliative care outcome scale-symptoms renal: cross-cultural adaptation and validation. BMC Nephrology. 2016;17(1):180.
27. Kyte D, Anderson N, Auti R, Aiyegbusi OL, Bishop J, Bissell A, et al. Development of an electronic patient-reported outcome measure (ePROM) system to aid the management of patients with advanced chronic kidney disease. J Patient Rep Outcomes. 2020;4(1):55.
28. Aiyegbusi OL, Kyte D, Cockwell P, Marshall T, Dutton M, Slade A, et al. Using Patient-Reported Outcome Measures (PROMs) to promote quality of care and safety in the management of patients with Advanced Chronic Kidney disease (PRO-trACK project): a mixed-methods project protocol. BMJ Open.7(6):e016687.
29. Kyte D, Anderson N, Bishop J, Bissell A, Brettell E, Calvert M, et al. Results of a pilot feasibility randomised controlled trial exploring the use of an electronic patient-reported outcome measure in the management of UK patients with advanced chronic kidney disease. BMJ Open. 2022;12(3):e050610.
30. Kyte D, Bishop J, Brettell E, Calvert M, Cockwell P, Dutton M, et al. Use of an electronic patient-reported outcome measure in the management of patients with advanced chronic kidney disease: the RePROM pilot trial protocol. BMJ Open. 2018;8(10):e026080.
31. Aiyegbusi OL, Kyte D, Cockwell P, Marshall T, Dutton M, Walmsley-Allen N, et al. Development and usability testing of an electronic patient-reported outcome measure (ePROM) system for patients with advanced chronic kidney disease. Computers in Biology & Medicine.101:120-7.
32. Wong D, Cao S, Ford H, Richardson C, ice, Belenko D, et al. Exploring the use of tablet computer-based electronic data capture system to assess patient reported measures among patients with chronic kidney disease: a pilot study. BMC Nephrology. 2017;18:1-10.
33. Mucsi I, Chawla G, Chen A, Sanchez MJPM, Edwards N, Peipert JD, et al. Construct validity of the patient-reported outcomes measurement information system (promis) profile summary scores in patients with kidney failure. Journal of the American Society of Nephrology. 2021;32:632.
34. Evans JM, Glazer A, Lum R, Heale E, Mackinnon M, Blake PG, et al. Implementing a patient-reported outcome measure for hemodialysis patients in routine clinical care perspectives of patients and providers on esas-r:Renal. Clinical Journal of the American Society of Nephrology. 2020;15(9):1299-309.
35. Schell JO, Lupu DE. A step in the right direction the promise of proms in routine hemodialysis care. Clinical Journal of the American Society of Nephrology. 2020;15(9):1228-30.
36. Grove BE, Ivarsen P, de Thurah A, Schougaard LM, Kyte D, Hjollund NH. Remote follow-up using patient-reported outcome measures in patients with chronic kidney disease: the PROKID study - study protocol for a non-inferiority pragmatic randomised controlled trial. BMC Health Serv Res. 2019;19(1):631.

For peer review only

37. Grove BE, Schougaard LMV, Ivarsen PR, Kyte D, Hjollund NH, de Thurah A. Patient-reported outcome measures for clinical decision-making in outpatient follow-up: validity and reliability of a renal disease questionnaire. Journal of Patient-Reported Outcomes. 2021;5(1):1-11.
38. Schougaard L M LLP, Jessen A et al. AmbuFlex: tele-patient-reported outcomes (telePRO) as the basis for follow up in chronic and malignant diseases. Qual Life Res. 2016;25(3):525-34.
39. Hjollund NHI, Larsen LP, de Thurah AL, Grove BE, Skuladottir H, Linnet H, et al. Patient-reported outcome (PRO) measurements in chronic and malignant diseases: ten years' experience with PRO-algorithm-based patient-clinician interaction (telePRO) in AmbuFlex. Qual Life Res. 2023.
40. Grove BE, Valen Schougaard LM, Ivarsen P, Hjollund NH, de Thurah A, Mejdahl CT. Remote follow-up based on patient-reported outcomes in patients with chronic kidney disease: A qualitative study of patient perspectives. PLoS One. 2023;18(2):e0281393.
41. Grove B, Ivarsen P, De Thurah A, Kyte D, Henrik HN. Tele follow-up using patient-reported outcomes (PRO) measures in patients with chronic kidney disease-the PRO-KID study: A study protocol for a non-inferiority randomised controlled trial in Denmark. Quality of Life Research.27:S143-S4.
42. Flythe JE, Dorough A, Narendra JH, Wingard RL, Dalrymple LS, DeWalt DA. Development and content validity of a hemodialysis symptom patient-reported outcome measure. Qual Life Res. 2019;28(1):253-65.
43. Flythe JE, Tugman MJ, Narendra JH, Dorough A, Hilbert J, Assimon MM, et al. Feasibility of Tablet-Based Patient-Reported Symptom Data Collection Among Hemodialysis Patients. Kidney Int Rep. 2020;5(7):1026-39.
44. Pittman ZC, John SG, McIntyre CW. Collection of daily patient reported outcomes is feasible and demonstrates differential patient experience in chronic kidney disease. Hemodial Int. 2017;21(2):265-73.
45. Schick-Makaroff K, Wozniak LA, Short H, Davison SN, Klarenbach S, Buzinski R, et al. Burden of mental health symptoms and perceptions of their management in in-centre hemodialysis care: a mixed methods study. J Patient Rep Outcomes. 2021;5(1):111.
46. Johnson JA, Al Sayah F, Buzinski R, Corradetti B, Davison SN, Elliott MJ, et al. A cluster randomized controlled trial for the Evaluation of routinely Measured PATient reported outcomes in HemodialYsis care (EMPATHY): a study protocol. BMC Health Services Research. 2020;20(1):1-14.
47. Davison SN, Klarenbach S, Manns B, Schnick-Makaroff K, Buzinski R, Corradetti B, et al. Patient-reported outcome measures in the care of in-centre hemodialysis patients. Journal of Patient-Reported Outcomes. 2021;5(1):1-8.
48. Schick-Makaroff K, Wozniak LA, Short H, Davison SN, Klarenbach S, Buzinski R, et al. How the Routine Use of Patient-Reported Outcome Measures for Hemodialysis Care Influences Patient-Clinician Communication: A Mixed-Methods Study. Clin J Am Soc Nephrol. 2022;17(11):1631-45.

For peer review only

49. Gabbard J, McLouth CJ, Brenes G, Claudel S, Ongchuan S, Burkart J, et al. Rapid Electronic Capturing of Patient-Reported Outcome Measures in Older Adults With End-Stage Renal Disease: A Feasibility Study. Am J Hosp Palliat Care. 2021;38(5):432-40.
50. Dworkin RH, Turk DC, Revicki DA, Harding G, Coyne KS, Peirce-Sandner S, et al. Development and initial validation of an expanded and revised version of the Short-form McGill Pain Questionnaire (SF-MPQ-2). Pain. 2009;144(1-2):35-42.
51. Kroenke K, Spitzer RL, Williams JB. The PHQ-9: validity of a brief depression severity measure. J Gen Intern Med. 2001;16(9):606-13.
52. Spitzer RL, Kroenke K, Williams JBW, Löwe B. A Brief Measure for Assessing Generalized Anxiety Disorder: The GAD-7. Archives of Internal Medicine. 2006;166(10):1092-7.
53. Weisbord SD, Fried LF, Arnold RM, Rotondi AJ, Fine MJ, Levenson DJ, et al. Development of a symptom assessment instrument for chronic hemodialysis patients: the Dialysis Symptom Index. Journal of Pain & Symptom Management. 2004;27(3):226-40.
54. Thilly N, Chanliau J, Frimat L, Combe C, Merville P, Chauveau P, et al. Cost-effectiveness of home telemonitoring in chronic kidney disease patients at different stages by a pragmatic randomized controlled trial (eNephro): rationale and study design. BMC Nephrology. 2017;18(1):126.
55. Snaith RP. The Hospital Anxiety And Depression Scale. Health and Quality of Life Outcomes. 2003;1(1):29.
56. Gentile S, Jouve E, Dussol B, Moal V, Berland Y, Sambuc R. Development and validation of a French patient-based health-related quality of life instrument in kidney transplant: the ReTransQoL. Health Qual Life Outcomes. 2008;6:78.
57. van der Willik EM, Hemmelder MH, Bart HAJ, van Ittersum FJ, Hoogendijk-van den Akker JM, Bos WJW, et al. Routinely measuring symptom burden and health-related quality of life in dialysis patients: first results from the Dutch registry of patient-reported outcome measures. Clin Kidney J. 2021;14(6):1535-44.
58. van der Willik EM, Meuleman Y, Prantl K, van Rijn G, Bos WJW, van Ittersum FJ, et al. Patient-reported outcome measures: selection of a valid questionnaire for routine symptom assessment in patients with advanced chronic kidney disease - a four-phase mixed methods study. BMC Nephrol. 2019;20(1):344.
59. van der Willik EM, Milders J, Bart JAJ, Bos WJW, van Ittersum FJ, Ten Dam M, et al. Discussing results of patient-reported outcome measures (PROMs) between patients and healthcare professionals in routine dialysis care: a qualitative study. BMJ Open. 2022;12(11):e067044.
60. Ware J, Jr., Kosinski M, Keller SD. A 12-Item Short-Form Health Survey: construction of scales and preliminary tests of reliability and validity. Med Care. 1996;34(3):220-33.

For peer review only

Example illustrative quotations

**Intervention characteristics**<br>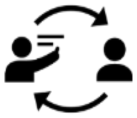	*'huge variety of different PROMs available but no single PROM measured even 50% of the symptoms that were reported by the population. So I think that kind of sends a clear message that maybe there's a gap here for a PROM that's maybe a bit more comprehensive that meets patient's needs. But of course, the challenge of that is you don't want to make it too long or burdensome.' (01) –* **Heterogeneity of measures**<br><br>*'the main learning is that if we want to make ePRO collection a success in renal or in any other specialty, we need to find concrete decisions or concrete situations where ePRO information really adds value. My learning is that just coming ePROs as a concept and saying, 'Now we're going to collect this data for anything,' I think that's not the way to go '(09).* **Importance of context and purpose**<br><br>*'It's a huge investment to make ePROS part of a registry and create that extra platform, and so we need to really provide the evidence that it's going to work before we just outlay everything, don't we?' (010)* **– Evidence of impact & benefit**
**Outer setting**<br>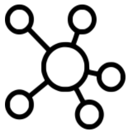	*'The way we designed the study was that we had intentionally iterations planned. We start with a certain package, then look at what works and what doesn't work and then add stuff. So related to what I said at the beginning, I think we started off with focusing on the patient side of things but acknowledging that we would need to do something to improve clinician engagement later on. So, I think I've always envisaged that being part of the later iterations, but we never got there because the study got paused halfway through the second wave' (09)* **– Impact of COVID**<br><br>*'It's really like hospital IT department, we kind of conceived of them as like a vendor who we were purchasing their services to launch something, which I guess is good, really wanted to be full partners, but that means they brought in their whole ideas about what informatics kind of launches should be like and this stuff. Which they were included in the co-production from the very beginning, which was good, but that relationship still isn't totally as good as it could be and so that's a challenge too……. there's always sort of push and pull on the priorities (02) –* **Partnerships and competing interests**<br><br>*'we were really planning to use this existing framework because this platform is integrated and linked to the local EPR and so once patients complete the questionnaires, their scores are displayed for the team' (04) –* **Data linkage**
**Inner setting**<br>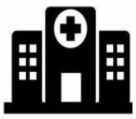	*'And that was one thing that we had thought of going into it, was that any time you have a new sort of feature that's going to be in the clinic, it can actually exacerbate disparities because the patients who are most ready to benefit from that, who are already doing the best probably will, right? And so now they've got this new thing that they can use and the patients that are already doing worse or have the most challenges or barriers, that's just another thing that they're not going to be able to take advantage of…' (02) –* **Accessibility and inclusivity**

	'I think giving the people in these units the opportunity to fill out a survey in their own language also then increases the inclusivity of the trial' (010) - **Accessibility and inclusivity**<br><br>*But they [patients] did use words like feeling empowered to be a partner in the discussion. And obviously bringing up things, they had permission to bring things up and so it would be easier to start a conversation on mental health for example '(012)* – **shifting power to patients**<br><br>*And if I was going to do it over again, I would have taken all the emphasis off the clinicians, and I would have put it on the patients. I would have educated the patients. I would have told them how they could use it. Initiating discussion with the clinician. Giving it to them to share with their clinician or, like I would have turned the tables (03*) **– shifting power to patients**<br><br>*'They [clinicians] felt that when you have the ePRO data in advance of a consultation it allowed them to focus in on the really important aspects around patient symptoms from the outset so maybe more efficient use of time' (01)* – **fitting into workflow**
**Characteristics of individuals**<br>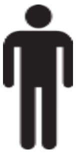	*'we also want to link the whole assessment to response pathways, both self-management support for patients and decision support for the professional teams' (04)* – **Ability to deal with responses**<br><br>*'But I think what I'm resisting is the, to quote Foucault, which is about the technology of self which is going to put it in a box. And say, this is not so bad, this is okay. This is a screaming alert and so the screaming alert gets all of our attention and all of sudden we've 'technologicalised' a life. And we don't want to do that so, I'm worried about moving to the technological simplification of something that isn't simple……….Yes, particularly in our context right, particularly where we see high technological dependence primarily in the nursing role…' (03)* – **Danger of digitising the patient experience**
**Process**<br>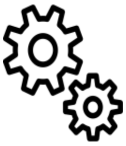	*'so the registry is a really important tool. We have this huge platform where we collect this information and it's already put in there in real time for the most part' (010)* – **Pragmatic approach**<br><br>*'The patient advisory group was really important especially during development. There are a number of times where we sort of went in with a plan and for the intervention, for the ePRO and we changed our plans based on what they were telling us in terms of how this would actually work in practice' (01)* – **Importance of patient involvement**<br><br>*'They got face-to-face training because they needed to understand the system really well and they needed to be able to explain what certain things meant. We did train nurses but that was more in groups. We maybe joined a multidisciplinary team meeting or a team meeting for the nurses in the morning, explaining where they could find the results' (09)* – **Value of training**

For peer review only

	'I guess the sustainability is the question because you can envision training and so-called patient [involvement] at the launch of this initiative and hoping that it will be cascaded down further and sustained but probably some kind of ongoing training or at least continuing education in services for nurses is going to be necessary. That's also part of a sustainability plan. It needs to become part of the quality systems (04) **– Sustainability**

For peer review only

COREQ (COnsolidated criteria for REporting Qualitative research) Checklist

A checklist of items that should be included in reports of qualitative research. You must report the page number in your manuscript where you consider each of the items listed in this checklist. If you have not included this information, either revise your manuscript accordingly before submitting or note N/A.

Topic	Item No.	Guide Questions/Description	Reported on Page No.
**Domain 1: Research team and reflexivity**			
Personal characteristics			
Interviewer/facilitator	1	Which author/s conducted the interview or focus group?	
Credentials	2	What were the researcher's credentials? E.g. PhD, MD	
Occupation	3	What was their occupation at the time of the study?	
Gender	4	Was the researcher male or female?	
Experience and training	5	What experience or training did the researcher have?	
Relationship with participants			
Relationship established	6	Was a relationship established prior to study commencement?	
Participant knowledge of the interviewer	7	What did the participants know about the researcher? e.g. personal goals, reasons for doing the research	
Interviewer characteristics	8	What characteristics were reported about the inter viewer/facilitator? e.g. Bias, assumptions, reasons and interests in the research topic	
**Domain 2: Study design**			
Theoretical framework			
Methodological orientation and Theory	9	What methodological orientation was stated to underpin the study? e.g. grounded theory, discourse analysis, ethnography, phenomenology, content analysis	
Participant selection			
Sampling	10	How were participants selected? e.g. purposive, convenience, consecutive, snowball	
Method of approach	11	How were participants approached? e.g. face-to-face, telephone, mail, email	
Sample size	12	How many participants were in the study?	
Non-participation	13	How many people refused to participate or dropped out? Reasons?	
Setting			
Setting of data collection	14	Where was the data collected? e.g. home, clinic, workplace	
Presence of non-participants	15	Was anyone else present besides the participants and researchers?	
Description of sample	16	What are the important characteristics of the sample? e.g. demographic data, date	
Data collection			
Interview guide	17	Were questions, prompts, guides provided by the authors? Was it pilot tested?	
Repeat interviews	18	Were repeat inter views carried out? If yes, how many?	
Audio/visual recording	19	Did the research use audio or visual recording to collect the data?	
Field notes	20	Were field notes made during and/or after the inter view or focus group?	
Duration	21	What was the duration of the inter views or focus group?	
Data saturation	22	Was data saturation discussed?	
Transcripts returned	23	Were transcripts returned to participants for comment and/or	

For peer review only

Topic	Item No.	Guide Questions/Description	Reported on Page No.
		correction?	
**Domain 3: analysis and findings**			
Data analysis			
Number of data coders	24	How many data coders coded the data?	
Description of the coding tree	25	Did authors provide a description of the coding tree?	
Derivation of themes	26	Were themes identified in advance or derived from the data?	
Software	27	What software, if applicable, was used to manage the data?	
Participant checking	28	Did participants provide feedback on the findings?	
Reporting			
Quotations presented	29	Were participant quotations presented to illustrate the themes/findings? Was each quotation identified? e.g. participant number	
Data and findings consistent	30	Was there consistency between the data presented and the findings?	
Clarity of major themes	31	Were major themes clearly presented in the findings?	
Clarity of minor themes	32	Is there a description of diverse cases or discussion of minor themes?	

Developed from: Tong A, Sainsbury P, Craig J. Consolidated criteria for reporting qualitative research (COREQ): a 32-item checklist for interviews and focus groups. *International Journal for Quality in Health Care*. 2007. Volume 19, Number 6: pp. 349 – 357

Once you have completed this checklist, please save a copy and upload it as part of your submission. DO NOT include this checklist as part of the main manuscript document. It must be uploaded as a separate file.

For peer review only
